# Supplementary material for: Engineering allorejection-resistant CAR-NKT cells from hematopoietic stem cells for off-the-shelf cancer immunotherapy
Source: Mol Ther. 2024 Apr 6;32(6):1849–74. doi: 10.1016/j.ymthe.2024.04.005 (PMC11184334; doi:10.1016/j.ymthe.2024.04.005)
Supplement: Document S1. Figures S1–S14 [file mmc1.pdf]

YMTHE, Volume *32*

Supplemental Information

Engineering allorejection-resistant CAR-NKT cells from hematopoietic stem cells for off-the-shelf cancer immunotherapy

Yan-Ruide Li, Yang Zhou, Jiaji Yu, Yichen Zhu, Derek Lee, Enbo Zhu, Zhe Li, Yu Jeong Kim, Kuangyi Zhou, Ying Fang, Zibai Lyu, Yuning Chen, Yanxin Tian, Jie Huang, Xinjian Cen, Tiffany Husman, Jae Min Cho, Tzung Hsiai, Jin J. Zhou, Pin Wang, Benjamin R. Puliafito, Sarah M. Larson, and Lili Yang

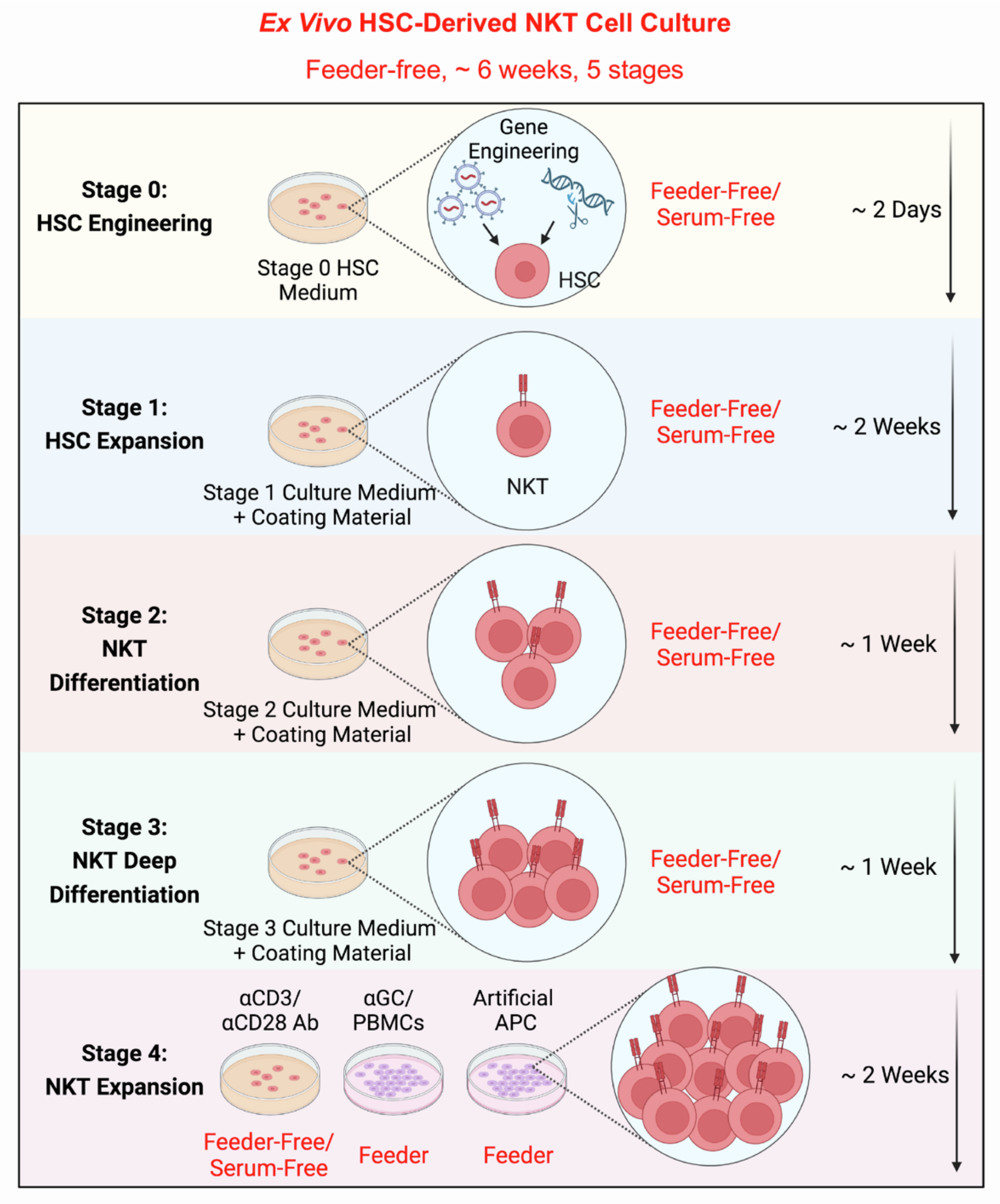

Figure S1. Diagram of the *Ex Vivo* HSC-Derived NKT Cell Culture.
Cryopreserved human cord blood-derived CD34$^+$ hematopoietic stem and progenitor cells (referred to as HSCs) were used to generate NKT cell products through a 5-stage, 6-week process. At Stage 0, HSCs were transduced with a lentivector, electroporated with a CRISPR-Cas9/B2M-CIITA-gRNAs complex, and then cultured over 48 hours in a classical X-VIVO 15-based HSC Medium. Gene-engineered HSCs were then cultured over ~6 weeks to generate a designated CAR-NKT cell product: Stage 1 HSC expansion (~2 weeks), Stage 2 NKT differentiation (~1 week), Stage 3 NKT deep differentiation (~1 week), and Stage 4 NKT expansion (~2 weeks). The Stage 1 Culture Medium comprised the StemSpan^{TM} SFEM II Medium (SFEM) and the StemSpan^{TM} Lymphoid Progenitor Expansion Supplement to support the HSC expansion. The Stage 2 Culture Medium comprised the SFEM and the StemSpan^{TM} Lymphoid Progenitor Maturation Supplement (LPMS) to support the NKT cell differentiation. The Stage 3 Culture Medium comprised the SFEM, the LPMS, the CD3/CD28/CD2 T Cell Activator, and the human

recombinant IL-15 to support NKT cell deep differentiation. In addition, the StemSpan™ Lymphoid Differentiation Coating Material were utilized throughout Stages 1 to 3 to support HSC expansion and differentiation into T cell lineage. The entire 5-stage culture can be implemented in a feeder-free and serum-free manner. Alternatively, two feeder-dependent strategies involving α-galactosylceramide (αGC)-loaded healthy donor PBMCs or K562-based artificial antigen-presenting cells (aAPCs) can be employed for Stage 4 NKT expansion.

A

Cell products	Engineering strategy		Description
	Lentivector design	CRISPR-Cas9 editing	
^{Allo15}BCAR-NKT	Lenti/iNKT-BCAR-IL-15	NA	Allogeneic IL-15-enhanced BCMA-targeting CAR-engineered NKT cells
^{U15}BCAR-NKT	Lenti/iNKT-BCAR-IL-15	*B2M* and *CIITA* KO	HLA-ablated universal IL-15-enhanced BCMA-targeting CAR-engineered NKT cells
^{Allo15}BCAR-NKT/FG	Lenti/iNKT-BCAR-IL-15 and Lenti/FG	NA	Allogeneic IL-15-enhanced BCMA-targeting CAR-engineered NKT cells labeled with FG
^{U15}BCAR-NKT/FG	Lenti/iNKT-BCAR-IL-15 and Lenti/FG	*B2M* and *CIITA* KO	HLA-ablated universal IL-15-enhanced BCMA-targeting CAR-engineered NKT cells labeled with FG
^{U}BCAR-NKT-TK	Lenti/iNKT-BCAR-sr39TK	*B2M* and *CIITA* KO	HLA-ablated universal BCMA-targeting CAR-engineered NKT cells with sr39TK
^{UE}BCAR-NKT	Lenti/iNKT-BCAR-HLA-E	*B2M* and *CIITA* KO	HLA-ablated universal HLA-E-overexpressed BCMA-targeting CAR-engineered NKT cells

B

Lentivectors	Titers (IFU/ml)
Lenti/iNKT-BCAR-IL-15	$1.784 \times 10^8 \pm 5.61 \times 10^7$
Lenti/iNKT-BCAR-sr39TK	$1.623 \times 10^8 \pm 2.49 \times 10^7$
Lenti/iNKT-BCAR-HLA-E	$1.692 \times 10^8 \pm 4.28 \times 10^7$

C

Cell products	VCN
^{Allo15}BCAR-NKT	3.40 ± 0.05
^{U15}BCAR-NKT	3.27 ± 0.13
^{UE}BCAR-NKT	3.19 ± 0.27

D

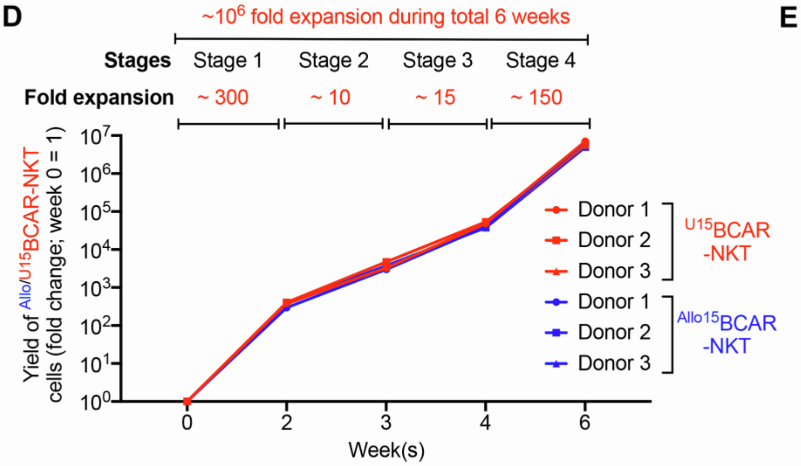

E

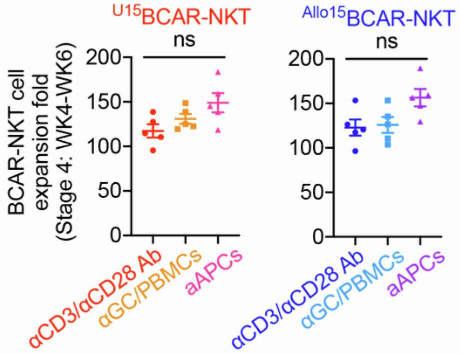

F

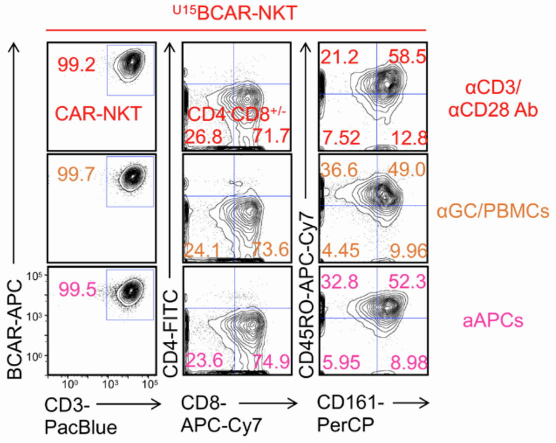

G

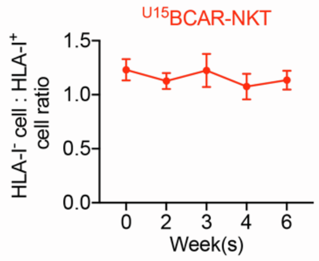

Figure S2. Summary of CAR-NKT cell products generated in this study and the generation of $^{Allo/U15}$BCAR-NKT cells.
(A) Table summarizing the generated CAR-NKT cell products in this study.
(B) Titers of the three lentivectors used in this study. Vector titers were measured by transducing HEK 293T cells with serial dilutions and performing flow cytometry (n = 3-6). IFU, infectious units.
(C) Vector copy numbers (VCNs) in the three indicated cell products. The generated BCAR-NKT cells were collected and analyzed for average VCN per cell using droplet digital PCR (ddPCR) (n = 3-6).
(D) Yield of $^{Allo/U15}$BCAR-NKT cells during the *Ex Vivo* HSC-Derived NKT Cell Culture.
(E) Comparison of $^{Allo/U15}$BCAR-NKT cell expansion fold using the three expansion approaches (n = 5).
(F) FACS detection of surface markers on ^{U15}BCAR-NKT cells generated using the three expansion approaches.
(G) The ratio of HLA-I/II-ablated ^{U15}BCAR-NKT cells to non-gene-edited cells during the *Ex Vivo* HSC-Derived NKT Cell Culture (n = 5).
Representative of > 3 experiments. Data are presented as the mean ± SEM. ns, not significant, by one-way ANOVA.

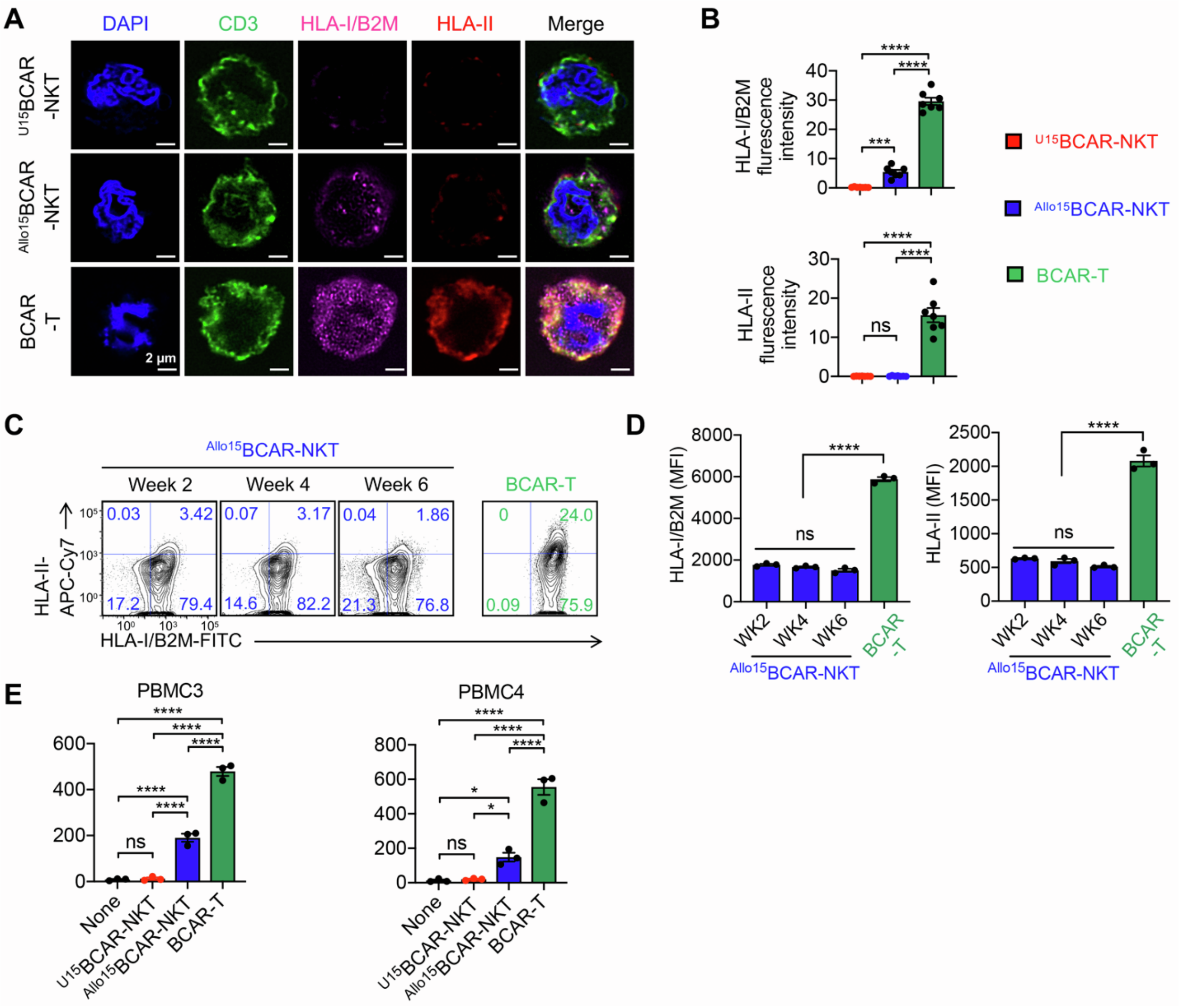

Figure S3. U15BCAR-NKT cells display an HLA-negative phenotype and resist to T cell-mediated allorejection, related to Figure 3.

(A-B) Studying HLA expression on Allo/U15BCAR-NKT cells. (A) Immunofluorescence analysis of surface HLA-I/II on Allo/U15BCAR-NKT cells. (B) Quantification of (A) (n = 7).

(C-D) Studying HLA expression Allo15BCAR-NKT cells throughout the 6-week cell culture. (C) FACS measurements of surface HLA-I/II molecules on Allo15BCAR-NKT cells collected from the indicated weeks. Conventional BCAR-T cells were included as a staining control. (D) Quantification of (C) (n = 3).

(E) ELISA analyses of IFN-γ production on day 4 in the T cell-mediated allorejection MLR assays (n = 3).

Representative of 3 experiments. Data are presented as the mean ± SEM. ns, not significant, *p < 0.05, ***p < 0.001, ****p < 0.0001, by one-way ANOVA.

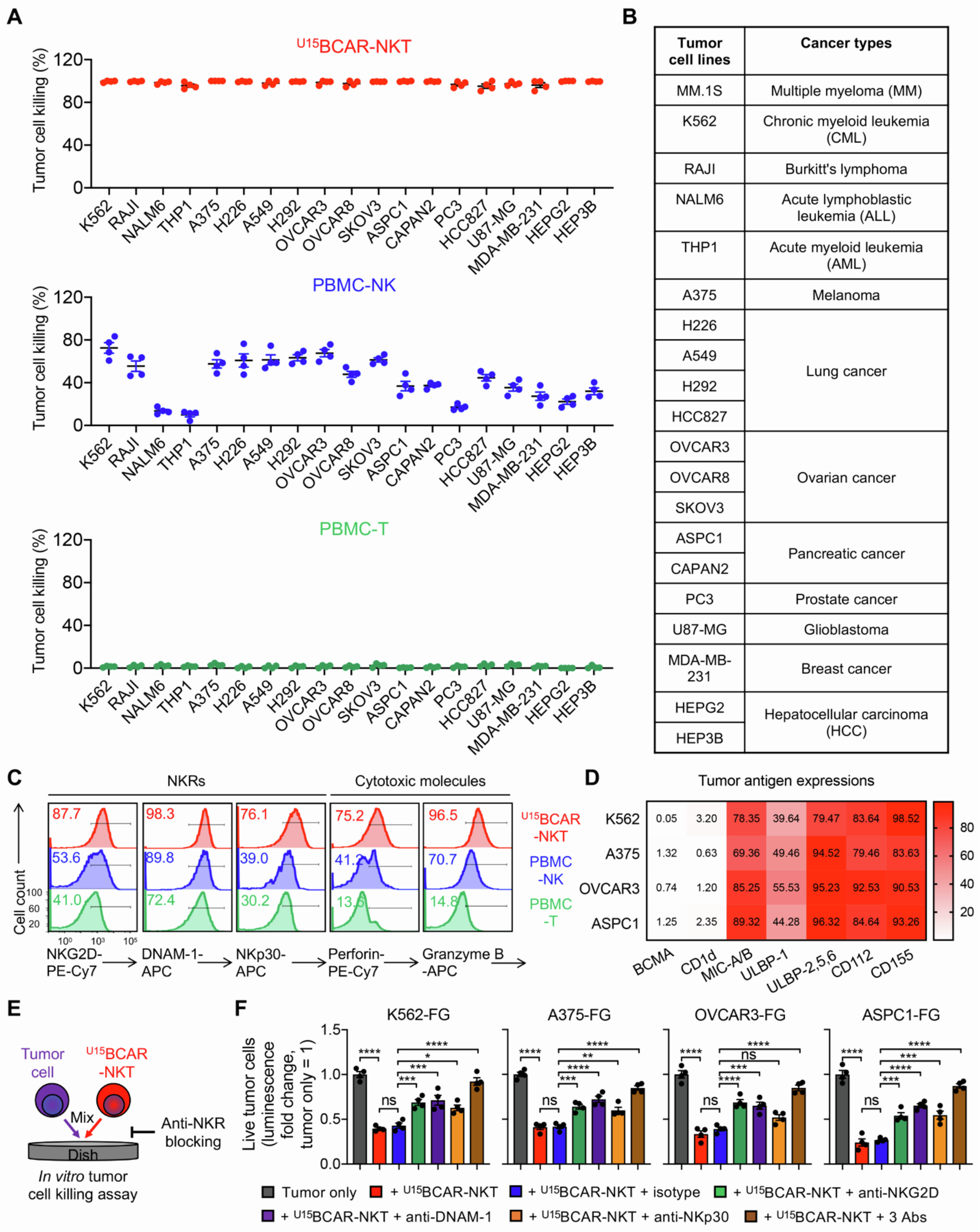

Figure S4. *In vitro* tumor targeting of U15BCAR-NKT cells through intrinsic NK function, related to Figure 4.

(A) Tumor cell killing data of ^{U15}BCAR-NKT cells at 24 h (n = 4). Healthy donor peripheral blood mononuclear cell (PBMC)-derived NK (PBMC-NK) and conventional T (PBMC-T) cells are included as therapeutic cell controls. E:T ratio = 10:1.
(B) Summary of tumor cell lines used in this study.
(C) FACS detection of surface NKR expression and intracellular cytotoxic molecules production by the indicated cells.
(D) FACS analyses of surface tumor antigen expressions on the indicated tumor cell lines. Data from 4 tumor cell lines (i.e., K562, A375, OVCAR3, and ASPC1) are presented. The numbers in the heatmap represent the percentage of antigen-positive tumor cells relative to the total tumor cell population. These percentages were determined based on control isotype staining.
(E-F) Studying the tumor killing mechanism of ^{U15}BCAR-NKT cells mediated by NKRs (i.e., NKG2D, DNAM-1, and NKp30). (E) Experimental design. (F) Tumor cell killing data at 24 h (E:T ratio = 2:1; n = 4).
Representative of 3 experiments. Data are presented as the mean ± SEM. ns, not significant, *p < 0.05, **p < 0.01, ***p < 0.001, ****p < 0.0001, by 1-way ANOVA.

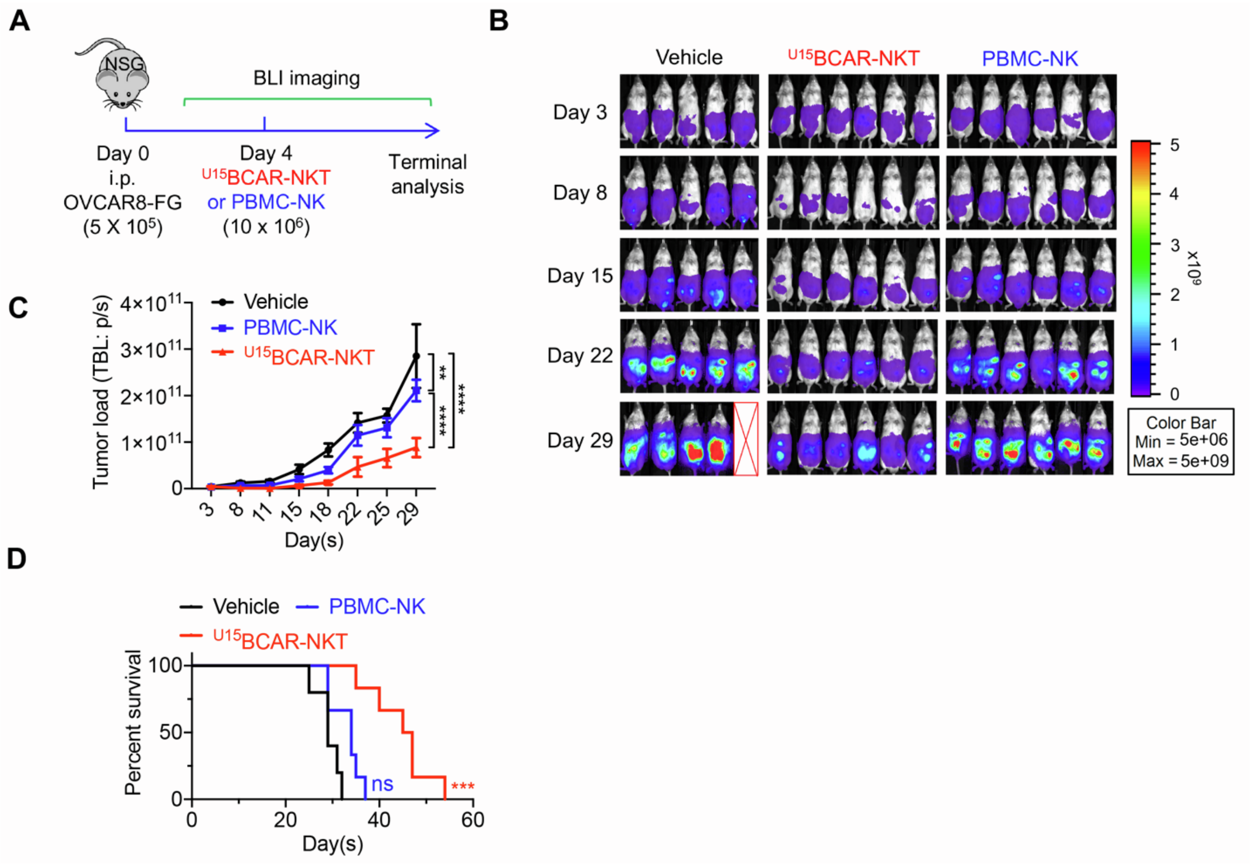

Figure S5. ***In vivo*** **tumor targeting of** **^{U15}BCAR-NKT cells through intrinsic NK function, related to Figure 4.**

(A) Experimental design to study the *in vivo* antitumor efficacy of ^{U15}BCAR-NKT cells in an OVCAR8-FG xenograft NSG mouse model. PBMC-NK cells were included as a therapeutic cell control.

(B) BLI images showing tumor loads in experimental mice over time.

(C) Quantification of (B) (n = 5-6).

(D) Kaplan-Meier survival curves of experimental mice over time (n = 5-6).

Representative of 3 experiments. Data are presented as the mean ± SEM. ns, not significant, **p < 0.01, ***p < 0.001, ****p < 0.0001, by 2-way ANOVA (C), or log rank (Mantel-Cox) test adjusted for multiple comparisons (D).

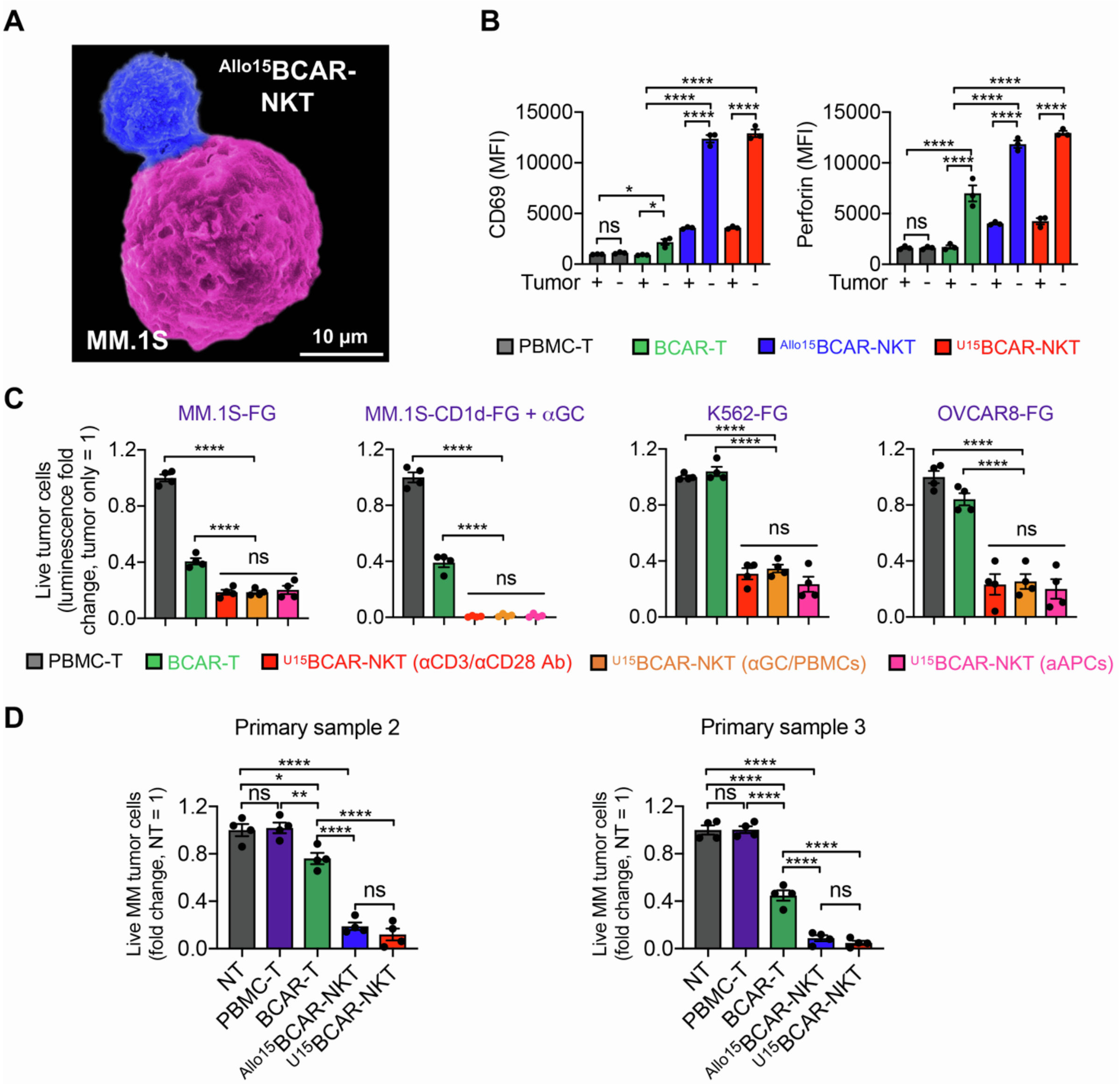

Figure S6. ***In vitro*** **antitumor efficacy and mechanism of action (MOA) study of U15BCAR-NKT cells, related to Figure 4.**

(A) Scanning electron microscope (SEM) image showing that an Allo15BCAR-NKT cell (blue) is attacking an MM.1S tumor cell (purple).

(B) FACS analyses of surface activation marker (i.e., CD69) and intracellular cytotoxic molecule (i.e., Perforin) in the indicated cells after coculturing with MM.1S-FG cells for 24 hours (n = 3).

(C) Tumor cell killing data by the indicated therapeutic cells at 24 h (E:T ratio = 1:1; n = 4). U15BCAR-NKT cells generated using the three expansion approaches were compared.

(D) Primary MM tumor cell killing data by the indicated cells at 24 h (n = 4).

Representative of 3 experiments. Data are presented as the mean ± SEM. ns, not significant, *p < 0.05, **p < 0.01, ***p < 0.001, ****p < 0.0001, by 1-way ANOVA.

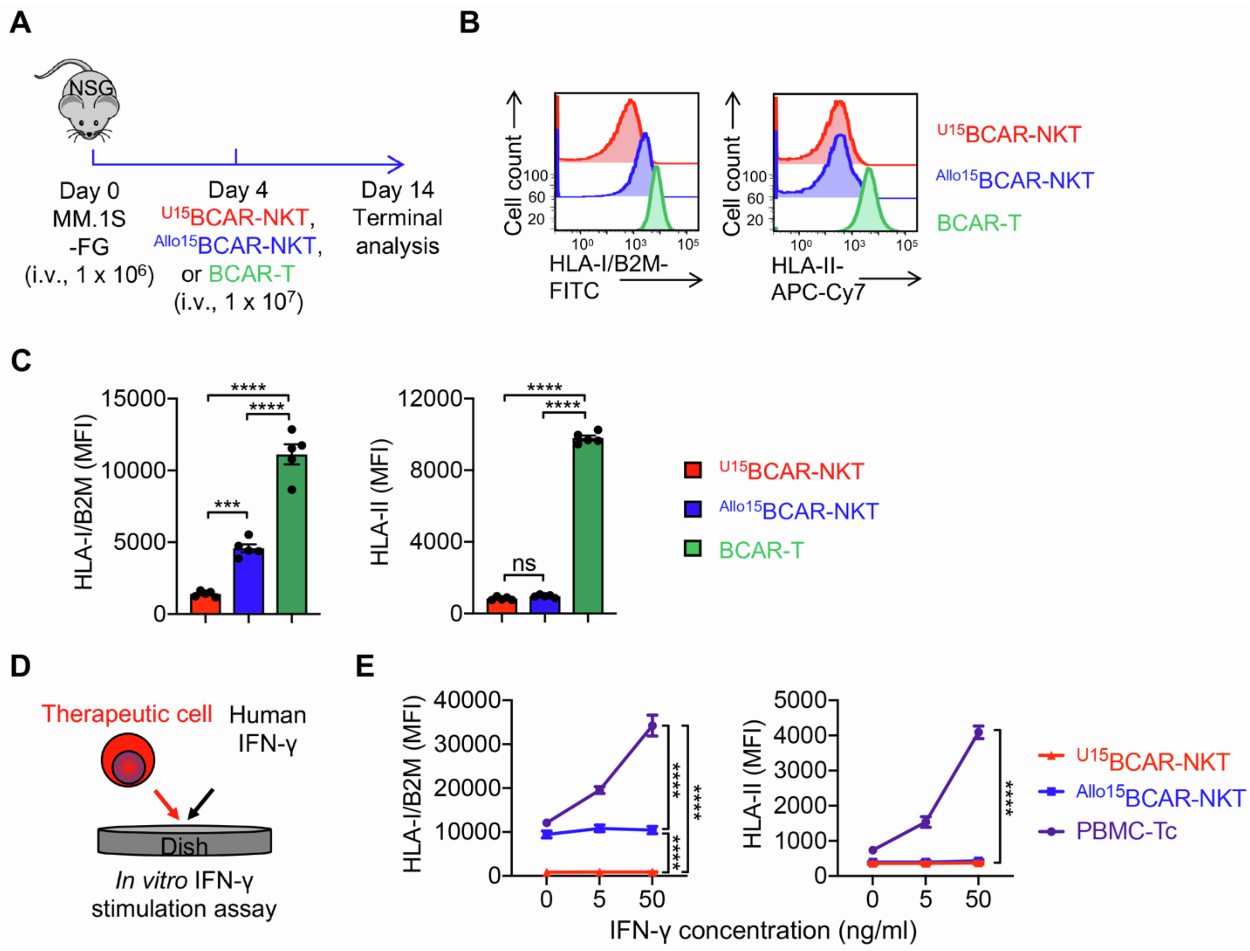

Figure S7. HLA expression of $^{Allo/U15}$BCAR-NKT cells *in vivo* or under IFN-γ stimulation, related to Figure 5.

(A-C) Studying HLA expression of $^{Allo/U15}$BCAR-NKT cells in a human MM.1S xenograft NSG mouse model. (A) Experimental design. Conventional BCAR-T cells were included as a control. (B) FACS measurements of surface HLA-I/II on $^{Allo/U15}$BCAR-NKT cells post *in vivo* antitumor response. (C) Quantification of (B) (n = 5).

(D-E) Studying HLA expression of $^{Allo/U15}$BCAR-NKT cells under IFN-γ stimulation. (D) Experimental design. $^{Allo/U15}$BCAR-NKT cells were stimulated with a range of IFN-γ (0, 5, and 50 ng/ml) for 3 days. PBMC-Tc cells were included as a positive control. (E) Quantification of HLA-I/II expressions on the indicated cells stimulated with IFN-γ of indicated concentrations (n = 3).

Representative of 3 experiments. Data are presented as the mean ± SEM. ns, not significant, ***p < 0.001, ****p < 0.0001, by 1-way ANOVA (C) or 2-way ANOVA (E).

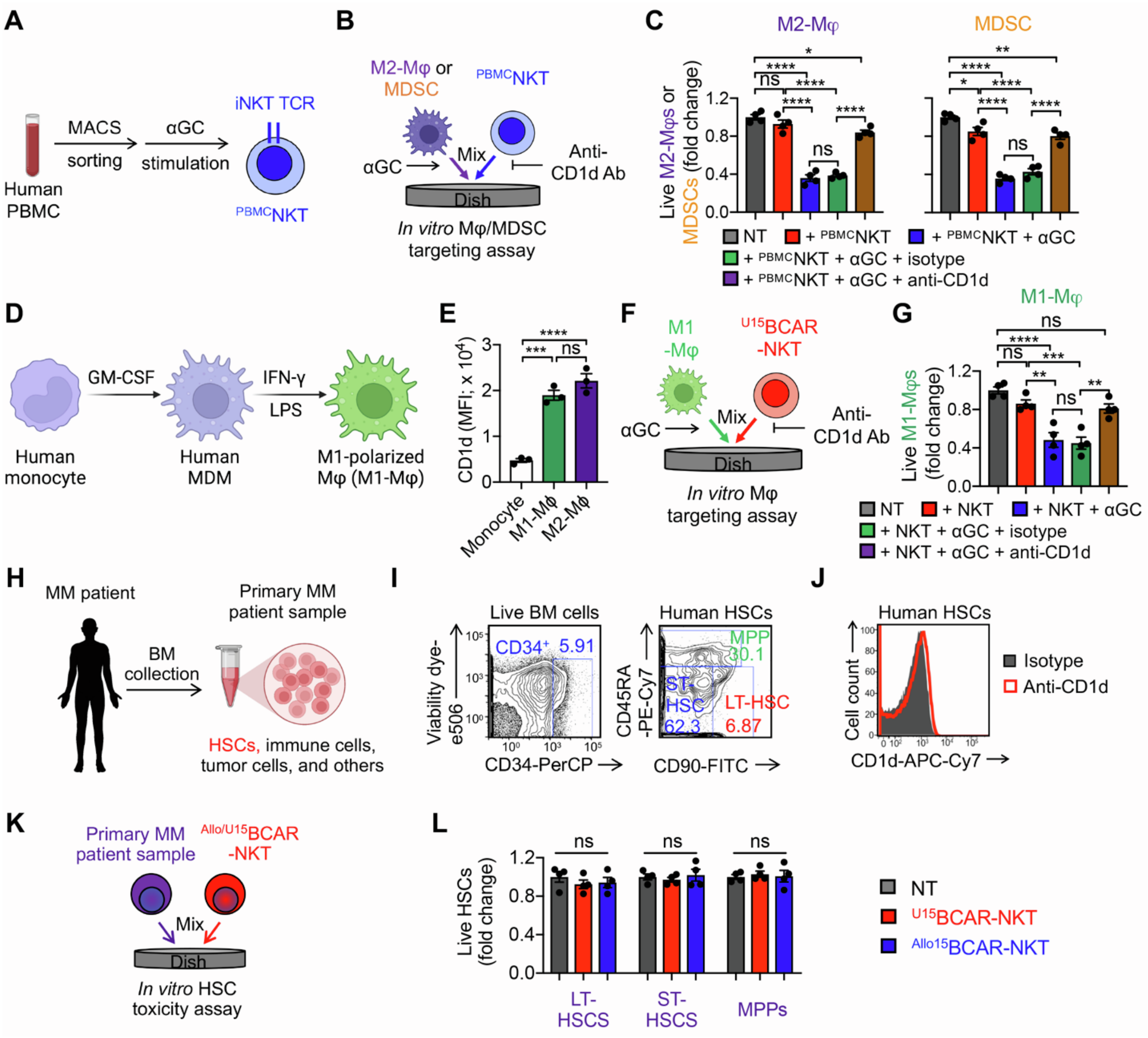

Figure S8. $^{Allo/U15}$BCAR-NKT cells selectively target the TME but not affect the human HSCs, related to Figure 7.

(A-C) Studying the TAM/MDSC targeting by PBMC-derived NKT (^{PBMC}NKT) cells. (A) Schematics showing the generation of ^{PBMC}NKT cells. (B) Experimental design to study Mφ/MDSC targeting by ^{PBMC}NKT cells using an *in vitro* Mφ/MDSC targeting assay. Mφ/MDSC:^{PBMC}NKT ratio = 1:1. (C) Mφ/MDSC killing data at 24 h (n = 4).

(D-G) Studying the M1-type macrophage (M1-Mφ) targeting by $^{Allo/U15}$BCAR-NKT cells. (D) Diagram showing the generation of healthy donor PBMC-derived M1-Mφs. (E) FACS detection of CD1d on the indicated cells. (F) Experimental design to study M1-Mφ targeting by $^{Allo/U15}$BCAR-NKT cells using an *in vitro* Mφ targeting assay. Mφ:NKT ratio = 1:1. (G) Mφ killing data at 24 h (n = 4).

(H-L) Studying the toxicity of $^{Allo/U15}$BCAR-NKT cells against human HSCs using primary MM patient BM samples. (H) Diagram showing the BM collection from primary MM patients. (I) FACS detection of long-term HSCs (LT-HSCs; gated as $CD34^+CD90^+CD45RA^-$ cells), short-term HSCs (ST-HSCs; gated as $CD34^+CD90^-CD45RA^-$ cells), and multi-potent progenitor cells (MPPs; gated as $CD34^+CD90^-CD45RA^+$ cells) in the MM patient BM cells. (J) FACS plots showing the lack of CD1d expression on primary MM patient BM HSCs. (K) Experimental design to study HSC killing

by $^{Allo/U15}$BCAR-NKT cells. MM patient BM cells were mixed with $^{Allo/U15}$BCAR-NKT cells at 1:1 ratio and cultured *in vitro*. (L) HSC killing data at 24 h (n = 4).
Representative of 3 experiments. Data are presented as the mean ± SEM. ns, not significant, *p < 0.05, **p < 0.01, ***p < 0.001, ****p < 0.0001, by 1-way ANOVA.

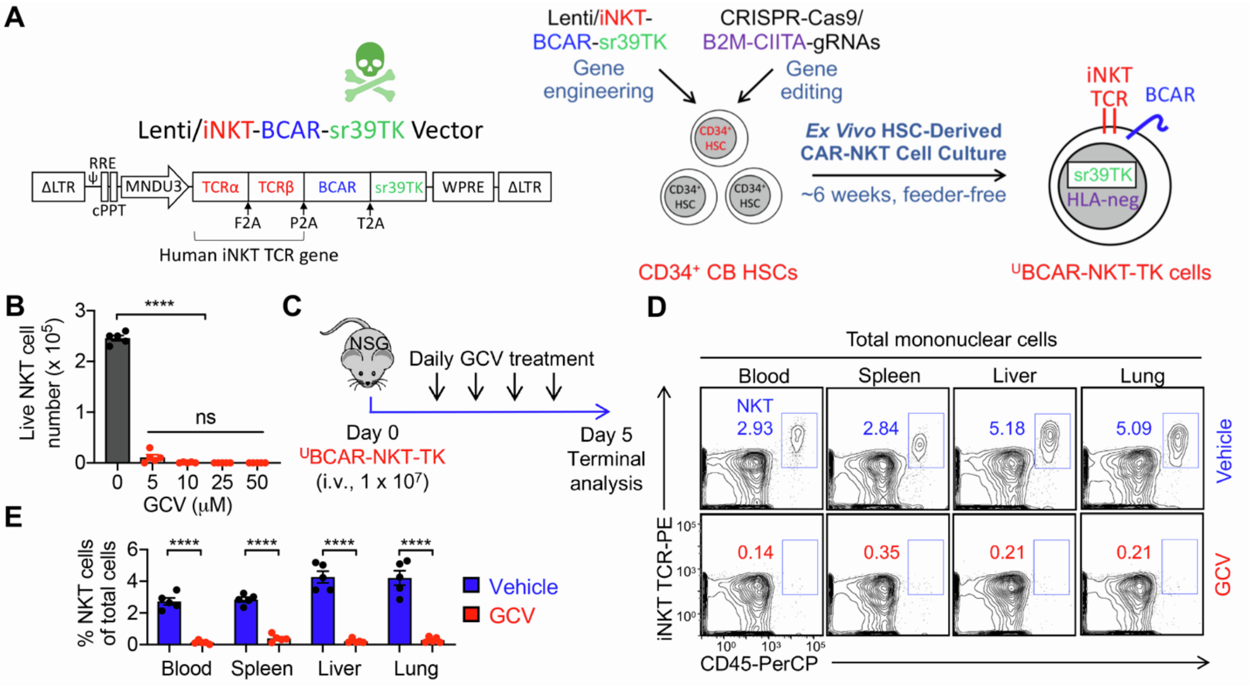

Figure S9. Incorporation of an sr39TK suicide switch in HSC-derived BCAR-NKT cells, related to Figure 8.

(A) Schematic showing the experimental design to generate HSC-engineered HLA-ablated BCAR-NKT cells equipped with an sr39TK suicide gene (denoted as ^{U}BCAR-NKT-TK cells). Lenti/iNKT-BCAR-sr39TK, lentivector encoding iNKT TCR, BCAR, and sr39TK.

(B) *In vitro* depletion of ^{U}BCAR-NKT-TK cells via GCV treatment (n = 4). GCV, ganciclovir that specifically depleting cells expressing the sr39TK gene.

(C-E) *In vivo* depletion of ^{U}BCAR-NKT-TK cells via GCV administration. (C) Experimental design.

(D) FACS detection of ^{U}BCAR-NKT-TK cells in the indicated tissues of the experimental animals.

(E) Quantification of (D).

Representative of 3 experiments. Data are presented as the mean ± SEM. ns, not significant; ****p < 0.0001 by Student's *t* test (E), or one-way ANOVA (B).

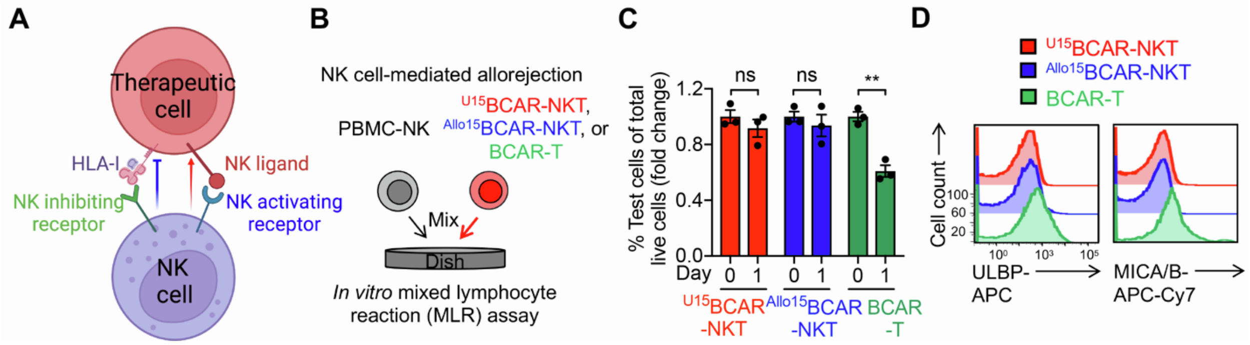

Figure S10. ^{U15}BCAR-NKT cells resist to NK cell-mediated allorejection.

(A) Diagram showing that host NK cells mediate allorejection through a double-trigger mechanism: 1) "missing self," which refers to the absence of matching HLA-I molecules on allogeneic cells, triggers the release of inhibitory signals mediated by NK inhibitory receptors, and 2) "stress signals," which refer to the upregulation of stress molecules on allogeneic cells, activate NK activating receptors such as NKG2D, DNAM-1, and NKp44.

(B-D) Studying the NK cell-mediated allorejection against ^{U15}BCAR-NKT cells using an *in vitro* MLR assay. PBMC-NK cells isolated from over ten random mismatched healthy donors were used. Data from one representative donor are presented. ^{Allo15}BCAR-NKT and BCAR-T cells were included as allorejection controls. (B) Experimental design. (C) FACS quantification of the indicated live cells on day 0 and day 1 (n = 3). (D) FACS measurements of NK ligands on the indicated therapeutic cells.

Representative of 3 experiments. Data are presented as the mean ± SEM. ns, not significant, **p < 0.01, by Student's *t* test.

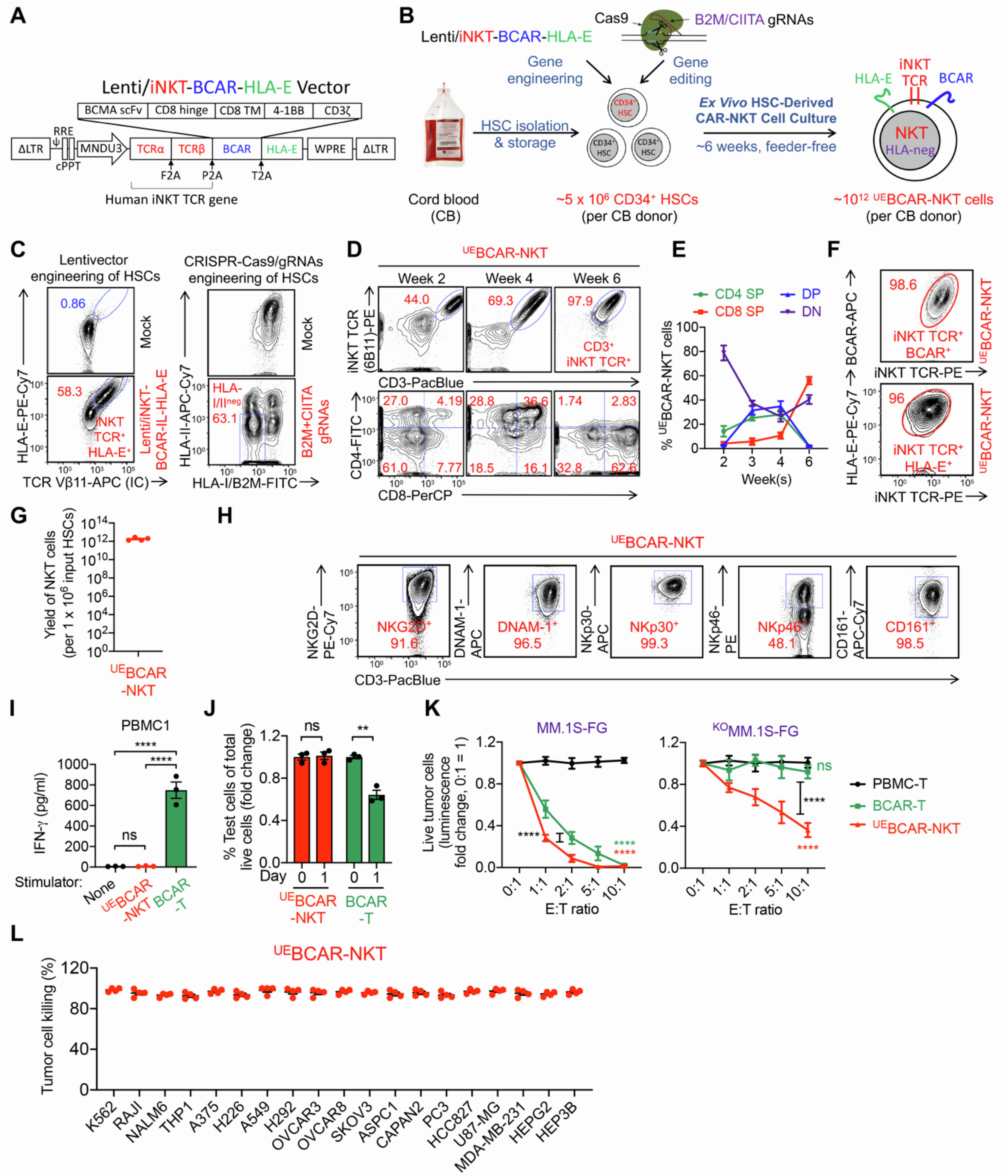

Figure S11. Generation of universal BCAR-NKT cells engineered with HLA-E to further ensure resistance to NK cell-mediated allorejection.

(A) Schematics showing the design of Lenti/iNKT-BCAR-HLA-E lentivector.

(B) Schematic showing the experimental design to generate HSC-engineered HLA-ablated BCAR-NKT cells equipped with an HLA-E gene (denoted as ^{UE}BCAR-NKT cells).

(C) Intracellular expression of iNKT TCR, surface expression of HLA-E, and surface ablation of HLA-I/II in CB HSCs 72 h after lentivector transduction and 48 h after CRISPR-Cas9 gene editing.

(D) FACS monitoring of the generation of ^{UE}BCAR-NKT cells.

(E) Quantification of the transition among four subpopulations of ^{UE}BCAR-NKT cells during their developmental stages.
(F) FACS detection of BCAR and HLA-E expressions on ^{UE}BCAR-NKT cells.
(G) Yield of ^{UE}BCAR-NKT cells (n = 4; n indicates different CB donors).
(H) FACS analyses of surface NK receptors on ^{UE}BCAR-NKT cells.
(I-J) Studying the T and NK cell-mediated allorejection against ^{UE}BCAR-NKT cells using *in vitro* MLR assays. BCAR-T cells were included as a control. (I) ELISA analyses of IFN-γ production on day 4 in the T cell-mediated allorejection assay (n = 3). (J) FACS quantification of the indicated live cells on day 0 and day 1 in the NK cell-mediated allorejection assay (n = 3).
(K-L) Tumor cell killing data by ^{UE}BCAR-NKT cells at 24 h (n = 4).
Representative of 3 experiments. Data are presented as the mean ± SEM. ns, not significant, *p < 0.05, **p < 0.01, ***p < 0.001, ****p < 0.0001, by Student's *t* test (J), 1-way ANOVA (I), or 2-way ANOVA (K).

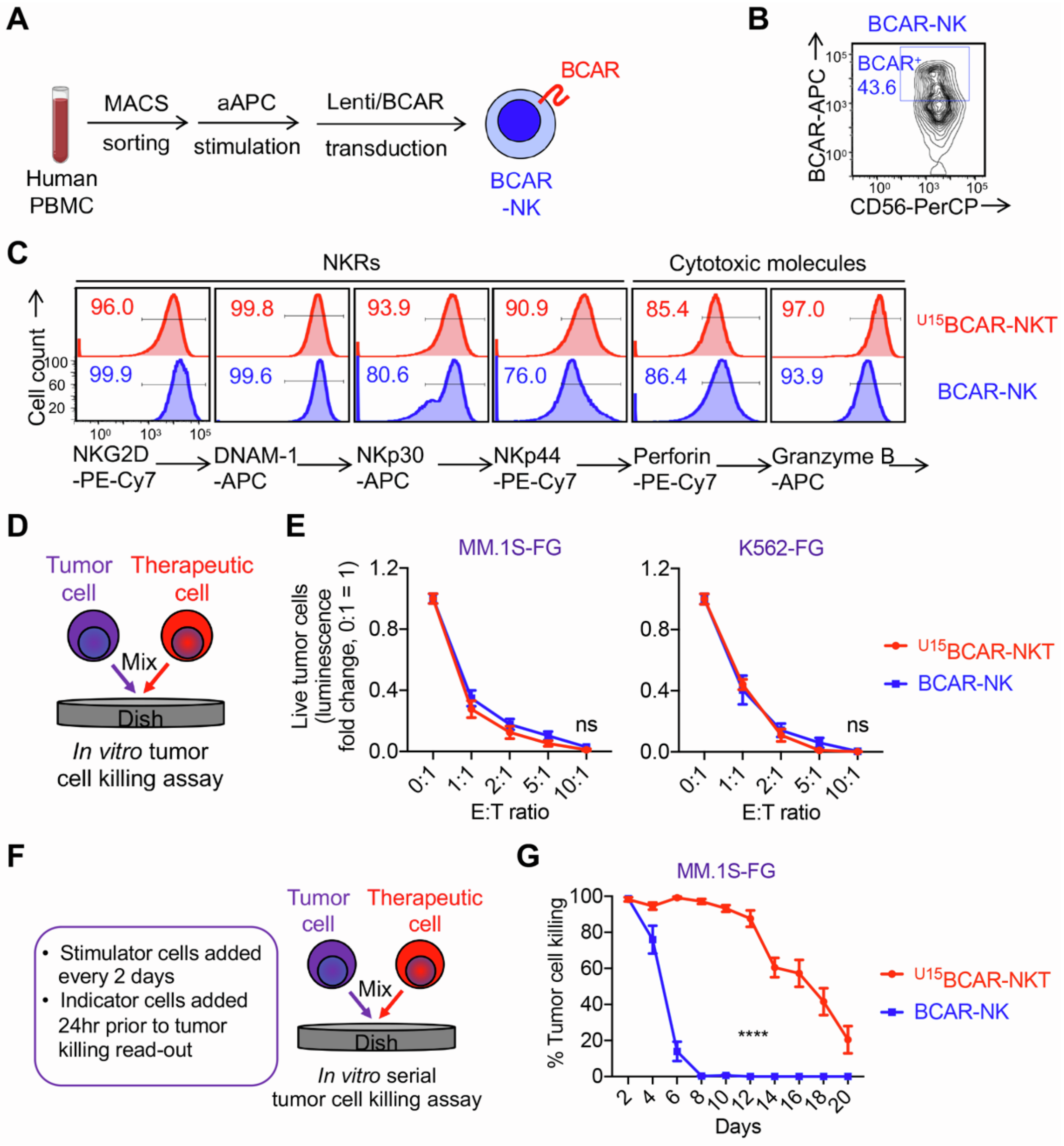

Figure S12. Comparison between ^{U15}BCAR-NKT cells and BCAR-engineered NK (BCAR-NK) cells.
(A) Schematics showing the generation of BCAR-NK cells.
(B) FACS plots showing the BCAR expression on BCAR-NK cells.
(C) FACS plots showing the surface NKR expression and intracellular cytotoxic molecule production of ^{U15}BCAR-NKT and BCAR-NK cells.
(D-E) Studying the *in vitro* antitumor efficacy of ^{U15}BCAR-NKT and BCAR-NK cells. (D) Experimental design. (E) Tumor cell killing data at 24 h (n = 4).
(F-G) Studying the long-term antitumor efficacy of ^{U15}BCAR-NKT and BCAR-NK cells using an *in vitro* serial tumor cell killing assay. (F) Experimental design. (G) Tumor cell killing data (n = 4).
Representative of two experiments. Data are presented as the mean ± SEM. ns, not significant, ****$p < 0.0001$, by two-way ANOVA.

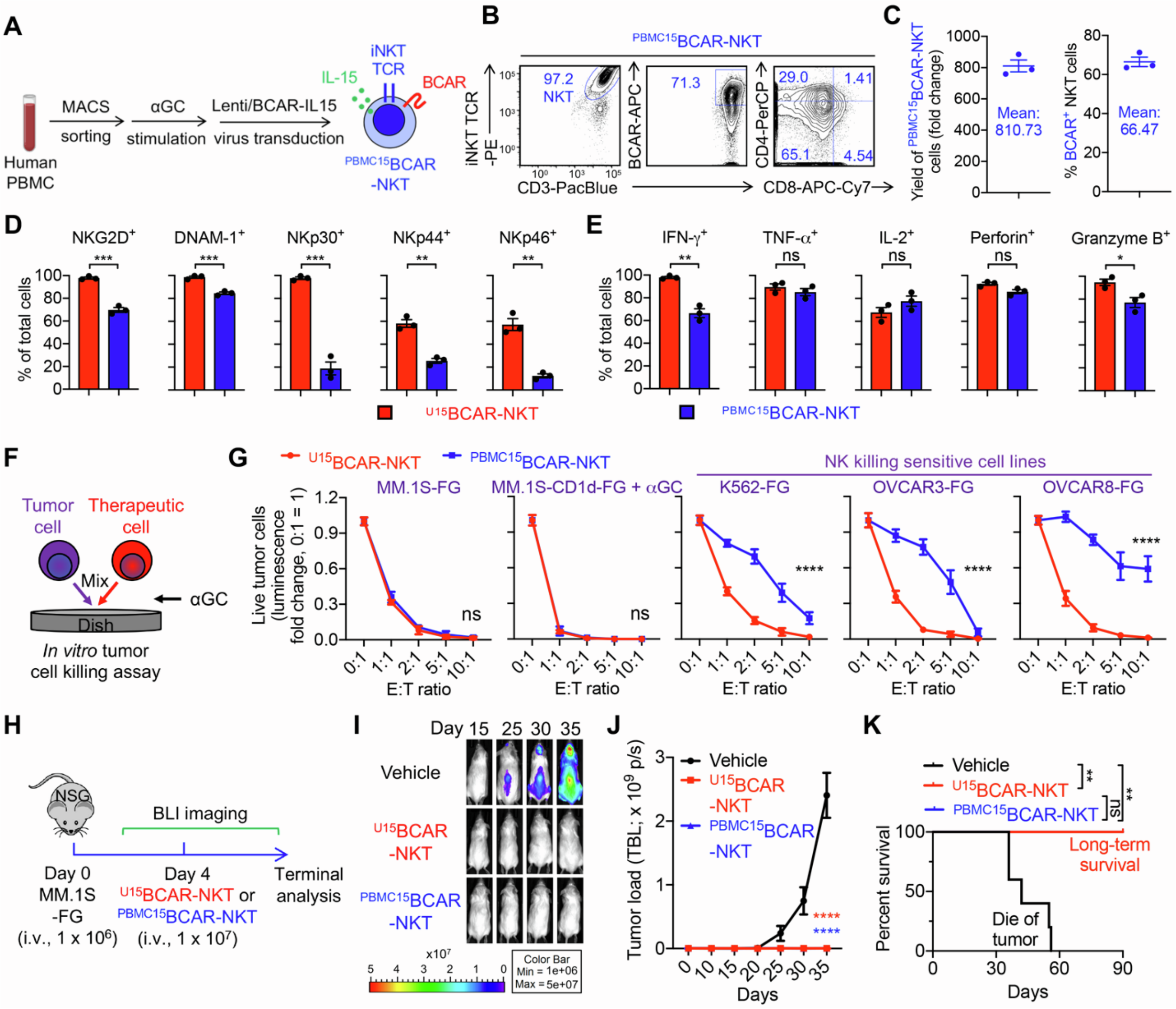

Figure S13. Comparison between ^{U15}BCAR-NKT cells and PBMC-derived IL-15-enhanced BCAR-engineered NKT (^{PBMC15}BCAR-NKT) cells.

(A) Schematics showing the generation of ^{PBMC15}BCAR-NKT cells.

(B) FACS plots showing the purity, CAR expression, and CD4/CD8 co-receptor expression of ^{PBMC15}BCAR-NKT cells.

(C) Yield and CAR expression of ^{PBMC15}BCAR-NKT cells (n = 3; n indicates different donors).

(D) FACS analyses of surface NKR expressions on ^{U15}BCAR-NKT and ^{PBMC15}BCAR-NKT cells (n = 3).

(E) FACS analyses of intracellular cytokine and cytotoxic molecule production in ^{U15}BCAR-NKT and ^{PBMC15}BCAR-NKT cells (n = 3).

(F-G) Studying the *in vitro* antitumor efficacy of ^{U15}BCAR-NKT and ^{PBMC15}BCAR-NKT cells. (F) Experimental design. (G) Tumor cell killing data at 24 h (n = 4).

(H-K) Studying the *in vivo* antitumor efficacy of ^{U15}BCAR-NKT and ^{PBMC15}BCAR-NKT cells in a human MM xenograft NSG mouse model. (H) Experimental design. (I) BLI images showing the presence of tumor cells in experimental mice over time. (J) Quantification of (I) (n = 5). (K) Kaplan-Meier survival curves of experimental mice over time (n = 5).

Representative of three (A-G) and two (H-K) experiments. Data are presented as the mean ± SEM. ns, not significant; *p < 0.05; **p < 0.01; ***p < 0.001; ****p < 0.0001, by one-way ANOVA

(D and E), two-way ANOVA (G and J), or by log rank (Mantel-Cox) test adjusted for multiple comparisons (K).

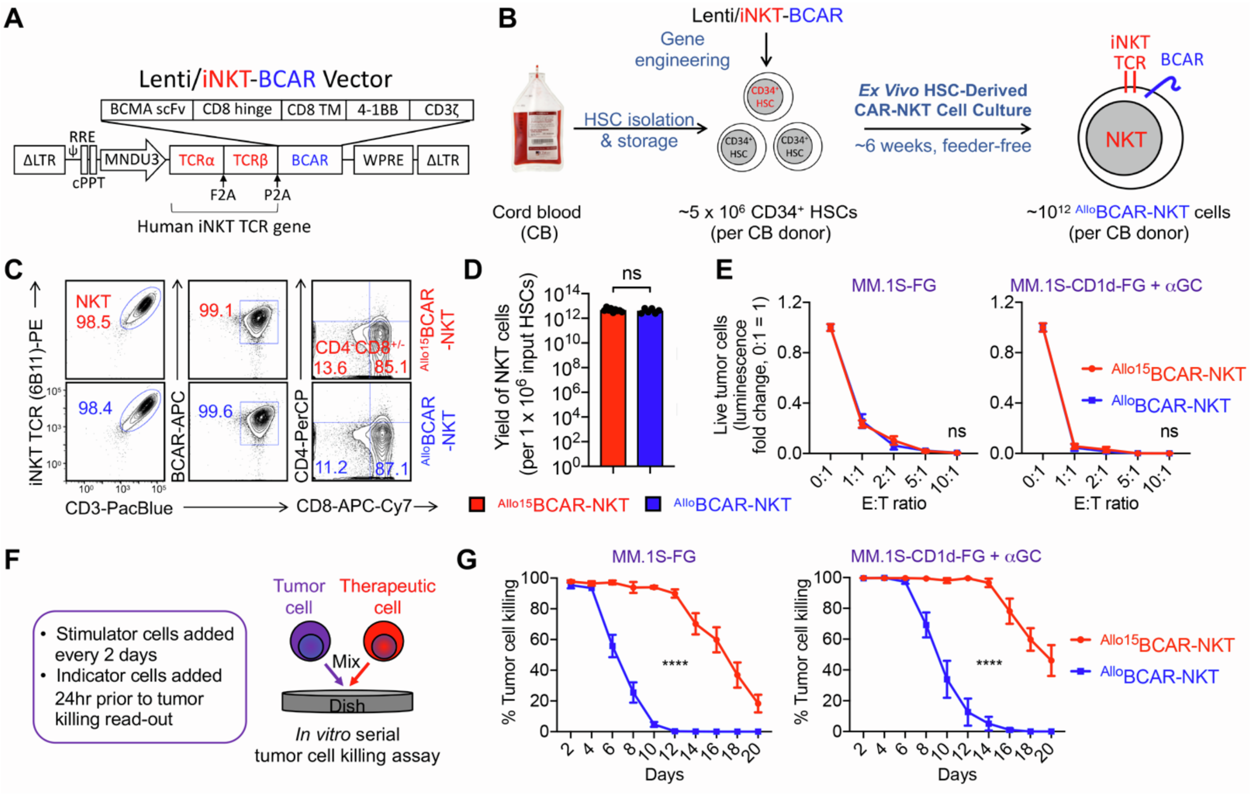

Figure S14. Comparison between non-IL-15-enhanced allogeneic BCAR-NKT (^{Allo}BCAR-NKT) cells with IL-15-enhanced ^{Allo15}BCAR-NKT cells.
(A) Schematics showing the design of Lenti/iNKT-BCAR lentivector.
(B) Schematic showing the experimental design to generate ^{Allo}BCAR-NKT cells.
(C) FACS detection of surface markers on $^{Allo/15}$BCAR-NKT cells.
(D) Yield of $^{Allo/15}$BCAR-NKT cells (n = 6-9; n indicates different donors).
(E) Tumor cell killing data by $^{Allo/15}$BCAR-NKT cells at 24 h (n = 4).
(F-G) Studying the long-term antitumor efficacy of $^{Allo/15}$BCAR-NKT cells using an *in vitro* serial tumor cell killing assay. (F) Experimental design. (G) Tumor cell killing data (n = 4).
Representative of 3 experiments. Data are presented as the mean ± SEM. ns, not significant, ****$p < 0.0001$, by Student's *t* test (D), or 2-way ANOVA (E and G).
