# Supplementary material for: Engineering allorejection-resistant CAR-NKT cells from hematopoietic stem cells for off-the-shelf cancer immunotherapy
Source: Mol Ther. 2024 Apr 6;32(6):1849–74. doi: 10.1016/j.ymthe.2024.04.005 (PMC11184334; doi:10.1016/j.ymthe.2024.04.005)
Supplement: Document S2. Article plus supplemental information [file mmc2.pdf]

Molecular Therapy
Original Article

Engineering allorejection-resistant CAR-NKT cells from hematopoietic stem cells for off-the-shelf cancer immunotherapy

Yan-Ruide Li,[1] Yang Zhou,[1] Jiaji Yu,[1] Yichen Zhu,[1] Derek Lee,[1] Enbo Zhu,[1,2] Zhe Li,[1] Yu Jeong Kim,[1] Kuangyi Zhou,[1] Ying Fang,[1] Zibai Lyu,[1] Yuning Chen,[1] Yanxin Tian,[1] Jie Huang,[1] Xinjian Cen,[1] Tiffany Husman,[1] Jae Min Cho,[2] Tzung Hsiai,[2] Jin J. Zhou,[3] Pin Wang,[4] Benjamin R. Puliafito,[5] Sarah M. Larson,[6,7] and Lili Yang[1,7,8,9]

[1]Department of Microbiology, Immunology & Molecular Genetics, University of California, Los Angeles, Los Angeles, CA 90095, USA; [2]Division of Cardiology, Department of Medicine, David Geffen School of Medicine, University of California, Los Angeles, Los Angeles, CA 90095, USA; [3]Department of Biostatistics, Fielding School of Public Health, University of California, Los Angeles, Los Angeles, CA 90095, USA; [4]Department of Chemical Engineering and Materials Science, University of Southern California, Los Angeles, CA 90089, USA; [5]Department of Hematology and Oncology, University of California, Los Angeles, Los Angeles, CA 90095, USA; [6]Department of Internal Medicine, University of California, Los Angeles, Los Angeles, CA 90095, USA; [7]Jonsson Comprehensive Cancer Centre, University of California, Los Angeles, Los Angeles, CA 90095, USA; [8]Eli and Edythe Broad Centre of Regenerative Medicine and Stem Cell Research, University of California, Los Angeles, Los Angeles, CA 90095, USA; [9]Molecular Biology Institute, University of California, Los Angeles, Los Angeles, CA 90095, USA

The clinical potential of current FDA-approved chimeric antigen receptor (CAR)-engineered T (CAR-T) cell therapy is encumbered by its autologous nature, which presents notable challenges related to manufacturing complexities, heightened costs, and limitations in patient selection. Therefore, there is a growing demand for off-the-shelf universal cell therapies. In this study, we have generated universal CAR-engineered NKT (^{U}CAR-NKT) cells by integrating iNKT TCR engineering and HLA gene editing on hematopoietic stem cells (HSCs), along with an *ex vivo*, feeder-free HSC differentiation culture. The ^{U}CAR-NKT cells are produced with high yield, purity, and robustness, and they display a stable HLA-ablated phenotype that enables resistance to host cell-mediated allorejection. These ^{U}CAR-NKT cells exhibit potent antitumor efficacy to blood cancers and solid tumors, both *in vitro* and *in vivo*, employing a multifaceted array of tumor-targeting mechanisms. These cells are further capable of altering the tumor microenvironment by selectively depleting immunosuppressive tumor-associated macrophages and myeloid-derived suppressor cells. In addition, ^{U}CAR-NKT cells demonstrate a favorable safety profile with low risks of graft-versus-host disease and cytokine release syndrome. Collectively, these preclinical studies underscore the feasibility and significant therapeutic potential of ^{U}CAR-NKT cell products and lay a foundation for their translational and clinical development.

INTRODUCTION

Autologous chimeric antigen receptor (CAR)-engineered T (CAR-T) cell therapy has demonstrated remarkable clinical responses in the treatment of hematological malignancies, particularly B cell malignancies and multiple myeloma (MM).[1–3] Despite its successes, current autologous CAR-T cell therapy faces significant challenges. It necessitates further enhancements in terms of efficacy and is inherently limited by its autologous nature.[4–7] Conventional αβ T cell-based cell products have the potential to induce graft-versus-host disease (GvHD) when introduced into allogeneic recipients.[8,9] Consequently, personalized CAR-T cells must be individually manufactured for each patient, rendering the therapy prohibitively costly and logistically complex, impeding its broad accessibility to all eligible cancer patients.[8,9] Therefore, there is a growing demand for allogeneic "off-the-shelf" cell products that can be manufactured on a large scale and readily distributed to address the needs of a diverse population of cancer patients.

Invariant natural killer T (NKT) cells, a distinct subset of unconventional αβ T cells, possess several distinctive attributes that render them exceptionally well-suited as cellular carriers for the development of allogeneic CAR-directed cell therapies in the context of cancer.[10–13] When compared with conventional CAR-T cells, CAR-engineered NKT (CAR-NKT) cells exhibit heightened efficacy in targeting tumor cells through multiple mechanisms. They demonstrate superior abilities in trafficking to and infiltrating tumor sites, modifying the immunosuppressive tumor microenvironment (TME), and, critically, do not incite GvHD.[14–16] However, the presence of NKT cells in human blood is extremely limited, typically ranging from 0.001% to 1% of total blood cells. This scarcity poses a formidable challenge in generating consistent and substantial quantities of allogeneic NKT cells suitable for CAR engineering.[17] Furthermore, allogeneic NKT

Received 31 December 2023; accepted 3 April 2024;
https://doi.org/10.1016/j.ymthe.2024.04.005.

Correspondence: Lili Yang, PhD, Department of Microbiology, Immunology & Molecular Genetics, University of California, Los Angeles, Los Angeles, CA 90095, USA.
E-mail: liliyang@ucla.edu

This is an open access article under the CC BY license (http://creativecommons.org/licenses/by/4.0/).

cell products may be subject to rejection by host immune cells, leading to restricted persistence and compromised antitumor efficacy.[18] Consequently, there is a pressing need to explore alternative sources for the production of CAR-NKT cells and develop strategies to render these cells resistant to allorejection.

Hematopoietic stem cells (HSCs) hold the promise for genetic engineering and subsequent differentiation into a variety of immune cell types, including NKT cells.[5,19,20] Previously, we successfully generated HSC-engineered NKT cells through the utilization of an artificial thymic organoid culture method.[21] However, this method presented inherent challenges associated with scalability and manufacturing due to the reliance on organoids and feeder cells of murine origin. Here, we present an advanced technology for the differentiation of gene-engineered HSCs into CAR-NKT cells using a feeder-free culture system. In addition, the incorporation of potent gene-editing tools, such as the CRISPR-Cas9 system, enables the genetic modification of NKT cells to confer resistance against host immune cell-mediated depletion.[22] This includes the knockout of the beta 2-microglobulin (*B2M*) gene to eliminate HLA-I molecule expression on NKT cells, thus evading host $CD8^+$ T cell-mediated rejection,[23] as well as the knockout of the *CIITA* gene to abrogate HLA-II molecule expression on NKT cells, thereby preventing $CD4^+$ T cell-mediated rejection.[24]

In this study, we have harnessed a synergistic approach that integrates iNKT TCR gene engineering and CRISPR-Cas9 gene editing on HSCs, as well as a feeder-free HSC differentiation culture. This strategy has enabled the generation of a diverse spectrum of HLA-ablated universal CAR-NKT (^{U}CAR-NKT) cell products. We have conducted a comprehensive series of preclinical investigations on these ^{U}CAR-NKT cell products, which encompass assessments of their manufacturing, pharmacology, efficacy, mechanism of action (MOA), pharmacokinetics/pharmacodynamics (PK/PD), safety, and immunogenicity.

RESULTS

HSC-engineered HLA-ablated ^{U}CAR-NKT cells can be generated with high yield and purity

Cryopreserved human cord blood (CB)-derived $CD34^+$ hematopoietic stem and progenitor cells, hereafter referred to as HSCs, were procured from commercial sources such as HemaCare. Subsequently, these HSCs underwent a five-stage, 6-week, feeder-free process to yield two distinct cellular products: allogeneic IL-15-enhanced BCMA-targeting CAR (BCAR)-NKT cells (referred to as ^{Allo15}BCAR-NKT cells) and HLA-ablated universal IL-15-enhanced BCAR-NKT cells (referred to as ^{U15}BCAR-NKT cells) (Figures 1A and S1).

To produce ^{Allo15}BCAR-NKT cells, the HSCs were genetically engineered using a Lenti/iNKT-BCAR-IL-15 vector (Figures 1B and S2A). This lentivector encompassed a pair of iNKT TCR α and β chains, previously employed in the development of autologous and allogeneic HSC-engineered NKT cell therapies for cancer.[21,25] In addition, the lentivector included a BCAR, a clinical modality utilized for MM treatment,[26] and IL-15, a cytokine known to augment the longevity and *in vivo* antitumor efficacy of CAR-NKT cells.[13,15]

To produce ^{U15}BCAR-NKT cells, the HSCs were genetically engineered using the same lentivector. In addition, a CRISPR-Cas9/B2M-CIITA-gRNAs complex was simultaneously employed to ablate the expression of HLA-I/II molecules (Figures 1B and S2A). Notably, the gene-engineering and -editing processes exhibited high efficiency, consistently achieving over 50% lentivector transduction rate and a concurrent over 50% HLA-I/II double-ablation rate (Figures 1C, 1D, S2B, and S2C). The genetically engineered HSCs were then cultured for a duration of 48 h in a classical X-VIVO 15-based HSC medium.

Gene-engineered HSCs were then subjected to a 6-week feeder-free culture process, resulting in the generation of a designated $^{Allo/U15}$BCAR-NKT cell product: stage 1 HSC expansion (∼2 weeks), stage 2 NKT differentiation (∼1 week), stage 3 NKT deep differentiation (∼1 week), and stage 4 NKT expansion (∼2 weeks) (Figures S1 and S2D). The entirety of this five-stage culture protocol was designed to be executed in a feeder-free and serum-free manner, thereby providing significant support for both clinical and commercial development.[27] Alternatively, two feeder-dependent strategies were available for stage 4 NKT expansion (Figure S1). These strategies included the utilization of either α-galactosylceramide (αGC)-loaded healthy donor peripheral blood mononuclear cells (PBMCs) or K562-based artificial antigen-presenting cells (aAPCs). Both feeder-dependent approaches were found to be suitable for clinical and commercial development.[15,27] Notably, the $^{Allo/U15}$BCAR-NKT cells generated through the three different expansion approaches exhibited comparable expansion folds, immunophenotypes, and functionalities, albeit with a slightly higher expansion fold observed using the aAPC approach (Figures S2E and S2F).

The differentiation process of $^{Allo/U15}$BCAR-NKT cells followed a conventional developmental trajectory observed in NKT cells,[28] transitioning from a $CD4^-CD8^-$ double-negative (DN) stage to a $CD4^+CD8^+$ double-positive (DP) stage. Subsequently, these cells further matured into either a $CD8^+$ single-positive (SP) stage or retained the DN phenotype (Figures 1E and 1F). Notably, the final $^{Allo/U15}$BCAR-NKT cell product did not include a $CD4^+$ SP subpopulation, a component typically present in endogenous human NKT cells (Figure 1E).[28–30] In general, CD8 SP/DN human NKT cells are recognized for their pro-inflammatory characteristics and heightened cytotoxicity, rendering them particularly appealing for applications in cancer immunotherapy.[28–30]

The co-delivery of all therapeutic genes (i.e., iNKT TCR, BCAR, and IL-15) within the same lentivector resulted in all the end product $^{Allo/U15}$BCAR-NKT cells expressing both BCAR and IL-15 (Figure 1G). This characteristic rendered these cell products inherently pure and "clonal," obviating the need for any further enrichment or purification steps. It is important to note that the introduction of

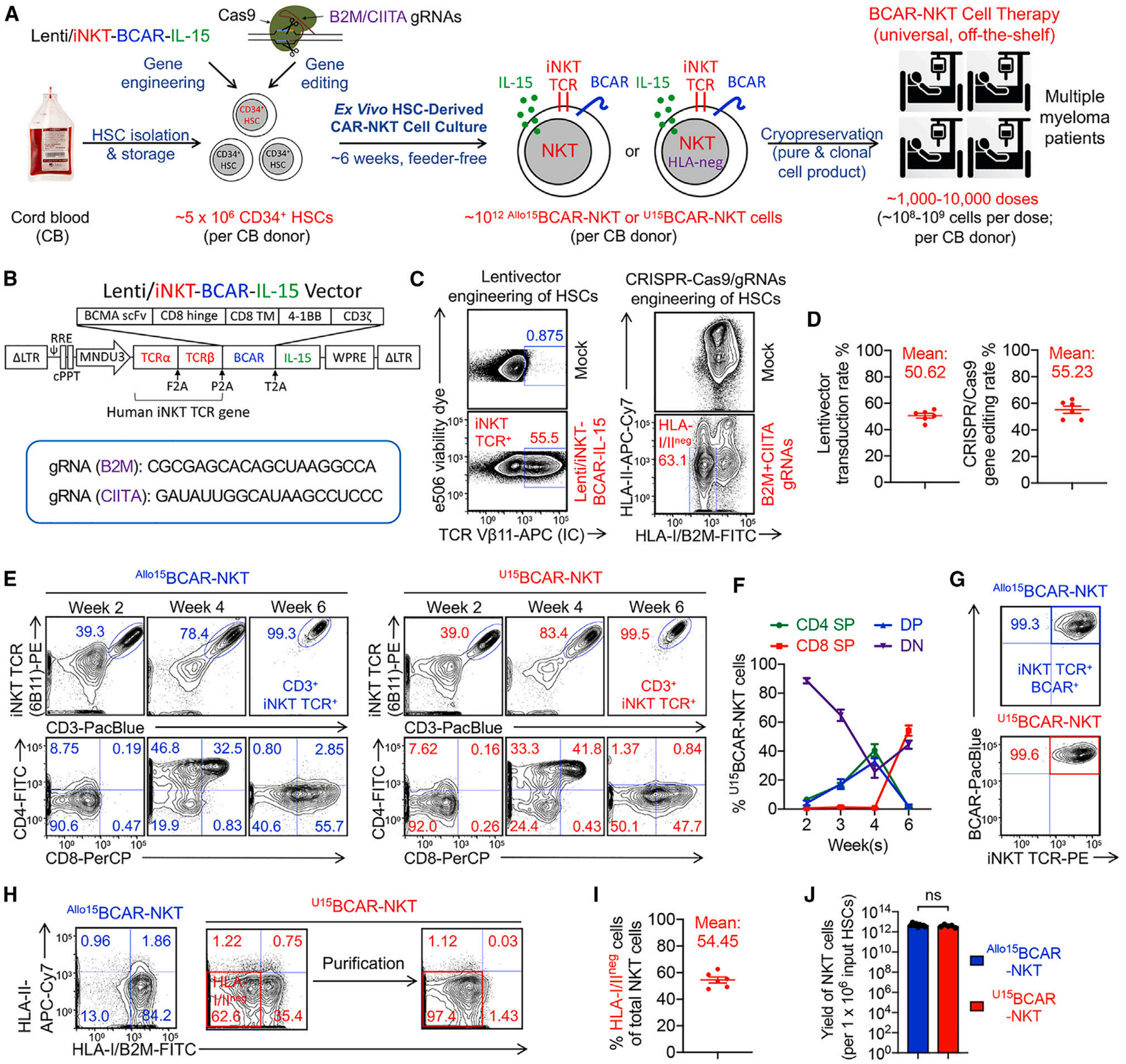

Figure 1. HSC-derived BCMA-targeting CAR-engineered NKT (BCAR-NKT) cells with/out HLA gene editing can be produced at high yield and purity

(A) Schematics showing the generation of two BCAR-NKT cell products: allogeneic IL-15-enhanced BCAR-NKT (^{Allo15}BCAR-NKT) cells, and HLA-ablated universal IL-15-enhanced BCAR-NKT (^{U15}BCAR-NKT) cells. HSC, hematopoietic stem cell; CAR, chimeric antigen receptor; gRNA, guide RNA; HLA-neg, HLA negative. (B) Schematics showing the design of Lenti/iNKT-BCAR-IL-15 lentivector and gRNA sequences of B2M and CIITA. BCAR, B cell maturation antigen (BCMA)-targeting CAR; ΔLTR, self-inactivating long-term repeats; MNDU3, internal promoter derived from the MND retroviral LTR U3 region; φ, packaging signal with the splicing donor and splicing acceptor sites; RRE, rev-responsive element; cPPT, central polypurine tract; WPRE, woodchuck responsive element. (C) Intracellular expression of iNKT TCR (identified as Vβ11$^+$) and surface ablation of HLA-I/II (identified as HLA-I/B2M$^-$HLA-II$^-$) in CB HSCs 72 h after lentivector transduction and 48 h after CRISPR-Cas9 gene editing. (D) Quantification of Lenti/iNKT-BCAR-IL-15 lentivector transduction rate and CRISPR-Cas9 gene editing rate (n = 6). (E) FACS monitoring of the generation of $^{Allo/U15}$BCAR-NKT cells. iNKT TCR was stained using a 6B11 monoclonal antibody. (F) Quantification of the transition among four subpopulations of ^{U15}BCAR-NKT cells during their developmental stages. CD4 SP, CD4 single-positive; CD8 SP, CD8 single-positive; DP, CD4 CD8 double-positive; DN, CD4 CD8 double-negative. (G) FACS detection of BCAR expression on $^{Allo/U15}$BCAR-NKT cells. BCAR was stained using an anti-mouse IgG F(ab')2 antibody. (H) FACS detection of HLA-I/II expression on $^{Allo/U15}$BCAR-NKT cells. HLA-I/II-negative ^{U15}BCAR-NKT cells were purified using MACS or FACS sorting. (I) Quantification of HLA-I/II-negative cells among unpurified ^{U15}BCAR-NKT cells (n = 6). (J) Yield of $^{Allo/U15}$BCAR-NKT cells (n = 5–9; n indicates different donors). Representative of 1 (A, B, and J) and >10 (C–I) experiments. Data are presented as the mean ± SEM. ns, not significant, by Student's t test (J).

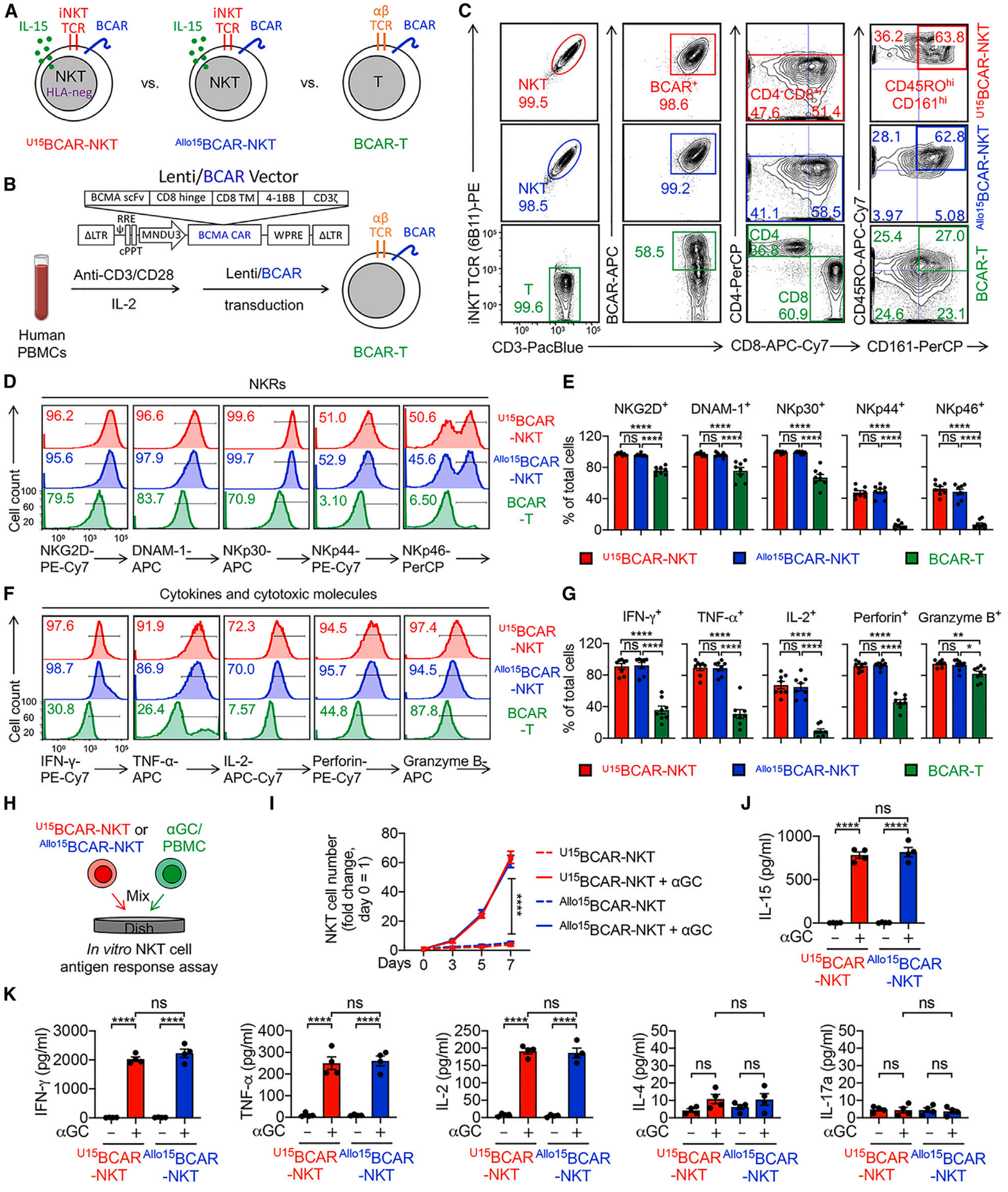

Figure 2. $^{Allo/U15}$BCAR-NKT cells display a typical NKT cell phenotype and a Th0/Th1-prone, highly cytotoxic functionality

(A) Diagram of ^{Allo15}BCAR-NKT cells, ^{U15}BCAR-NKT cells, and healthy donor PBMC-derived conventional T cells engineered with the same BCAR (denoted as BCAR-T cells). (B) Diagram showing the generation of BCAR-T cells from healthy donor PBMCs. (C) FACS detection of surface markers on $^{Allo/U15}$BCAR-NKT cells. BCAR-T cells were included as a control. (D and E) FACS detection (D) and quantification (E) of NK receptors (NKRs) expression on the indicated cells (n = 8). (F–G) FACS detection (F) and

(legend continued on next page)

CRISPR-Cas gene editing did not disrupt the differentiation of CAR-NKT cells, and there was no discernible selection between HLA-I/II-ablated cells and non-edited cells throughout the entire culture process (Figures 1E, 1G, and S2G). The approach yielded CAR-NKT cell products with a double-ablation rate exceeding 50% for HLA-I/II, which could be further enriched through either MACS or fluorescence-activated cell sorting (FACS) sorting (Figures 1H, 1I, and S2G). Interestingly, ^{Allo15}BCAR-NKT cells already expressed low levels of HLA-I molecules and nearly no HLA-II molecules, suggesting that these cells may inherently be well-suited for allogeneic transfer (Figure 1H).[18,31]

The resultant cell products comprised of pure and clonal $^{Allo/U15}$BCAR-NKT cells, devoid of bystander conventional αβ T cells and risk of GvHD (Figure 1E). This manufacturing process demonstrated robustness, yielding high quantities of pure $^{Allo/U15}$BCAR-NKT cells across over 10 CB donors tested. Based on estimations, from a single CB donor containing approximately 5×10^6 $CD34^+$ HSCs, it is projected that approximately 10^{12} $^{Allo/U15}$BCAR-NKT cells could be generated (Figures 1J and S2D). This would allow for potential formulation into 1,000–10,000 doses, with each dose containing approximately 10^8–10^9 cells, in accordance with approved CAR-T cell therapy dosing standards (Figure 1A).[32]

^{U}CAR-NKT cells display a typical NKT cell phenotype and a Th0/Th1-prone, highly cytotoxic functionality

The phenotype and functionality of $^{Allo/U15}$BCAR-NKT cells were analyzed in comparison with BCAR-engineered conventional αβ T (BCAR-T) cells derived from endogenous human PBMCs (Figures 2A and 2B). Flow cytometry analysis demonstrated that $^{Allo/U15}$BCAR-NKT cells exhibited a typical NKT cell phenotype (Figure 2C). In contrast to conventional BCAR-T cells, $^{Allo/U15}$BCAR-NKT cells expressed high levels of NK receptors (NKRs) (e.g., CD161, NKG2D, and DNAM-1) and produced exceedingly high levels of effector cytokines (e.g., IFN-γ, TNF-α, and IL-2) and cytotoxic molecules (e.g., Perforin and Granzyme B) (Figures 2D–2G). These characteristics, in line with their CD8 SP/DN phenotype, make $^{Allo/U15}$BCAR-NKT cells particularly well-suited for cancer therapy applications.[33,34]

To assess the functionality of the iNKT TCR, $^{Allo/U15}$BCAR-NKT cells were stimulated with the agonist glycolipid antigen αGC (Figure 2H).[35,36] These cells exhibited robust proliferation (Figure 2I) and secreted elevated levels of IL-15 and Th0/Th1 cytokines (i.e., IFN-γ, TNF-α, and IL-2), while producing lower levels of Th2/Th17 cytokines (i.e., IL-4 and IL-17a) (Figures 2J and 2K). These features indicated a Th0/Th1-prone functionality of $^{Allo/U15}$BCAR-NKT cells, consistent with their CD8 SP/DN phenotype (Figures 1E and 2C).[28,29] Importantly, the gene-editing of HLA molecules did not disrupt either the development or the phenotype and functionality of ^{U15}BCAR-NKT cells (Figures 1 and 2).

^{U}CAR-NKT cells resist T cell-mediated allorejection

For allogeneic cell therapies, a critical concern is the risk of host cell-mediated allorejection, which can result in the depletion of allogeneic therapeutic cells by the host's immune system, particularly by host CD8 and CD4 T cells due to their recognition of disparate HLA-I/II molecules present on allogeneic therapeutic cells.[18,21,37,38] In comparison with BCAR-T cells and ^{Allo15}BCAR-NKT cells, ^{U15}BCAR-NKT cells exhibit a complete absence of surface HLA-I/II molecules (Figures 3A, 3B, S3A, and S3B). Intriguingly, even in the absence of HLA gene-editing, ^{Allo15}BCAR-NKT cells already manifest markedly reduced HLA-I expression and nearly undetectable HLA-II molecules (Figures 3A, 3B, S3A, and S3B). The low HLA-I/II expression feature of ^{Allo15}BCAR-NKT cells remained stable and was maintained throughout the entire 6-week cell culture (Figures S3C and S3D). This unique feature confers upon $^{Allo/U15}$BCAR-NKT cells a lower level of immunogenicity, potentially allowing these cells to persist within an allogeneic host for an extended duration, thereby delivering therapeutic benefits even without the necessity for gene editing to ablate surface HLA-I/II molecules. Indeed, in an *in vitro* mixed lymphocyte reaction (MLR) assay designed to study T cell-mediated alloresponse (Figure 3C), compared with conventional BCAR-T cells, ^{Allo15}BCAR-NKT cells elicited significantly diminished response from multiple donor-mismatched PBMCs (Figures 3D, 3E, and S3E). As expected, ^{U15}BCAR-NKT cells induced nearly imperceptible T cell-mediated alloresponse when co-cultured with these mismatched healthy donor PBMCs (Figures 3D, 3E, and S3E).

Subsequently, we assessed the susceptibility of $^{Allo/U15}$BCAR-NKT cells to T cell-mediated allorejection in a human xenograft NSG mouse model (Figure 3F). To mimic the presence of host T cells, donor-mismatched PBMCs were first injected into the mice. Following this, therapeutic cells labeled with firefly luciferase and enhanced green fluorescence protein dual reporters (FG) were introduced into the mice, and their PK/PD profiles and resistance to donor-mismatched T cells were evaluated through bioluminescence imaging (BLI) (Figures 3F–3H).

In the absence of donor-mismatched PBMC injection, $^{Allo/U15}$BCAR-NKT cells exhibited distinct PK/PD dynamics when compared with BCAR-T cells. $^{Allo/U15}$BCAR-NKT cells demonstrated robust expansion, reaching a peak at approximately 2–3 weeks, followed by a gradual decline, yet they could persist *in vivo* for over 2 months (Figures 4I and 4J). Conversely, BCAR-T cells initiated expansion 2 weeks post-injection, rapidly increased in number, and eventually

quantification (G) of intracellular cytokines and cytotoxic molecules production by the indicated cells (n = 8). (H–K) Studying the antigen responses of $^{Allo/U15}$BCAR-NKT cells. $^{Allo/U15}$BCAR-NKT cells were stimulated with/out αGC/PBMC for 1 week. (H) Experimental design. (I) Growth curve of $^{Allo/U15}$BCAR-NKT cells (n = 4). (J) ELISA analyses of IL-15 production by $^{Allo/U15}$BCAR-NKT cells cultured in the presence or absence of αGC stimulation for 48 h (n = 4). (K) ELISA analyses of effector cytokine (IFN-γ, TNF-α, IL-2, IL-4, and IL-17a) production on day 7 (n = 4). Representative of 3 experiments. Data are presented as the mean ± SEM. ns, not significant; *$p < 0.05$, **$p < 0.01$, ****$p < 0.0001$ by one-way ANOVA.

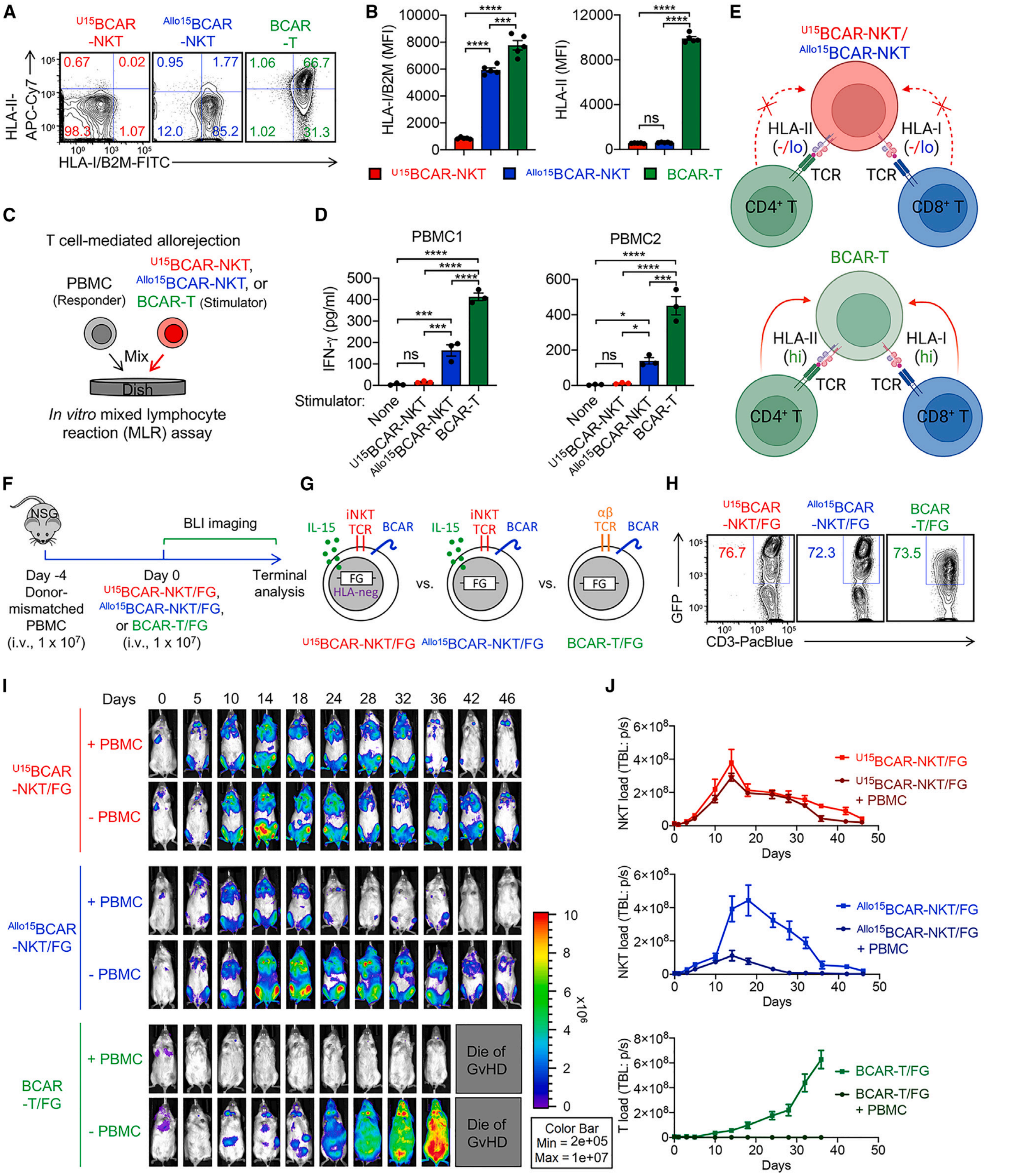

Figure 3. U15BCAR-NKT cells display an HLA-negative phenotype and resist to T cell-mediated allorejection

(A and B) Studying the HLA expression on Allo/U15BCAR-NKT cells. Conventional BCAR-T cells were included as a control. (A) FACS measurements of surface HLA-I/II on Allo/U15BCAR-NKT cells. (B) Quantification of (A) (n = 5). (C and D) Studying the T cell-mediated allorejection against Allo/U15BCAR-NKT cells using an *in vitro* mixed

(legend continued on next page)

led to the demise of mice due to GvHD (Figures 4I and 4J). In the presence of donor-mismatched PBMCs, ^{U15}BCAR-NKT cells displayed potent resistance, characterized by their PK/PD profiles and persistence being comparable with the condition without allogeneic T cells (Figures 4I and 4J). On the other hand, ^{Allo15}BCAR-NKT cells exhibited a degree of resistance to T cell-mediated allorejection, with limited expansion, followed by a rapid decline, ultimately disappearing 40 days after injection (Figures 4I and 4J). In stark contrast, conventional BCAR-T cells were vigorously rejected by allogeneic T cells, as they failed to expand at all in the experimental mice (Figures 4I and 4J).

Collectively, these studies unveiled a stable HLA-I/II-low phenotype of ^{Allo15}BCAR-NKT cells and an HLA-I/II-negative phenotype of ^{U15}BCAR-NKT cells. These unique characteristics bestow upon them a distinct advantage in resisting host T cell-mediated rejection when compared with other allogeneic cell products derived from healthy donor PBMCs. The observed "low immunogenicity" features of $^{Allo/U15}$BCAR-NKT cells provide a strong rationale for their potential application in off-the-shelf cell therapy strategies.

^{U}CAR-NKT cells directly kill tumor cells at high efficacy and using multiple targeting mechanisms

To study the tumor targeting efficacy and MOA of $^{Allo/U15}$BCAR-NKT cells, we utilized the BCMA^{+} human MM cell line, MM.1S, which was genetically modified to express FG for luciferase assay and flow cytometry monitoring (denoted as MM.1S-FG) (Figure 4A). It is worth noting that a significant proportion of primary MM tumor cells express both BCMA and CD1d, rendering them susceptible to both BCAR and iNKT TCR-mediated targeting, although CD1d expression levels may fluctuate with respect to MM disease stages.[21,39,40] Given that the parental MM.1S cell line lacks CD1d expression, we also overexpressed CD1d expression in the MM.1S-FG cell line to establish a BCMA^{+}CD1d^{+} MM.1S-CD1d-FG cell line for the investigation of iNKT TCR/BCAR dual targeting (Figures 4A and 4B). Furthermore, a BCMA^{-}CD1d^{-} ^{KO}MM.1S-FG cell line was generated by disrupting BCMA expression in MM.1S-FG cells through CRISPR-Cas9 gene editing (Figures 4A and 4B).

In an *in vitro* tumor cell killing assay, $^{Allo/U15}$BCAR-NKT cells demonstrated superior tumor cell killing efficacy to all three MM.1S tumor cell lines compared with conventional BCAR-T cells (Figures 4C and 4D). In contrast to BCAR-T cells, which solely relied on BCAR-BCMA recognition for tumor cell killing, $^{Allo/U15}$BCAR-NKT cells exhibited a reduced dependence on BCAR/BCMA recognition, although they still benefited from it (Figure 4D). Interestingly, $^{Allo/U15}$BCAR-NKT cells exhibited enhanced tumor cell killing in the presence of CD1d/αGC, indicating an iNKT TCR-directed targeting mechanism (Figure 4D). Moreover, $^{Allo/U15}$BCAR-NKT cells effectively eliminated BCMA^{-}CD1d^{-} tumor cells through recognition by NKRs such as NKG2D and DNAM-1, confirming an NKR-mediated targeting mechanism (Figures 4D–4F). Notably, even when compared with PBMC-derived NK (PBMC-NK) cells, $^{Allo/U15}$BCAR-NKT cells displayed a heightened tumor cell killing capacity targeting over 20 different tumor cell lines *in vitro* (Figures S4A and S4B). This enhanced performance can be attributed to their inherent capacity for NKR-mediated tumor cell killing (Figures 2D, 2E, and S4C–S4F). Furthermore, these cells demonstrated potent tumor suppression in an OVCAR8-FG human ovarian cancer xenograft mouse model *in vivo* through their intrinsic NK killing (Figures S5A–S5D). In summary, $^{Allo/U15}$BCAR-NKT cells utilize CAR/TCR/NKR triple-targeting mechanisms to target tumor cells (Figure 4G). These multiple targeting mechanisms may enhance their capacity to overcome tumor immune evasion during immunotherapy.[2,41,42]

The direct recognition and targeting of MM.1S tumor cells by $^{Allo/U15}$BCAR-NKT cells were observable through scanning electron microscopy (SEM) (Figure S6A). Aligned with their diverse targeting mechanisms and robust cytotoxicity, $^{Allo/U15}$BCAR-NKT cells exhibited heightened expression of activation markers (i.e., CD69), and increased production of effector cytokines (i.e., IFN-γ) and cytotoxic molecules (i.e., Perforin and Granzyme B), compared with BCAR-T cells following co-culture with tumor cells (Figures 4H–4J and S6B). Notably, $^{Allo/U15}$BCAR-NKT cells generated through three expansion approaches (i.e., αCD3/αCD28 Ab, αGC/PBMCs, and aAPCs) exhibited comparable antitumor capacities (Figure S6C), indicating the viability of utilizing all three expansion methods to produce functional therapeutic cell products to treat cancers.

Furthermore, a cohort of primary MM patient bone marrow (BM) samples was collected and employed to assess the tumor cell killing efficacy of $^{Allo/U15}$BCAR-NKT cells (Figure 4K). The co-expression of BCMA, CD1d, and NK ligands was identified on MM tumor cells across all the samples (Figure 4L). In an *in vitro* tumor cell killing assay, $^{Allo/U15}$BCAR-NKT cells demonstrated a superior effectiveness in eliminating primary MM tumor cells compared with conventional BCAR-T cells (Figures 4M, 4N, and S6D). Collectively, these results underscore the compelling potential of $^{Allo/U15}$BCAR-NKT cells for off-the-shelf cancer immunotherapy. The high antitumor efficacy and multiple tumor-targeting mechanisms of $^{Allo/U15}$BCAR-NKT

lymphocyte reaction (MLR) assay. PBMCs from over 10 random mismatched healthy donors were used as responder cells, and irradiated $^{Allo/U15}$BCAR-NKT cells were used as stimulator cells. Data from four representative donors are presented. BCAR-T cells were included as an allorejection control. (C) Experimental design. (D) ELISA analyses of IFN-γ production on day 4 (n = 3). (E) Diagram showing $^{Allo/U15}$BCAR-NKT cells display HLA-low/negative phenotype and resist T cell-mediated allorejection. (F–J) Studying the T cell-mediated allorejection against $^{Allo/U15}$BCAR-NKT cells using an *in vivo* humanized NSG mouse model. (F) Experimental design. BLI, bioluminescence live animal imaging. (G) Diagram of ^{Allo15}BCAR-NKT/FG, ^{U15}BCAR-NKT/FG, and BCAR-T/FG cells. The three therapeutic cells were engineered to overexpress the firefly luciferase and green fluorescence protein (FG) dual reporters. (H) FACS detection of FG expression in the indicated cells. (I) BLI images showing the presence of therapeutic cells in experimental mice over time. (J) Quantification of (I) (n = 3). Representative of 3 experiments. Data are presented as the mean ± SEM. ns, not significant; *p < 0.05, **p < 0.01, ****p < 0.0001 by one-way ANOVA.

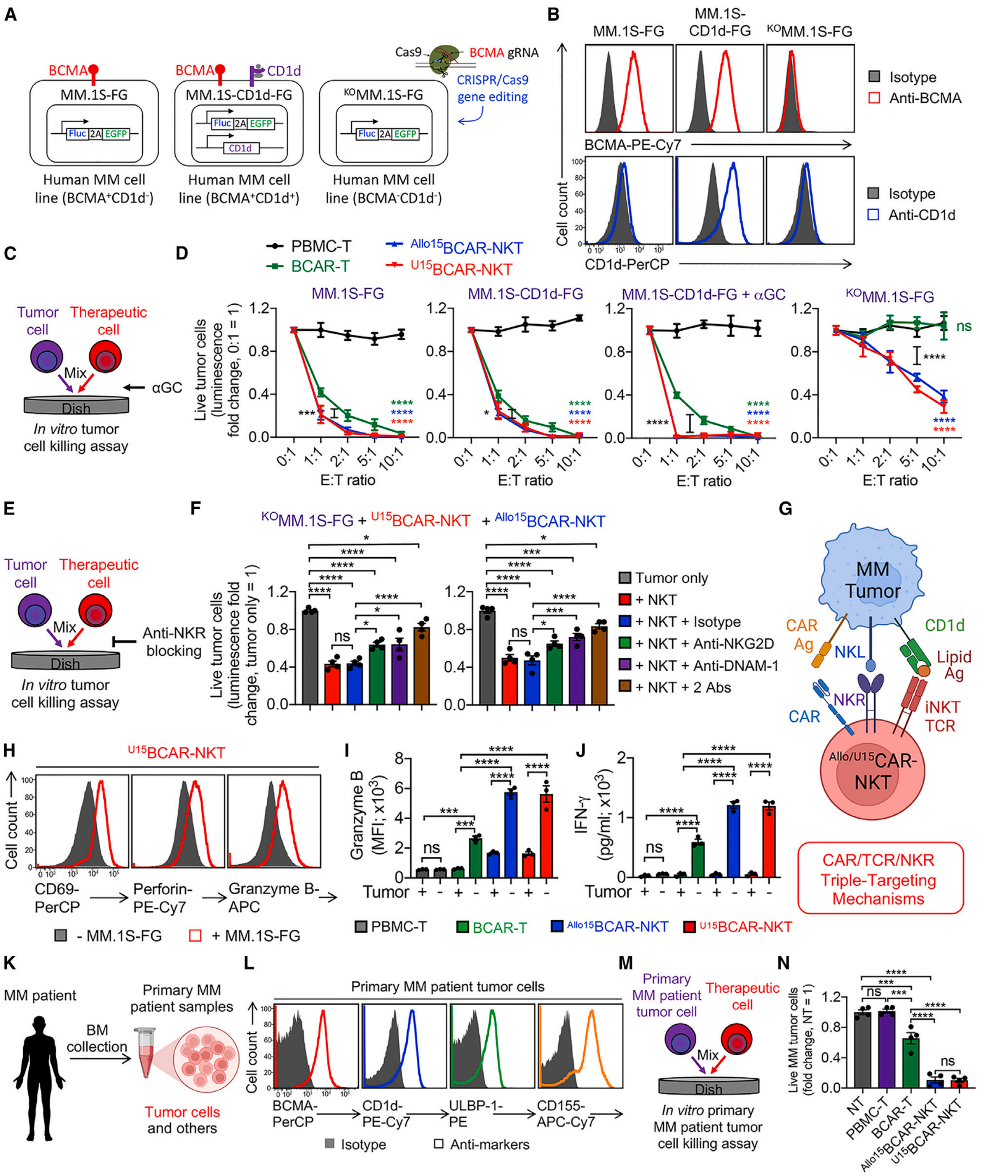

Figure 4. U15BCAR-NKT cells directly kill tumor cells at high efficacy and use multiple targeting mechanisms

(A) Schematics showing the indicated human MM.1S cell lines. MM.1S-FG, MM.1S cell line engineered to express FG dual reporters; MM.1S-FG-CD1d, MM.1S-FG cell line further engineered to overexpress human CD1d; KOMM.1S-FG, MM.1S-FG cell line further engineered to knock out the *BCMA* gene. (B) FACS detection of BCMA and CD1d

(legend continued on next page)

cells may open up new avenues for targeting hard-to-treat tumors and counteracting tumor antigen escape.

^{U}CAR-NKT cells exhibit potent antitumor efficacy *in vivo*

To assess the *in vivo* antitumor effectiveness of $^{Allo/U15}$BCAR-NKT cells, a series of *in vivo* experiments were carried out utilizing human MM xenograft NSG mouse models. Conventional BCAR-T cells were included as a benchmark control. Four separate *in vivo* experiments were conducted to replicate distinct tumor burdens (comprising low and high tumor burdens) (Figures 5A and 5E) and various tumor heterogeneity scenarios, involving BCMA^{+}CD1d^{-} MM tumor cells (Figures 5A and 5E), BCMA^{+}CD1d^{+} MM tumor cells (Figure 5I), and BCMA^{-}CD1d^{-} MM tumor cells (Figure 5M).

In a scenario characterized by a low tumor burden and the presence of BCMA^{+}CD1d^{-} tumor cells, $^{Allo/U15}$BCAR-NKT cells exhibited a tumor cell elimination efficacy on a par with that of BCAR-T cells (Figures 5B and 5C). However, mice treated with BCAR-T cells, while becoming tumor free, eventually succumbed to GvHD (Figure 5D). In contrast, mice treated with $^{Allo/U15}$BCAR-NKT cells achieved a long-term survival with both tumor eradication and an absence of GvHD (Figure 5D).

Remarkably, even under conditions of high tumor burden and BCMA^{+}CD1d^{-} tumor cells, $^{Allo/U15}$BCAR-NKT cells continued to effectively suppress tumor growth and achieved superior tumor clearance (Figures 5F and 5G). Conventional BCAR-T cells, on the other hand, exhibited a less-effective suppression of tumor growth and led to earlier mortality in experimental mice (Figures 5F–5H).

Under high tumor load conditions and BCMA^{+}CD1d^{+} tumor cells, $^{Allo/U15}$BCAR-NKT cells still effectively suppressed tumor growth and achieved tumor clearance in a portion of the experimental mice (specifically, three out of five mice for ^{Allo15}BCAR-NKT cells and three out of six for ^{U15}BCAR-NKT cells) (Figures 5J–5L). Conventional BCAR-T cells, however, displayed a less effective tumor growth suppression and failed to achieve tumor clearance (Figures 5J–5L).

In the scenario involving a low tumor burden and BCMA^{-}CD1d^{-} tumor cells, both BCAR-T cells and $^{Allo/U15}$BCAR-NKT cells demonstrated reduced antitumor efficacy due to the absence of a CAR target (Figures 5N–5P). Nevertheless, $^{Allo/U15}$BCAR-NKT cells still managed to suppress tumor growth, potentially attributable to their intrinsic capability for NKR-mediated tumor cell killing (Figures 4E–4G and 5N–5P). It is noteworthy that the inflammatory TME can potentially induce the upregulation of HLA molecules on immune cells infiltrating the tumor site, possibly through factors such as IFN-γ.[43,44] We therefore assessed HLA expressions on $^{Allo/U15}$BCAR-NKT cells collected from the BM of tumor-bearing mice, as well as those acquired from *in vitro* culture following IFN-γ stimulation (Figures S7A–S7E). In comparison with conventional T cells, $^{Allo/U15}$BCAR-NKT cells exhibited low/no expression of HLA-I and HLA-II molecules, signifying their resistance to T cell-mediated allorejection under *in vivo* or IFN-γ stimulation conditions (Figures S7B–S7E).

^{U}CAR-NKT cells maintain high antitumor efficacy despite T cell-mediated allorejection

Subsequently, we assessed the *in vivo* antitumor efficacy of $^{Allo/U15}$BCAR-NKT cells in a setting of T cell-mediated allorejection. Four days following the injection of MM.1S-FG, donor-mismatched PBMCs were introduced into the mice, simulating the presence of host T cells. Following this, therapeutic cells were administered, and their effectiveness in suppressing tumor growth was evaluated (Figure 6A).

Remarkably, ^{U15}BCAR-NKT cells continued to effectively suppress tumor growth and achieved superior tumor control, leading to improved mouse survival (Figures 6B–6D). ^{Allo15}BCAR-NKT cells exhibited a less-effective suppression of tumor growth, resulting in earlier mouse mortality (Figures 6B–6D). In contrast, conventional BCAR-T cells performed the worst, with the least-effective tumor suppression and the earliest onset of mouse mortality (Figures 6B–6D).

These results substantiate that ^{U15}BCAR-NKT cells possess a robust resistance to host T cell-mediated allorejection and maintain the highest level of antitumor efficacy. While their antitumor efficacy may experience some attenuation compared with scenarios lacking T cell-mediated allorejection (Figures 5A–5H and 6B–6D), they nonetheless exhibit significant promise for the effective treatment of cancer with a diminished susceptibility to allorejection concerns.

^{U}CAR-NKT cells alter the TME by selectively depleting TAMs and MDSCs through CD1d recognition

The immunosuppressive TME poses a substantial challenge to cancer immunotherapy, particularly in the context of solid tumors, but it is

on the indicated tumor cells. (C and D) Studying the antitumor efficacy of ^{U15}BCAR-NKT cells against human MM.1S cell lines. ^{Allo15}BCAR-NKT, BCAR-T, and non-BCAR-engineered PBMC-T cells were included as therapeutic cell controls. (C) Experimental design. (D) Tumor cell killing data at 24 h (n = 4). (E and F) Studying the tumor killing mechanism of ^{U15}BCAR-NKT cells mediated by NK activating receptors (i.e., NKG2D and DNAM-1). (E) Experimental design. (F) Tumor cell killing data at 24 h (E:T ratio = 10:1; n = 4). (G) Diagram showing the CAR/TCR/NKR triple tumor-targeting mechanisms of ^{U15}BCAR-NKT cells. (H–J) FACS characterization of ^{U15}BCAR-NKT cells 24 h after co-culturing with MM.1S-FG. (H) FACS detection of surface CD69 as well as intracellular Perforin and Granzyme B in ^{U15}BCAR-NKT cells. (I) Quantification of (H) (n = 3). (J) ELISA analyses of IFN-γ production by ^{U15}BCAR-NKT cells (n = 3). (K–N) Studying the antitumor efficacy of ^{U15}BCAR-NKT cells against primary MM patient samples. (K) Diagram showing the collection of bone marrow (BM) samples from MM patients. (L) FACS detection of CAR target (BCMA), iNKT TCR target (CD1d), and NKR target (ULBP-1 and CD155) on primary MM patient-derived tumor cells. (M) Experimental design to study the primary MM tumor cell killing by therapeutic cells. (N) Tumor cell killing data at 24 h (n = 4). Representative of 3 experiments. Data are presented as the mean ± SEM. ns, not significant; *$p < 0.05$, **$p < 0.01$, ***$p < 0.001$, ****$p < 0.0001$ by one-way ANOVA (F, I, J, and N) or two-way ANOVA (D).

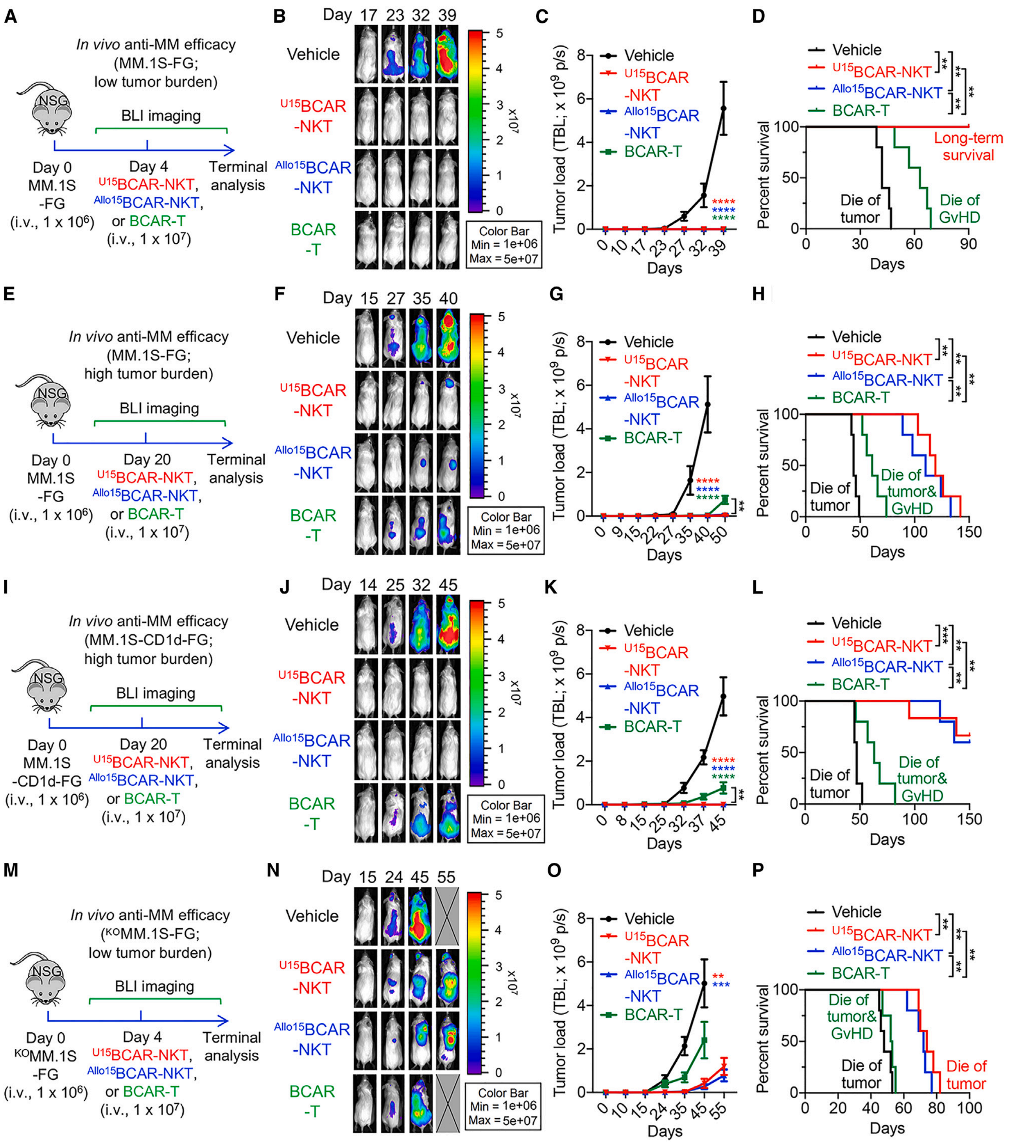

Figure 5. ^{U15}BCAR-NKT cells exhibit potent antitumor efficacy *in vivo*

(A–D) Studying the *in vivo* antitumor efficacy of ^{U15}BCAR-NKT cells in an MM.1S-FG human MM xenograft NSG mouse model. Therapeutic cells are injected on day 4 to mimic the low tumor burden condition. (A) Experimental design. (B) BLI images showing the presence of tumor cells in experimental mice over time. (C) Quantification of (B) (n = 5). TBL, total body luminescence. (D) Kaplan-Meier survival curves of experimental mice over time (n = 5). (E–H) Studying the *in vivo* antitumor efficacy of ^{U15}BCAR-NKT cells in an MM.1S-FG human MM xenograft NSG mouse model. Therapeutic cells are injected on day 20 to mimic the high tumor burden condition. (E) Experimental design. (F) BLI images showing the presence of tumor cells in experimental mice over time. (G) Quantification of (F) (n = 5). (H) Kaplan-Meier survival curves of experimental mice over

(legend continued on next page)

also pertinent to certain blood cancers such as MM, especially when the disease affects BM sites.[45–47] Within the TME, two major immunosuppressive components are tumor-associated macrophages (TAMs) and myeloid-derived suppressor cells (MDSCs).[47–50] Notably, both TAMs and MDSCs express elevated levels of CD1d, rendering them direct targets of NKT cells (Figure 7A).[45,47,51] In light of this, we conducted an investigation into the interactions between $^{Allo/U15}$BCAR-NKT cells and the MM TME, using *in vitro* cultured macrophages/MDSCs (Figure 7B) and primary MM patient samples (Figure 7K).

In the first study, we generated M2-polarized macrophages or MDSCs from healthy donor-derived monocytes (Figure 7B). These macrophages and MDSCs displayed elevated expression levels of macrophage markers, including CD11b, CD206, CD163, and HLA-DR (Figure 7C). Significantly, these cells exhibited notable levels of CD1d expression, indicating their susceptibility to targeting by NKT cells via the iNKT TCR/CD1d recognition (Figures 7D and 7E). To assess the capacity of therapeutic cells to target macrophages and MDSCs, we established an *in vitro* macrophage/MDSC assay (Figure 7F). $^{Allo/U15}$BCAR-NKT cells demonstrated effective elimination of M2 macrophages and MDSCs, particularly in the presence of αGC, a phenomenon that was impeded when CD1d was blocked (Figure 7G). Similar findings were also noted for PBMC-derived NKT (^{PBMC}NKT) cells (Figures S8A–S8C), which further validate the capacity of $^{Allo/U15}$BCAR-NKT cells to deplete macrophages and MDSCs through CD1d-antigen-iNKT TCR recognition. Significantly, even in the absence of αGC, $^{Allo/U15}$BCAR-NKT cells displayed a substantial capacity to target M2 macrophages and MDSCs, suggesting their inherent capability for NKR-mediated function (Figure 7G). The depletion of macrophages and MDSCs by $^{Allo/U15}$BCAR-NKT cells corresponded with the upregulation of cytotoxic molecules such as Granzyme B (Figure 7H). It is noteworthy that $^{Allo/U15}$BCAR-NKT cells did not exhibit cytotoxicity toward healthy donor-derived T and B cells, which is likely attributable to the absence of CD1d expression on these particular target cells (Figures 7H and 7I). In contrast, BCAR-T cells were unable to target macrophages and MDSCs, underscoring the potential of $^{Allo/U15}$BCAR-NKT cells to alter the TME (Figure 7J). Furthermore, $^{Allo/U15}$BCAR-NKT cells also demonstrated cytotoxic activity against CD1d^{+} M1-polarized macrophages, which are recognized as antitumorigenic myeloid populations (Figures S8D–S8G).[52,53] Consequently, there is an intriguing opportunity for further investigation into the potential of $^{Allo/U15}$BCAR-NKT cells to target pro-inflammatory myeloid cells in alternate contexts such as viral infections and autoimmune disorders.[54,55]

In the second study, we co-cultured $^{Allo/U15}$BCAR-NKT cells with primary MM patient BM samples (Figure 7K). $^{Allo/U15}$BCAR-NKT cells demonstrated effective and selective depletion of TAMs and MDSCs, which was due to the high expression of CD1d on TAMs and MDSCs (Figures 7L and 7M). Notably, $^{Allo/U15}$BCAR-NKT cells spared other immune cells expressing no or low level of CD1d, including granulocytes, T cells, B cells, NK cells, and HSCs (Figures 7M–7O and S8H–S8L). Further investigations were directed toward specific subpopulations of HSCs isolated from MM patient samples, including long-term HSCs, short-term HSCs, and multi-potent progenitor cells (Figure S8I). Notably, all three subpopulations of HSCs exhibited undetectable CD1d expression and were not targeted by $^{Allo/U15}$BCAR-NKT cells (Figures S8J–S8L). Preserving the normal functionality of other immune cells, particularly HSCs in the BM, is of paramount importance given their pivotal role in supporting functional hematopoiesis.[56]

^{U}CAR-NKT cells exhibit a prominent safety profile featured by minimal GvHD risk and low CRS attributes

The primary safety concern associated with allogeneic cell therapy typically revolves around the risk of GvHD.[57–61] For ^{U}CAR-NKT cells, the unique feature of their iNKT TCR recognition of the non-polymorphic MHC molecule CD1d suggests that these cells are unlikely to induce GvHD.[62,63] This characteristic was assessed through both *in vitro* MLR assays (Figure 8A) and an *in vivo* MM xenograft model (Figure 5A).

In the *in vitro* MLR assay, $^{Allo/U15}$BCAR-NKT cells were stimulated with irradiated PBMCs derived from a diverse set of mismatched healthy donors (>20 donors) (Figures 8A and 8B). This assay revealed minimal production of IFN-γ by $^{Allo/U15}$BCAR-NKT cells, in stark contrast to conventional BCAR-T cells, which exhibited robust IFN-γ production (Figure 8B).

In the *in vivo* MM NSG xenograft model, the administration of conventional BCAR-T cells led to the development of severe GvHD, ultimately resulting in the demise of experimental mice (Figure 5D). This was accompanied by substantial immune cell infiltration into critical organs such as the liver and lung (Figures 8C and 8D). In sharp contrast, the treatment with $^{Allo/U15}$BCAR-NKT cells yielded a GvHD-free, long-term survival outcome for experimental mice (Figure 5D). Importantly, this was associated with the absence of immune cell infiltration in vital organs (Figures 8C and 8D).

Cytokine release syndrome (CRS) is a substantial side effect associated with CAR-T cell therapy, and it has been documented that

time (n = 5). (I–L) Studying the *in vivo* antitumor efficacy of ^{U15}BCAR-NKT cells in an MM.1S-CD1d-FG human MM xenograft NSG mouse model. Therapeutic cells are injected on day 20 to mimic the high tumor burden condition. (I) Experimental design. (J) BLI images showing the presence of tumor cells in experimental mice over time. (K) Quantification of (J) (n = 5–6). (L) Kaplan-Meier survival curves of experimental mice over time (n = 5–6). (M–P) Studying the *in vivo* antitumor efficacy of ^{U15}BCAR-NKT cells in a ^{KO}MM.1S-FG human MM xenograft NSG mouse model. Therapeutic cells are injected on day 4 to mimic the low tumor burden condition. (M) Experimental design. (N) BLI images showing the presence of tumor cells in experimental mice over time. (O) Quantification of (N) (n = 4–5). (P) Kaplan-Meier survival curves of experimental mice over time (n = 4–5). Representative of 2 experiments. Data are presented as the mean ± SEM. ns, not significant; *p < 0.05, **p < 0.01, ***p < 0.001, ****p < 0.0001, by one-way ANOVA (G and O), two-way ANOVA (C and K), or by log rank (Mantel-Cox) test adjusted for multiple comparisons (D, H, L, and P).

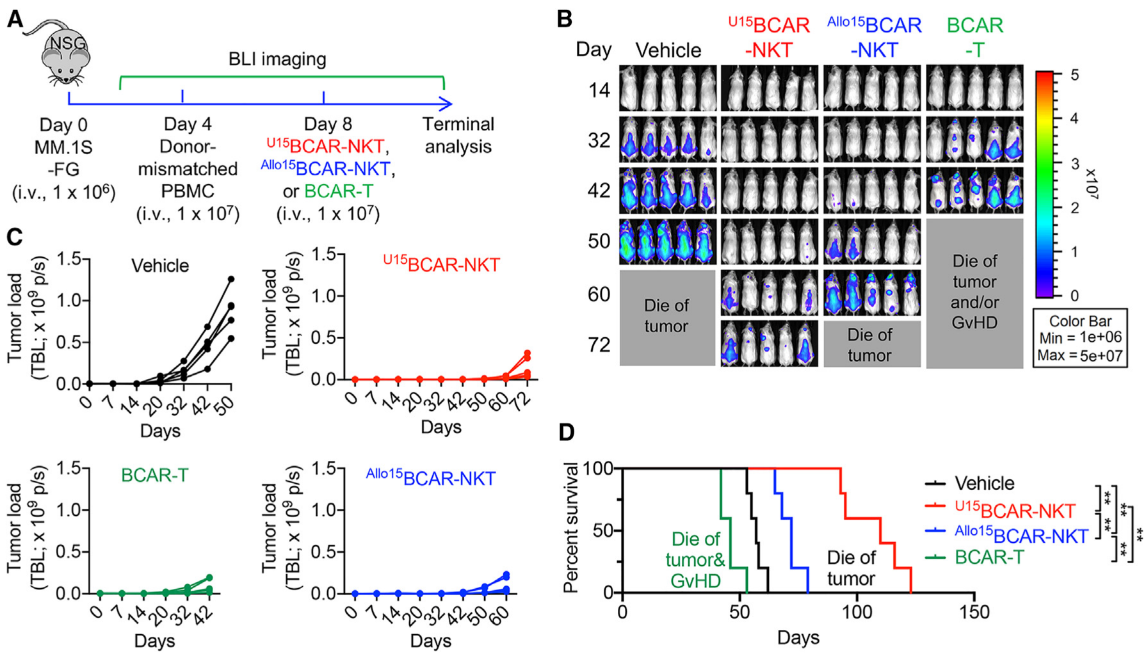

Figure 6. ^{U15}BCAR-NKT cells maintain high antitumor efficacy despite T cell-mediated allorejection

(A) Experimental design to study the *in vivo* antitumor efficacy of ^{U15}BCAR-NKT cells under T cell-mediated allorejection in a human MM xenograft NSG mouse model. ^{Allo15}BCAR-NKT and BCAR-T cells were included as controls. (B) BLI images showing the presence of tumor cells in experimental mice over time. (C) Quantification of (B) (n = 5). (D) Kaplan-Meier survival curves of experimental mice over time (n = 5). Representative of 2 experiments. Data are presented as the mean ± SEM. ns, **$p < 0.01$, by log rank (Mantel-Cox) test adjusted for multiple comparisons.

macrophages can exacerbate CRS effects.[64–66] Interestingly, the treatment with $^{Allo/U15}$BCAR-NKT cells, in contrast to BCAR-T cells, led to a reduction in the levels of CRS-related biomarkers, such as IL-6 and serum amyloid A-3 (SAA-3), in the serum (Figures 8C and 8D).[64–66] These results indicate that ^{U}CAR-NKT cells may carry a lower risk of CRS-like response, a characteristic that can be attributed to their NK cell attributes (Figures 2D, 2E, S4, and S5)[67–69] and their ability to deplete macrophages associated with CRS (Figure 7).[64–66]

In summary, these findings collectively point to a favorable safety profile for ^{U}CAR-NKT cells. They exhibit low CRS-like response and minimal GvHD risk, thus strengthening their potential for off-the-shelf therapeutic applications.

^{U}CAR-NKT cells can be further engineered with a safety switch

While our studies did not reveal any tissue toxicity induced by $^{Allo/U15}$BCAR-NKT cells in our NSG xenograft mouse models (Figures 8C and 8D), it is important to acknowledge that these safety assessments may have limitations associated with the chosen preclinical animal models. As such, additional safety measures, particularly in the early stages of clinical development, may be warranted. To address this concern, we have implemented a "safety switch" in universal BCAR-NKT cell products by introducing a suicide gene (i.e., sr39TK) into the human iNKT TCR gene delivery vector (Figure S9A). This engineering results in universal BCAR-NKT cells that are entirely labeled with the suicide gene (denoted as ^{U}BCAR-NKT-TK cells) (Figure S9A). In cell culture, the addition of a guanosine analog, such as ganciclovir (GCV), effectively led to the elimination of ^{U}BCAR-NKT-TK cells (Figure S9B). Furthermore, in an NSG mouse xenograft model, the administration of GCV successfully depleted ^{U}BCAR-NKT-TK cells from all examined tissues, including the blood, liver, spleen, and lung (Figures S9C–S9E). It is noteworthy that GCV has already been utilized clinically as a prodrug to induce the sr39TK-mediated suicide effect in cellular products.[70–72] In addition, other alternative suicide switch systems, such as inducible Cas9 and truncated EGFR, can also be employed.[73–75]

Taken together, our results indicate that universal BCAR-NKT cells pose no risk of GvHD, low risk of CRS, and can be equipped with an additional safety switch, rendering them well-suited for off-the-shelf allogeneic cell therapy.

^{U}CAR-NKT cells resist NK cell-mediated allorejection

In addition to T cell-mediated allorejection, host NK cells can contribute to allorejection through a dual-trigger mechanism. This mechanism involves two aspects: (1) "missing self," which occurs when there is a lack of matching HLA-I molecules on allogeneic cells, triggering the release of inhibitory signals mediated by killer cell immunoglobulin-like receptors. (2) "Stress signals," which involve the upregulation of stress molecules on allogeneic cells, inducing the activation of NKRs such as NKG2D (Figure S10A).[21,37,76]

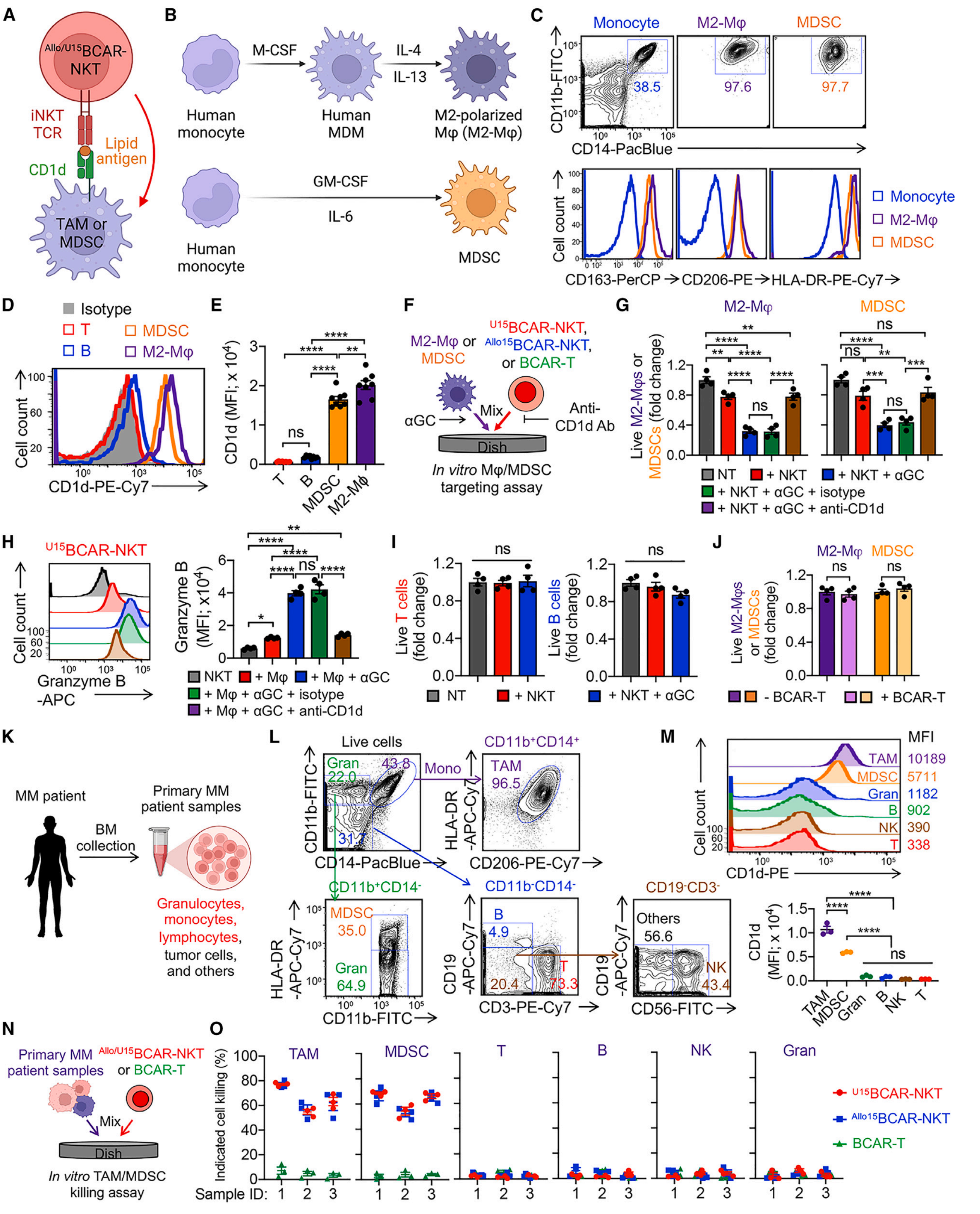

(legend on next page)

To assess the allorejection prospect of $^{Allo/U15}$BCAR-NKT cells, especially HLA-I-ablated ^{U15}BCAR-NKT cells, we conducted *in vitro* MLR assays, specifically designed to investigate NK cell-mediated allorejection (Figure S10B). In these assays, $^{Allo/U15}$BCAR-NKT cells were co-cultured with donor-mismatched PBMC-NK cells (>10 donors), followed by quantifying viable $^{Allo/U15}$BCAR-NKT cells (Figure S10B). Compared with BCAR-T cells, $^{Allo/U15}$BCAR-NKT cells exhibited markedly improved survival rates (Figure S10C), which can be attributed to their reduced expression of surface NK ligands, such as ULBP and MICA/B (Figure S10D). This diminished NK ligand expression may contribute to their resistance to NK cell-mediated allorejection. In summary, these results reveal an intriguing "hypoimmunogenic" feature of $^{Allo/U15}$BCAR-NKT cells, potentially providing advantages for their off-the-shelf cell therapy applications.

^{U}CAR-NKT cells can be engineered with HLA-E to further ensure resistance to NK cell-mediated allorejection

Although NK cell-mediated allorejection of ^{U15}BCAR-NKT cells was not observed, it is important to acknowledge potential limitations in our immunogenicity studies using preclinical assays (Figures S10B–S10D). Therefore, we have taken additional steps to enhance their resistance to allogeneic NK cells. Specifically, we have engineered HLA-E, which has been reported to bind with CD94/NKG2A on NK cells and leads to inhibitory signaling within the NK cell,[77] on universal BCAR-NKT cells to prevent NK cell-mediated allorejection (Figures S11A and S11B).

CD34^{+} HSCs were gene engineered with a Lenti/iNKT-BCAR-HLA-E vector together with a CRISPR-Cas9/B2M-CIITA-gRNAs complex to ablate surface expression of HLA-I/II and achieve overexpression of HLA-E on BCAR-NKT cells, referred to as ^{UE}BCAR-NKT cells (Figures S11A–S11E). Importantly, as all therapeutic genes (iNKT TCR, BCAR, and HLA-E) are co-delivered by the same lentivector, the resulting ^{UE}BCAR-NKT cells co-expressed HLA-E, ensuring the purity and clonality of these cellular products (Figures S11D and S11F). These engineered cells exhibited similar development, phenotype, and yield to non-HLA-E-engineered universal BCAR-NKT cells (Figures 1E–1J and S11C–S11H). Furthermore, ^{UE}BCAR-NKT cells have demonstrated resistance to host T and NK cell-mediated allorejection, along with potent antitumor activity against various cancer cells (Figures S11I–S11L). In conclusion, these results underscore the feasibility and cancer therapy potential of ^{UE}BCAR-NKT cell products, setting the stage for their translational and clinical development.

DISCUSSION

In this study, we present a technological advancement aimed at facilitating the generation of HLA-ablated ^{U}CAR-NKT cells. This process is achieved through the genetic modification of HSCs and the establishment of a feeder-free differentiation culture system. Our technology has demonstrated the capability to produce ^{U}CAR-NKT cells with a high degree of yield, purity, and robustness (Figure 1). These HLA-ablated ^{U}CAR-NKT cells exhibit remarkable resistance to allorejection mediated by host T and NK cells (Figures 3, S3, S10, and S11), which addresses a significant limitation of conventional CAR-T cell therapy,[5,18] as these cells can persist *in vivo* longer and exert a sustained tumor-suppressive effect (Figure 6). The resulting ^{U}CAR-NKT cells exhibit several favorable characteristics for the development of off-the-shelf cancer immunotherapies. They demonstrate potent antitumor efficacy through multiple mechanisms (Figures 4 and 5), possess a high safety profile characterized by a minimal risk of GvHD and low CRS attributes, and can be further engineered with a suicide switch for added safety (Figure 8). Moreover, these ^{U}CAR-NKT cells exhibit the intriguing capacity to modulate the immunosuppressive TME by selectively depleting CD1d^{+} TAMs and MDSCs (Figure 7). This technological advancement holds significant promise in the field of cancer immunotherapy, offering a multifaceted approach to enhance the effectiveness and safety of off-the-shelf cellular therapies.

The off-the-shelf HSC-derived CAR-NKT technology demonstrates remarkable versatility, as demonstrated by its successful application in the generation of six distinct CAR-NKT cell products through various gene engineering approaches (Figure S2A). These modifications encompass the incorporation of diverse genes, including those conferring immune enhancement (e.g., IL-15) (Figures 1A and 1B), the integration of suicide switches (e.g., sr39TK) (Figure 8G), the introduction of reporter genes (e.g., FG) (Figures 3G and 3H), and the incorporation of NK inhibitory factors (e.g., HLA-E) (Figures S11A and S11B). In addition, this technology allows for the targeted ablation of specific genes, such as HLA molecules (Figures 1A, 1B, 8G, S11A, and S11B). Notably, the incorporation of these various cargo genes did not result in any discernible disruptions in the manufacturing process, yield, or quality of the

Figure 7. ^{U15}BCAR-NKT cells alter the TME by selectively depleting TAMs and MDSCs via CD1d recognition

(A) Diagram showing the TAM/MDSC targeting by ^{U15}BCAR-NKT cells via CD1d/iNKT TCR recognition. (B–J) Study the TAM/MDSC targeting by ^{U15}BCAR-NKT cells using *in vitro* cultured cells. (B) Diagram showing the generation of healthy donor PBMC-derived TAMs and MDSCs. MDM, monocyte-derived macrophage; Mφ, macrophage. (C) FACS detection of macrophage markers on the indicated cells. (D) FACS detection of CD1d on the indicated cells. (E) Quantification of (D). (F) Experimental design to study Mφ/MDSC targeting by ^{U15}BCAR-NKT cells using an *in vitro* Mφ/MDSC targeting assay. (G) Mφ/MDSC killing data at 24 h (n = 4). (H) FACS analyses of Granzyme B production by ^{U15}BCAR-NKT cells 24 h after co-culturing with Mφ. (I) T and B cell killing data by ^{U15}BCAR-NKT cells at 24 h (n = 4). (J) Mφ/MDSC killing data by BCAR-T cells at 24 h (n = 4). (K–O) Study the TAM/MDSC targeting by ^{U15}BCAR-NKT cells using primary MM patient samples. (K) Schematics showing the collection of primary MM patient samples. (L) FACS analysis of immune cell composition in the MM patient BM samples. Gran, granulocyte; Mono, monocytes. (M) FACS analyses of surface CD1d expression in the indicated TME cell component. MFI, mean florescence intensity. (N) Experimental design to study TME targeting by ^{U15}BCAR-NKT cells. (O) Killing data of the indicated TME cell component (n = 3). Representative of 3 experiments. Data are presented as the mean ± SEM. ns, not significant; *$p < 0.05$, **$p < 0.01$, ***$p < 0.001$, ****$p < 0.0001$ by Student's t test (J), or one-way ANOVA (E, G, H, I, and M).

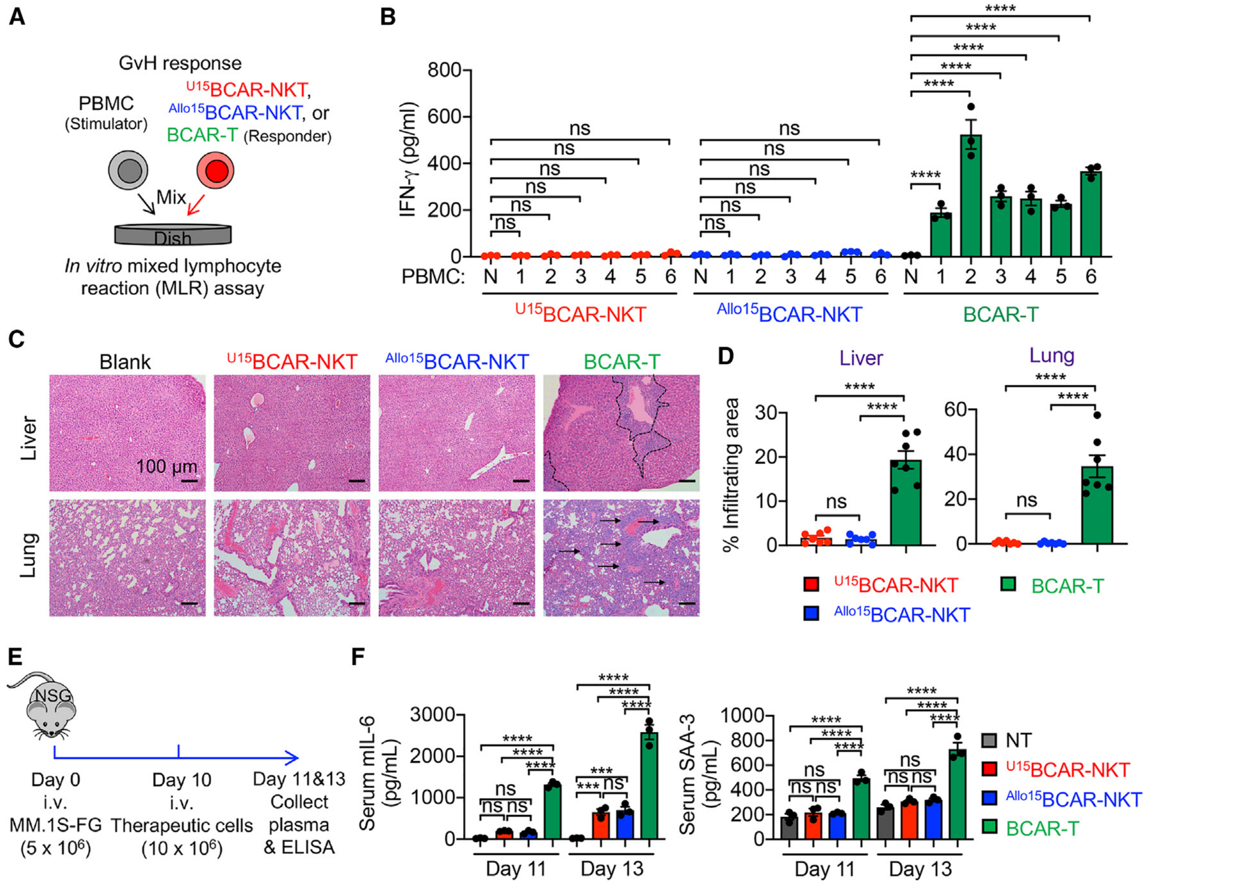

Figure 8. U15BCAR-NKT cells exhibit a high safety profile featured by low CRS attributes and minimal GvHD risk

(A and B) Studying the graft-versus-host response of U15BCAR-NKT cells using an *in vitro* MLR assay. PBMCs from over 10 random mismatched healthy donors were used as stimulator cells. Data from six representative donors are presented. Allo15BCAR-NKT and conventional BCAR-T cells were included as responder controls. (A) Experimental design. (B) ELISA analyses of IFN-γ production on day 4. N, no addition of stimulator PBMCs (n = 3). (C and D) Studying the GvHD risk of U15BCAR-NKT cells using a human MM.1S xenograft NSG mouse model. Experimental design is shown in Figure 5A. (C) H&E-stained tissue sections. Tissues were collected from experimental mice on day 60. Scale bars, 100 μm. (D) Quantification of (C) (n = 7). (E and F) Studying CRS response induced by U15BCAR-NKT cells using an *in vivo* human MM.1S xenograft NSG mouse model. (E) Experimental design. (F) ELISA analyses of mouse IL-6 and SAA3 in mouse plasma collected on days 11 and 13 (n = 3). SAA-3, serum amyloid A-3. NT, mouse plasma sample collected from tumor-bearing mice receiving no therapeutic cell treatment. Representative of 3 experiments. Data are presented as the mean ± SEM. ns, not significant, $*p < 0.05$, $**p < 0.01$, $***p < 0.001$, $****p < 0.0001$, by one-way ANOVA (B, D, and F).

UCAR-NKT cell products (Figures 1E–1J and S11D–S11H). This significant achievement underscores the wide-ranging potential of the technology, enabling its application to produce UCAR-NKT cell products armed with diverse CARs to target a broad spectrum of blood cancers and solid tumors, and potentially extending to other diseases.[7]

Currently, a diverse range of allogeneic off-the-shelf CAR-engineered cell therapies have been developed and are rapidly advancing as a frontier in cancer immunotherapy. Initially, conventional αβ CAR-T cells have been subjected to gene editing techniques, such as disruption of TRAC and/or TRBC loci, to prevent GvHD induced by HLA incompatibility.[8] Additional modifications include disrupting HLA-I and/or HLA-II molecules to mitigate rejection by host T cells and disrupting CD52 to make these allogeneic T cells resistant to lymphodepleting drugs such as alemtuzumab.[8,78] Secondly, NK cell-based allogeneic cell products have been developed, which are considered to carry a lower risk of GvHD and, therefore, do not require extensive gene editing.[79,80] However, their clonal expansion and effectiveness against tumors *in vivo* might be more limited compared with conventional αβ T cells. Currently, various such conventional αβ T- and NK-based allogeneic cell products have undergone phase I clinical trials, targeting B cell malignancies, acute myeloid leukemia, MM, and specific solid tumors.[8,80–84] These studies demonstrate the feasibility, antitumor activity, and manageable safety profile of these therapies, marking a significant advancement in the field of allogeneic cell therapy. In addition, some preclinical studies have explored the use of other immune cell types, such as NKT, mucosal-associated invariant T cells, and γδ T cells, as well as alternative cell sources such as induced pluripotent stem cell-derived

NK or T cells.[19,20,85–87] Diverse hypoimmunogenic cell products have been developed and have shown promise for off-the-shelf cancer immunotherapy.[88]

The reported technology employs a feeder-free culture method that is amenable to easy scaling for clinical and commercial development. The resultant CAR-NKT cell products demonstrate remarkable attributes, including high yield, purity, and robustness (Figure 1). Importantly, there is an absence of bystander conventional αβ T cells (Figures 1E and 1G), eliminating the need for supplementary purification steps. Significantly, due to the inherent property of NKT cells not to recognize mismatched HLAs, the elimination of endogenous TCR is rendered unnecessary, thereby preserving the TCR and its associated advantages for CAR-NKT cells.[89] Notably, an unintended characteristic of non-HLA-ablated ^{Allo15}BCAR-NKT cells is their notably lower immunogenicity when compared with conventional αβ T cells (Figure 3). ^{Allo15}BCAR-NKT cells express diminished levels of HLA-I molecules and nearly undetectable levels of HLA-II molecules, which appear to be genetically predetermined and remain stable throughout *in vitro* culture and *in vivo* persistence, even within the TME (Figures 3 and S7). This unique feature may confer resistance to allorejection by host T cells, thereby reducing the necessity for additional HLA gene editing or intensive preconditioning treatments targeting host T cell depletion, such as CD52 antibody treatment. Furthermore, a recent study has documented distinct characteristics of NKT cells, where allogeneic NKT cells exhibit prolonged persistence in MHC-mismatched canine recipients, along with sustained immunomodulatory effects.[90] This discovery further advances the potential application of allogeneic NKT cells as a readily accessible universal platform for the treatment of cancer and other diseases.

The ^{U}CAR-NKT cell products exhibit remarkable antitumor efficacy against a wide spectrum of cancers, including both hematologic malignancies and solid tumors, encompassing various tumor cell lines and primary samples from cancer patients (Figures 4, 5, 6, S4, S11K, and S11L). This exceptional potency is attributed to the robust cytotoxicity, potent effector functions, and multiple tumor-targeting mechanisms of these ^{U}CAR-NKT cells, which are acquired during the manufacturing process and persist *in vivo* (Figures 2, 4, and S6). Notably, their potent antitumor efficacy paralleled or surpassed that of PBMC-derived conventional CAR-T (Figures 4 and 5), CAR-NK (Figure S12), and CAR-NKT cells (Figure S13), underscoring their considerable promise as a potent antitumor therapy. Furthermore, in comparison with conventional CAR-T cells, ^{U}CAR-NKT cells possess an additional distinct characteristic that equips them to overcome the current limitations encountered by CAR-T cells in targeting the TME.[6,91,92]

The immunosuppressive TME, consisting of TAMs and MDSCs, presents a significant challenge to the effectiveness of CAR T cell-based immunotherapy. Current strategies aimed at modulating TAMs and MDSCs have often proven to be suboptimal, typically involving approaches to reduce monocyte recruitment and induce an antitumor M1-like transformation.[93–96] Remarkably, ^{U}CAR-NKT cells have demonstrated the ability to alter the TME by effectively and selectively depleting CD1d-high TAMs and MDSCs (Figure 7). Importantly, the treatment with ^{U}CAR-NKT cells preserves the integrity of other blood cells, including HSCs, T, B, and NK cells, and granulocytes, maintaining the host's hemostasis and functional immunity (Figures 7 and S8). It is noteworthy that, in addition to M2-polarized macrophages, CD1d^{+} M1-polarized macrophages can also be targeted by ^{U}CAR-NKT cells (Figures S8D–S8G). These M1-polarized macrophages exhibit both pro-inflammatory and antitumor properties.[52,53] Consequently, the ability of ^{U}CAR-NKT cells to deplete all myeloid populations may pose certain limitations for cancer immunotherapy. Nonetheless, this observation prompts an intriguing avenue for research into leveraging ^{U}CAR-NKT cells to target pro-inflammatory myeloid cells in different disease contexts, such as viral infections and autoimmune disorders.[54,55]

While the reported technology holds significant promise, it faces certain limitations that necessitate further refinement. In this study, T and NK cell-mediated allorejections were explored through *in vitro* MLR assays and/or *in vivo* xenograft NSG mouse models (Figures 3C, 3D, 3F–3J, S3C, 6A–6D, and S10B–S10D). However, it is crucial to acknowledge that these assays only partially replicate T and NK cell-mediated alloresponse, and the true conditions can only be fully understood through clinical studies. IL-15 integration has been a prevalent feature across various CAR-NKT cell formulations, with clinical trials showcasing enhanced *in vivo* functionality of IL-15-engineered CAR-NKT cells.[13,15,97] Our investigation substantiates the efficacy of IL-15 on CAR-NKT cells, specifically highlighting improvements in long-term antitumor activity and overall performance (Figure S14). In addition, incorporating genes encoding other immune-enhancing molecules (such as IL-7, IL-12, IL-18, and IL-21) and immunosuppression-resistant factors (such as immune checkpoint inhibitors such as anti-PD-1 antibody and dominant-negative TGF-β receptor) into ^{U}CAR-NKT cells could enhance their antitumor capabilities.[3] Furthermore, synergistic approaches involving other therapeutic modalities such as checkpoint blockade therapy, preconditioning regimens, and cancer vaccines could be explored to improve the *in vivo* performance of ^{U}CAR-NKT cells.[84] Considering the potential immunogenicity induced by sr39TK, alternative suicide switch systems, such as inducible Cas9 and truncated EGFR, could be integrated to enhance the safety profile of ^{U}CAR-NKT cells.[73,98] In summary, comprehensive clinical investigations are essential to fully assess the potential of ^{U}CAR-NKT cells as allogeneic candidates for off-the-shelf cancer therapy. Continued research and refinement are crucial to address the identified limitations and unlock the full therapeutic potential of this innovative approach.

MATERIALS AND METHODS

Mice

NOD.Cg-Prkdc^{SCID}Il2rg^{tm1Wjl}/SzJ (NOD/SCID/IL-2Rγ$^{-/-}$, NSG) mice were maintained in the animal facilities of the University of California, Los Angeles (UCLA). Six- to 10-week-old female mice were used for all experiments. All animal experiments were approved by

the Institutional Animal Care and Use Committee (IACUC) of UCLA. All mice were bred and maintained under specific pathogen-free conditions, and all experiments were conducted in accordance with the animal care and use regulations of the Division of Laboratory Animal Medicine (DLAM) at the UCLA.

Media and reagents

The X-VIVO 15 Serum-Free Hematopoietic Cell Medium was purchased from Lonza. The StemSpan T cell Generation Kit, comprising the StemSpan SFEM II Medium, the StemSpan Lymphoid Progenitor Expansion Supplement, the StemSpan Lymphoid Progenitor Maturation Supplement, the StemSpan Lymphoid Progenitor Differentiation Coating Material, and the ImmunoCult Human CD3/CD28/CD2 T Cell Activator, was purchased from STEMCELL Technologies. The CTS OpTmizer T Cell Expansion SFM (no phenol red, bottle format), the RPMI 1640 cell culture medium, and the DMEM cell culture medium were purchased from Thermo Fisher Scientific. The CryoStor Cell Cryopreservation Media CS10 was purchased from MilliporeSigma.

The homemade C10 medium was made of RPMI 1640 cell culture medium, supplemented with fetal bovine serum (FBS) (10% v/v), penicillin-streptomycin-glutamine (P/S/G) (1% v/v), MEM non-essential amino acids (NEAA) (1% v/v), HEPES (10 mM), sodium pyruvate (1 mM), β-mercaptoethanol (β-ME) (50 mM), and Normocin (100 mg/mL). The homemade D10 medium was made of DMEM supplemented with FBS (10% v/v), P/S/G (1% v/v), and Normocin (100 mg/mL). The homemade R10 medium was made of RPMI supplemented with FBS (10% v/v), P/S/G (1% v/v), and Normocin (100 mg/mL).

αGC (KRN7000) was purchased from Avanti Polar Lipids. Recombinant human IL-2, IL-3, IL-7, IL-15, IL-21, IFN-γ, Flt3 ligand (Flt3L), stem cell factor (SCF), and thrombopoietin (TPO) were purchased from PeproTech. GCV, FBS, and β-ME were purchased from Sigma. P/S/G, MEM NEAA, HEPES buffer solution, and sodium pyruvate were purchased from Gibco. Normocin was purchased from InvivoGen. GCV was purchased from Sigma.

Lentiviral vectors

Lentiviral vectors used in this study were all constructed from a parental lentivector pMNDW.[99,100] The 2A sequences derived from foot-and-mouth disease virus (F2A), porcine teschovirus-1 (P2A), and thosea asigna virus (T2A) were used to link the inserted genes to achieve co-expression.

Seven lentivectors were constructed and used in this study. The Lenti/iNKT-BCAR-IL-15 vector was constructed by inserting into the pMNDW vector a synthetic tetracistronic gene encoding human iNKT TCRα-F2A-TCRβ-P2A-BCAR-T2A-IL15 (BCAR indicates a BCMA-targeting CAR,[26] and IL15 indicates the secreting form of human IL-15). The Lenti/iNKT-BCAR-sr39TK vector was constructed by inserting into the pMNDW vector a synthetic tetracistronic gene encoding human iNKT TCRα-F2A-TCRβ-P2A-BCAR-T2A-sr39TK (sr39TK indicates an sr39TK suicide and positron emission tomography imaging reporter gene). The Lenti/iNKT-BCAR-HLA-E vector was constructed by inserting into the pMNDW vector a synthetic tetracistronic gene encoding human iNKT TCRα-F2A-TCRβ-P2A-BCAR-T2A-HLA-E. The Lenti/BCAR vector was constructed by inserting into the pMNDW a synthetic gene encoding BCAR. The Lenti/FG vector was constructed by inserting into the pMNDW a synthetic bicistronic gene encoding Fluc-P2A-EGFP.[25] The Lenti/CD1d vector was constructed by inserting into the pMNDW a synthetic gene encoding human CD1d.[25] The Lenti/BCMA vector was constructed by inserting into the pMNDW a synthetic gene encoding BCMA.

The synthetic gene fragments were obtained from GenScript and IDT. Lentiviruses were produced using human embryonic kidney 293T (HEK293T) cells (American Type Culture Collection [ATCC]), following a standard transfection protocol using the Trans-IT-Lenti Transfection Reagent (Mirus Bio) and a centrifugation concentration protocol using the Amicon Ultra Centrifugal Filter Units, according to the manufacturer's instructions (MilliporeSigma).

Cell lines

Human MM cell line MM.1S, chronic myelogenous leukemia cell line K562, Burkitt's lymphoma cell line RAJI, acute lymphoblastic leukemia cell line NALM-6, acute myeloid leukemia cell line THP1, melanoma cell line A375, lung cancer cell lines H226, A549, H292, and HCC827, ovarian cancer cell lines OVCAR3, OVCAR8, and SKOV3, pancreatic cancer cell lines ASPC1 and CAPAN2, prostate cancer cell line PC3, glioblastoma cell line U87-MG, breast cancer cell line MDA-MB-231, hepatocellular carcinoma cell lines HEPG2 and HEP3B, and HEK293T were purchased from the ATCC.

To make stable tumor cell lines overexpressing human CD1d, and/or firefly luciferase and enhanced green fluorescence protein dual reporters (FG), the parental tumor cell lines were transduced with lentiviral vectors encoding the intended gene(s). Seventy-two hours post lentivector transduction, cells were subjected to flow cytometry sorting to isolate gene-engineered cells for making stable cell lines. Nine stable tumor cell lines were generated for this study, including MM-FG, MM-CD1d-FG, K562-FG, RAJI-FG, NALM-6-FG, THP1-FG, A375-FG, H292-FG, HCC827-FG, OVCAR3-FG, OVCAR8-FG, SKOV3-FG, ASPC1-FG, CAPAN2-FG, PC3-FG, U87-MG-FG, MDA-MB-231-FG, HEPG2-FG, and HEP3B-FG cell lines. The ^{KO}MM.1S-FG cell line was generated by knocking out the BCMA gene from the parental MM.1S-FG cell line using CRISPR-Cas9. The single guide RNA targeting the BCMA gene (UAUUAAGC UCAGUCCCAAAC[101]) was purchased from Synthego, and was introduced into MM.1S-FG cells via electroporation using an Amaxa 4D Nucleofection X Unit (Lonza), according to the manufacturer's instructions.

The aAPC was generated by engineering the K562 human chronic myelogenous leukemia cell line (ATCC) to overexpress human CD83/CD86/4-1BBL co-stimulatory receptors.[102] The

aAPC-BCMA cell lines were generated by further engineering the parental aAPC line to overexpress human BCMA.

Human CB CD34$^+$ HSCs, PBMCs, and primary MM patient BM samples

Purified CB-derived human CD34$^+$ HSCs were purchased from HemaCare. Healthy donor PBMCs were provided by the UCLA/CFAR Virology Core Laboratory without identification information under federal and state regulations. Primary MM patient BM samples were collected at the Ronald Reagan UCLA Medical Center from consented patients through an IRB-approved protocol (IRB no. 21-001444) and processed.

Antibodies and flow cytometry

Fluorochrome-conjugated antibodies specific for human CD45 (clone H130, PerCP, FITC or Pacific Blue-conjugated, 1:500), TCR αβ (clone I26, Pacific Blue or PE-Cy7-conjugated, 1:25), CD3 (clone HIT3a, Pacific Blue, PE, or PE-Cy7-conjugated, 1:500), CD4 (clone OKT4, PE-Cy7, PerCP, or FITC-conjugated, 1:500), CD8 (clone SK1, PE, APC-Cy7, or APC-conjugated, 1:300), CD45RO (clone UCHL1, APC-Cy7-conjugated, 1:100), CD161 (clone HP-3G10, PerCP-conjugated, 1:50), CD69 (clone FN50, PE-Cy7 or PerCP-conjugated, 1:50), CD56 (clone HCD56, FITC or PerCP-conjugated, 1:10), CD1d (clone 51.1, PE-Cy7 or APC-conjugated, 1:50), BCMA (19F2, PE-Cy7-conjugated, 1:50), CD14 (clone HCD14, Pacific Blue-conjugated, 1:100), CD19 (clone HIB19, APC-Cy7-conjugated, 1:200), CD11b (clone ICRF44, PerCP or FITC-conjugated, 1:500), MICA/MICB (clone 6D4, APC-conjugated, 1:25), NKG2D (clone 1D11, PE-Cy7-conjugated, 1:50), DNAM-1 (clone 11A8, APC-conjugated, 1:50), NKp30 (clone P30-15, APC-conjugated, 1:50), NKp44 (clone P44-8, PE-Cy7-conjugated, 1:50), NKp46 (clone 9E2, PerCP-conjugated, 1:50), CD155 (clone SKII.4, PE-Cy7-conjugated, 1:250), CD163 (clone GHI/61, APC-Cy7-conjugated, 1:500), CD206 (clone 15-2, APC-conjugated, 1:500), IFN-γ (clone B27, PE-Cy7-conjugated, 1:50), Granzyme B (clone QA16A02, APC-conjugated, 1:2,000 or 1:5,000), Perforin (clone dG9, PE-Cy7-conjugated, 1:50 or 1:100), TNF-α (clone Mab11, APC-conjugated, 1:4,000), IL-2 (clone MQ1-17H12, APC-Cy7-conjugated, 1:50), β2-microglobulin (B2M) (clone 2M2, FITC, APC, or PerCP-conjugated, 1:2,000 or 1:5,000), HLA-DR (clone L243, APC-Cy7-conjugated, 1:200 or 1:500), and HLA-DR, DP, DQ (clone Tü 39, APC-Cy7-conjugated, 1:200 or 1:500) were purchased from BioLegend. Fluorochrome-conjugated antibodies specific for human CD34 (clone 581) and human iNKT TCR Vα24-Jβ18 (clone 6B11, PE-conjugated, 1:20) were purchased from BD Biosciences. Fluorochrome-conjugated antibody specific for human iNKT TCR Vβ11 (APC-conjugated, 1:50) was purchased from Beckman-Coulter. Fluorochrome-conjugated antibodies specific for human ULBP-1 (clone 170818, PE-conjugated, 1:25) and ULBP-2,5,6 (clone 165903, APC-conjugated, 1:25) were purchased from R&D Systems. A goat anti-mouse IgG F(ab')2 secondary antibody was purchased from Thermo Fisher Scientific. Fixable Viability Dye eFluor506 (e506, 1:500) was purchased from Affymetrix eBioscience. Mouse Fc Block (anti-mouse CD16/32) was purchased from BD Biosciences, and human Fc Receptor Blocking Solution (TrueStain FcX) was purchased from BioLegend.

All flow cytometry staining was performed following standard protocols, as well as specific instructions provided by the manufacturer of a particular antibody. Stained cells were analyzed using a MACSQuant Analyzer 10 flow cytometer (Miltenyi Biotech), following the manufacturer's instructions. FlowJo software version 9 (BD Biosciences) was used for data analysis.

Enzyme-linked immunosorbent cytokine assays

The enzyme-linked immunosorbent cytokine assays (ELISAs) for detecting human cytokines were performed following a standard protocol from BD Biosciences.[25] Supernatants from cell culture assays were collected and assayed to quantify human IFN-γ, TNF-α, IL-2, and IL-4. The capture and biotinylated pairs for detecting cytokines were purchased from BD Biosciences. The streptavidin-HRP conjugate was purchased from Invitrogen. Human cytokine standards were purchased from eBioscience. Tetramethylbenzidine substrate was purchased from KPL. Human IL-15 was quantified using a Human IL-15 Quantikine ELISA Kit (R&D Systems), following the manufacturer's instructions. Human IL-17a was quantified using a Human IL-17A ELISA MAX Deluxe Kit (BioLegend), following the manufacturer's instructions. Mouse IL-6 was quantified with paired purified anti-mouse IL-6 antibody and biotin anti-mouse IL-6 antibody (BioLegend). Mouse SAA-3 was quantified using a Mouse SAA-3 ELISA Kit (MilliporeSigma), as per the manufacturer's instructions. The samples were analyzed for absorbance at 450 nm using an Infinite M1000 microplate reader (Tecan).

Generation of HSC-derived BCAR-engineered NKT cells and their derivatives (denoted as $^{Allo/U15}$BCAR-NKT cells)

$^{Allo/U15}$BCAR-NKT cells were generated by differentiating gene-engineered CB CD34$^+$ HSCs in a five-stage feeder-free *Ex Vivo* HSC-Derived NKT Cell Culture. ^{Allo15}BCAR-NKT cells were differentiated from HSCs engineered to overexpress a human transgenic iNKT TCR, together with a BCAR and the secreting form of human IL-15. ^{U15}BCAR-NKT cells were differentiated from HSCs engineered to overexpress iNKT TCR, BCAR, and IL-15, and to ablate HLA-I/II expression. ^{U}BCAR-NKT-TK cells were differentiated from HSCs engineered to overexpress iNKT TCR, BCAR, and sr39TK, and to ablate HLA-I/II expression. ^{UE}BCAR-NKT cells were differentiated from HSCs engineered to overexpress iNKT TCR, BCAR, and HLA-E, and to ablate HLA-I/II expression.

At stage 0, frozen-thawed human CD34$^+$ HSCs were revived in X-VIVO 15 Serum-Free Hematopoietic Stem Cell Medium supplemented with 50 ng/mL Flt3L, 50 ng/mL SCF, 50 ng/mL TPO, and 20 ng/mL IL-3 for 24 h, then transduced with lentiviruses for another 24 h following an established protocol.[21,25] For ^{U15}BCAR-NKT cell generation, HSCs were further electroporated with a CRISPR-Cas9/B2M-CIITA-gRNAs complex, following an established protocol.[21]The gRNA sequences are CGCGAGCACAGCUAAGGCCA (B2M) and GAUAUUGGCAUAAGCCUCCC (CIITA).

At stage 1, gene-engineered HSCs collected from stage 0 were cultured in the feeder-free StemSpan SFEM II Medium supplemented with StemSpan Lymphoid Progenitor Expansion Supplement for ~2 weeks. CELLSTAR 24-well Cell Culture Nontreated Multiwell Plates (VWR) were used to culture HSCs. The plates were coated with 500 μL/well StemSpan Lymphoid Differentiation Coating Material for 2 h at room temperature or overnight at 4°C. Transduced $CD34^+$ HSCs were suspended at 2×10^4 cells/mL and 500 μL of cell suspension was added into each pre-coated well. Twice per week, half of the medium from each well was removed and replaced with fresh medium. During this stage, cells would undergo approximately a 300-fold expansion.

At stage 2, cells collected from the stage 1 were cultured in the feeder-free StemSpan SFEM II Medium supplemented with StemSpan Lymphoid Progenitor Maturation Supplement for about 1 week. Non-Treated Falcon Polystyrene 6-well Microplates (Thermo Fisher Scientific) were coated with 1 mL/well of StemSpan Lymphoid Differentiation Coating Material. The stage 1 cells were collected and resuspended at 1×10^5 cells/mL; 2 mL of cell suspension was added into each pre-coated well. Cells were passaged 2–3 times per week to maintain a cell density at $1–2 \times 10^6$ cells per well; fresh medium was added at every passage. During this stage, cells would undergo approximately a 10-fold expansion.

At stage 3, cells collected from stage 2 were cultured in the feeder-free StemSpan SFEM II Medium supplemented with StemSpan Lymphoid Progenitor Maturation Supplement, CD3/CD28/CD2 T Cell Activator and 20 ng/mL human recombinant IL-15 for ~1 week. Cells were resuspended at 5×10^5 cells/mL; 2 mL cell suspension was added into Non-Treated Falcon Polystyrene 6-well Microplates (Thermo Fisher Scientific) pre-coated with 1 mL/well of StemSpan Lymphoid Differentiation Coating Material. Cells were passaged 2–3 times per week to maintain a cell density at $1–2 \times 10^6$ cells per well, and fresh medium was added at every passage. During this stage, cells would undergo approximately a 15-fold expansion.

At stage 4, cells collected from stage 3, now mature $^{Allo/U15}$BCAR-NKT cells or their derivatives, were expanded using various expansion approaches: (1) an αCD3/αCD28 expansion approach, (2) an αGC/PBMC expansion approach, or (3) an aAPC expansion approach. The expansion stage lasted for ~1–2 weeks. The expansion can happen in a feeder-free, serum-free CTS OpTmizer T Cell Expansion SFM (Thermo Fisher Scientific), or a homemade C10 medium. The resulting $^{Allo/U15}$BCAR-NKT cells were aliquoted and cryopreserved in CryoStor Cell Cryopreservation Medium CS10 using a Thermo Scientific CryoMed Controlled-Rate Freezer 7450 (Thermo Scientific) for future use, following the manufacturer's instructions. During this stage, cells would undergo approximately a 150-fold expansion.

The αCD3/αCD28 antibody expansion approach

CELLSTAR 24-well Cell Culture Nontreated Multiwell Plates (VWR) were coated with 1 μg/mL (500 μL/well) of Ultra-LEAF Purified Anti-Human CD3 Antibody (clone OKT3, BioLegend) for 2 h at room temperature or overnight at 4°C. Mature $^{Allo/U15}$BCAR-NKT cells collected from the stage 3 culture were resuspended in the expansion medium supplemented with 10 ng/mL IL-7, 10 ng/mL IL-15, and 1 μg/mL Ultra-LEAF Purified Anti-Human CD28 antibody (clone CD28.2, BioLegend) at 5×10^5 cells/mL; 2 mL cell suspension was added into each pre-coated well. After 3 days, cells were collected and resuspended in fresh expansion medium supplemented with 10 ng/mL IL-7 and IL-15, at $0.5–1 \times 10^6$ cells/mL; 2 mL cell suspension was added into each well of Corning Costar Flat Bottom Cell Culture 6-well Plates (Corning, no αCD3 antibody coating). Cells were passaged 2–3 times per week to maintain a cell density at $0.5–1 \times 10^6$ cells/mL; fresh medium was added at every passage.

The αGC/PBMC expansion approach

Healthy donor PBMCs were loaded with αGC (Avanti Polar Lipids) at 5 μg/mL in C10 medium for 1 h following a previously established protocol.[25] The resulting αGC-loaded PBMCs (αGC/PBMCs) were then irradiated at 6,000 rads using a Rad Source RS-2000 X-Ray Irradiator (Rad Source Technologies). Mature $^{Allo/U15}$BCAR-NKT cells and derivatives collected from the stage 3 culture were mixed with the irradiated αGC/PBMCs at 1:5 ratio, resuspended in expansion medium supplemented with 10 ng/mL IL-7 and IL-15 at $0.5–1 \times 10^6$ cells/mL, and seeded into the Corning Costar Flat Bottom Cell Culture 6-well Plates at 2 mL per well. Cells were passaged 2–3 times per week to maintain a cell density at $0.5–1 \times 10^6$ cells/mL; fresh medium was added at every passage.

The aAPC expansion approach

aAPCs were irradiated at 10,000 rads using a Rad Source RS-2000 X-Ray Irradiator (Rad Source Technologies). Mature $^{Allo/U15}$BCAR-NKT cells collected from the stage 3 culture were mixed with the irradiated aAPCs at 1:1 ratio, resuspended in expansion medium supplemented with 10 ng/mL IL-7 and IL-15 at $0.5–1 \times 10^6$ cells/mL, and seeded into the Corning Costar Flat Bottom Cell Culture 6-well Plates (Corning) at 2 mL per well. $^{Allo/U15}$BCAR-NKT cells were passaged 2–3 times per week to maintain a cell density at $0.5–1 \times 10^6$ cells/mL; fresh medium was added at every passage.

Generation of PBMC-derived conventional αβ T and NK cells

Healthy donor PBMCs were used to generate the PBMC-derived conventional αβ T and NK cells (denoted as PBMC-T and PBMC-NK cells, respectively). To generate PBMC-T cells, PBMCs were stimulated with Dynabeads Human T-Activator CD3/CD28 (Thermo Fisher Scientific) according to the manufacturer's instructions, followed by culturing in the C10 medium supplemented with 20 ng/mL IL-2 for 2–3 weeks. To generate PBMC-NK cells, PBMCs were FACS sorted using a FACSAria III Sorter (BD Biosciences) via human CD56 antibody (clone HCD56, BioLegend) labeling, or MACS sorted using a Human NK Cell Isolation Kit (Miltenyi Biotech), following the manufacturer's instructions.

Generation of BCAR-T cells

Non-treated tissue culture 24-well plates (Corning) were coated with Ultra-LEAF Purified Anti-Human CD3 Antibody (clone OKT3, BioLegend) at 1 μg/mL (500 μL/well), at room temperature for 2 h or at 4°C overnight. Healthy donor PBMCs were resuspended in the C10 medium supplemented with 1 μg/mL Ultra-LEAF Purified Anti-Human CD28 Antibody (clone CD28.2, BioLegend) and 30 ng/mL IL-2, followed by seeding in the pre-coated plates at 1×10^6 cells/mL (1 mL/well). On day 2, cells were transduced with Lenti/BCAR viruses for 24 h. The resulting BCAR-T cells were expanded for about 2 weeks in C10 medium and cryopreserved for future use, following established protocols.[21]

Generation of BCAR-NK cells

Healthy donor PBMCs were MACS sorted using a Human NK Cell Isolation Kit (Miltenyi Biotech), following the manufacturer's instructions. The enriched NK cells were mixed with irradiated aAPCs at a ratio of 1:10, followed by culturing in C10 medium supplemented with 10 ng/mL IL-7 and IL-15. On day 3, NK cells were transduced with Lenti/BCAR viruses for 24 h. The resulting BCAR-NK cells were expanded for about 1 week in C10 medium supplemented with 10 ng/mL IL-7 and IL-15 and cryopreserved for future use.

Generation of ^{PBMC}NKT cells

To generate ^{PBMC}NKT cells, PBMCs were MACS sorted via Anti-iNKT Microbeads (Miltenyi Biotech) labeling to enrich NKT cells, following the manufacturer's instructions. The enriched NKT cells were mixed with donor-matched irradiated αGC/PBMCs at a ratio of 1:1, followed by culturing in C10 medium supplemented with 10 ng/mL IL-7 and IL-15 for 2–3 weeks. If needed, the resulting cultured cells could be further purified using FACS via human NKT TCR antibody (clone 6B11, BD Biosciences) staining.

Generation of PBMC-derived IL-15-enhanced BCAR-engineered NKT (^{PBMC15}BCAR-NKT) cells

Healthy donor PBMCs were MACS-sorted via Anti-iNKT Microbeads (Miltenyi Biotech) labeling to enrich NKT cells, following the manufacturer's instructions. The enriched NKT cells were mixed with donor-matched irradiated αGC/PBMCs at a ratio of 1:1, followed by culturing in C10 medium supplemented with 10 ng/mL IL-7 and IL-15. On day 3, NKT cells were transduced with Lenti/BCAR-IL15 viruses for 24 h. The resulting ^{PBMC15}BCAR-NKT cells were expanded for about 2 weeks in C10 medium supplemented with 10 ng/mL IL-7 and IL-15 and cryopreserved for future use.

Generation of healthy donor PBMC-derived M1- and M2-polarized macrophages and MDSCs

Human monocytes were isolated from PBMCs by adherence. In brief, PBMCs were suspended in serum-free RPMI 1640 media (Corning Cellgro) at 1×10^7 cells/mL. About 10–15 mL of the cell suspension was added to each 10 cm dish and incubated for 1 h. Next, medium containing non-adherent cells was discarded. The dishes were then washed twice using PBS, and the adherent monocytes were used to generate M1- or M2-polarized macrophages or MDSCs. To generate M1-polarized macrophages, monocytes were cultured in C10 medium supplemented with human GM-CSF (10 ng/mL) for 6 days to generate MDMs. At day 6, the generated MDMs were dissociated by 0.25% Trypsin/EDTA (Gibco), collected, and reseeded in 6- or 12-well plates in C10 medium (0.5–1 $\times 10^6$ cells/mL) for 48 h in the presence of recombinant human IFN-γ (20 ng/mL) and LPS (50 ng/mL) to induce M1-polarized macrophage polarization. To generate M2-polarized macrophages, monocytes were cultured in C10 medium supplemented with human M-CSF (10 ng/mL) for 6 days to generate MDMs. At day 6, the generated MDMs were dissociated by 0.25% Trypsin/EDTA (Gibco), collected, and reseeded in 6- or 12-well plates in C10 medium (0.5–1 $\times 10^6$ cells/mL) for 48 h in the presence of recombinant human IL-4 (10 ng/mL) and human IL-13 (10 ng/mL) to induce M2-polarized macrophage polarization. To generate MDSCs, monocytes were cultured in C10 medium supplemented with human GM-CSF and IL-6 (10 ng/mL) for 6 days.

BCAR-NKT cell phenotype and functional study

BCAR-NKT cells were analyzed in comparison with BCAR-T cells. The phenotype of these cells was studied using flow cytometry, by analyzing cell surface markers including co-receptors (i.e., CD4 and CD8), NK cell receptors (e.g., CD161, NKG2D, DNAM-1, NKp30, NKp44, and NKp46), and memory T cell markers (i.e., CD45RO). The capacity of these cells to produce cytokines (i.e., IFN-γ, TNF-α, and IL-2) and cytotoxic molecules (i.e., Perforin and Granzyme B) were studied using flow cytometry via intracellular staining.

Response of BCAR-NKT cells to antigen stimulation was studied by culturing BCAR-NKT cells *in vitro* in C10 medium for 7 days, in the presence or absence of αGC (100 ng/mL). Proliferation of BCAR-NKT cells was measured by cell counting and flow cytometry (identified as $6B11^+CD3^+$) over time. Cytokine production was assessed by ELISA analysis of cell culture supernatants collected on day 7 (for human IFN-γ, TNF-α, IL-2, IL-4, and IL-17).

In vitro tumor cell killing assay

Tumor cells (1×10^4 cells per well) were co-cultured with therapeutic cells (at ratios indicated in the figure legends) in Corning 96-well clear bottom black plates for 24 h, in C10 medium with or without the addition of αGC (100 ng/mL). At the end of culture, live tumor cells were quantified by adding D-luciferin (150 μg/mL, Caliper Life Science) to cell cultures and reading out luciferase activities using an Infinite M1000 microplate reader (Tecan).

In tumor killing assays involving blocking CD1d, 10 μg/mL LEAF purified anti-human CD1d antibody (clone 51.1, BioLegend) or LEAF purified mouse lgG2bk isotype control antibody (clone MG2B-57, BioLegend) was added to tumor cell cultures 1 h prior to adding ^{Allo}(CAR)-NKT cells. In some experiments, 10 μg/mL LEAF purified anti-human NKG2D (clone 1D11, BioLegend), anti-human DNAM-1 antibody (clone 11A8, BioLegend), or LEAF purified mouse lgG2bk isotype control antibody (clone MG2B-57, BioLegend) was added to co-cultures, to study the NKR-mediated tumor cell killing mechanism.

In vitro serial tumor cell killing assay

A total of 1×10^4 non-engineered tumor cells (e.g., MM.1S cells; referred to as stimulator cells) was co-cultured with 2×10^5 therapeutic cells in a Corning 96-well clear bottom black plate in C10 medium. Cultures were supplemented with a dose of 1×10^4 stimulator cells every 2 days. Stimulator cells were then substituted with 1×10^4 of FG-engineered tumor cells (e.g., MM.1S-FG cells; referred to as indicator cells) 24 h prior to luminescent readout of tumor killing. On the day of imaging, remaining live indicator cells were quantified through addition of 100 μL of D-Luciferin (10 mg/mL) with subsequent readout using an Infinite M1000 microplate reader (Tecan) to measure luciferase activity from residual indicator cells.

In vitro assays using MM patient samples

Primary MM patient BM samples were collected and subsequently diluted in PBS and subjected to density gradient centrifugation using Ficoll-Paque (Thermo Fisher Scientific) to obtain mononuclear cells following the manufacturer's instructions. The resulting cells were cryopreserved for future use.

In one assay, the primary MM patient samples were analyzed for tumor cell phenotype and the TME composition using flow cytometry. Tumor cells were identified as $CD45^-CD31^-$FAP (fibroblast activation protein)$^-CD38^+CD138^+$ cells, T cells were identified as $CD45^+$ $CD3^+$ cells, B cells were identified as $CD45^+CD19^+$ cells, NK cells were identified as $CD45^+CD56^+$ cells, monocytes and macrophages were identified as $CD45^+CD11b^+CD14^+$ cells, granulocytes were identified as $CD45^+CD11b^+CD14^-$ cells, and granulocytic MDSCsS were identified as $CD45^+CD11b^+CD14^-HLA\text{-}DR^+$ cells. Surface expression of BCMA, CD1d, and NK ligands on tumor or/and immune cells were also analyzed using flow cytometry.

In another assay, the primary MM patient samples were used to study tumor cell killing by $^{Allo/U15}$BCAR-NKT cells. Tumor cells were sorted using a Human Tumor Cell Isolation Kit (Miltenyi Biotec), followed by co-culturing with various therapeutic cells (E:T ratio = 1:1) in C10 medium in Corning 96-well Round Bottom Cell Culture plates for 24 h. At the end of culture, cells were collected and live MM tumor cells (identified as $CD45^-CD3^-6B11^-$) were analyzed using flow cytometry.

In another assay, the primary MM patient samples were used to study the TME targeting by $^{Allo/U15}$BCAR-NKT cells. Patient samples were directly co-cultured with $^{Allo/U15}$BCAR-NKT cells (ratio 1:1) in C10 medium in Corning 96-well Round Bottom Cell Culture plates for 24 h. At the end of culture, cells were collected, and the TME targeting of ^{Allo15}BCAR-NKT cells was assessed using flow cytometry by quantifying live human TAMs (identified as $6B11^-CD45^{hi}$ $CD14^+CD11b^+$), MDSCs (identified as $6B11^-CD45^{med}CD11b^+$ $CD14^-HLA\text{-}DR^+$), CD4 T cells (identified as $6B11^-CD3^+CD4^+$), CD8 T cells (identified as $6B11^-CD3^+CD8^+$), B cells (identified as $6B11^-CD3^-CD19^+$), granulocytes (identified as $6B11^-CD45^{med}$ $CD11b^+CD14^-HLA\text{-}DR^-$), and NK cells (identified as $6B11^-$ $CD3^-CD56^+$).

In vitro macrophage/MDSC targeting assay

Macrophages or MDSCs (1×10^5 cells per well) were co-cultured with therapeutic cells (1×10^5 cells per well) in 96-well round bottom plates for 24 h, in C10 medium with or without the addition of αGC (100 ng/mL). At the end of culture, live macrophages or MDSCs (identified as $CD11b^+CD14^+$ cells) were quantified using flow cytometry. In assays involving blocking CD1d, 10 μg/mL LEAF purified anti-human CD1d antibody (clone 51.1, BioLegend) or LEAF purified mouse lgG2bk isotype control antibody (clone MG2B-57, BioLegend) was added to cell cultures 1 h prior to adding BCAR-NKT cells.

In vitro MLR assay: Studying GvH response

PBMCs from multiple random healthy donors were irradiated at 2,500 rads and used as stimulators to study the GvH response of $^{Allo/U15}$BCAR-NKT cells as responders. PBMC-derived BCAR-T cells were included as a responder control. Stimulators (5×10^5 cells/well) and responders (2×10^4 cells/well) were co-cultured in 96-well round-bottom plates in C10 medium for 4 days; the cell culture supernatants were then collected to measure IFN-γ production using ELISA. Note that the IFN-γ production is solely attributed to $^{Allo/U15}$BCAR-NKT or BCAR-T responder cells.

In vitro MLR assay: Studying T cell-mediated allorejection

PBMCs from multiple healthy donors were used as responders to study the T cell-mediated allorejection of $^{Allo/U15}$BCAR-NKT cells as stimulators, which were irradiated at 2,500 rads. PBMC-derived BCAR-T cells were included as a stimulator control. Irradiated stimulators (5×10^5 cells/well) and responders (2×10^4 cells/well) were co-cultured in 96-well round-bottom plates in C10 medium for 4 days; the cell culture supernatants were then collected to measure IFN-γ production using ELISA. Note that the IFN-γ production is solely attributed to PBMC responder cells.

In vitro MLR assay: Studying NK cell-mediated allorejection

PBMC-derived NK cells obtained from multiple healthy donors were employed to investigate the NK cell-mediated allorejection of universal BCAR-NKT cells. PBMC-derived BCAR-T cells were included as an allogeneic subject control. PBMC-NK cells (2×10^4 cells/well) and the corresponding allogeneic subject cells (2×10^4 cells/well) were co-cultured in 96-well round bottom plates with C10 medium for 24 h. Subsequently, the cell cultures were collected to quantify live cells via flow cytometry.

In vitro SEM

SEM was used to visualize the ^{Allo15}BCAR-NKT cell targeting of MM.1S-FG human MM cells. The ^{Allo15}BCAR-NKT cells were co-cultured with MM.1S -FG cells in C10 medium on a Cover Glass Slide (Bioland Scientific) for 8 h. After the culture, the cells were rinsed with warm HBSS and then fixed with warm 3% glutaraldehyde in the SEM buffer, moved to 4°C, and stored overnight. The SEM buffer (pH 7.4) was prepared using 0.1 M Na-phosphate buffer containing 0.1 M sucrose. On the second day, the cells were washed twice with SEM buffer for 5 min each time. Then, the cells were fixed with 2% osmium tetroxide in SEM buffer on ice for 1 h and washed again with SEM

buffer twice for 5 min each time. After the washing step, the cells were dehydrated successively with 50%, 70%, 95%, and 100% ethanol, each for 15 min. The final 100% ethanol was replaced with hexamethyldisilazane, and then the cells were evaporated in the hood. The processed cells were then subjected to low-vacuum SEM on an FEI Nova Nano 230 SEM (Thermo Fisher Scientific), following the manufacturer's instructions.

Immunostaining and confocal microscopy

$^{Allo/U15}$BCAR-NKT and BCAR-T cells were fixed using 4% PFA. Following fixation, the cells were washed with PBS and blocked with 10% goat serum (Thermo Fisher Scientific). Subsequently, they were stained using the following antibodies: FITC anti-human CD3 Antibody (OKT3, BioLegend, 1:20), APC anti-human β2-microglobulin Antibody (A17082A, BioLegend, 1:100), and PE/Cyanine7 anti-human HLA-DR Antibody (Tü39, BioLegend, 1:10), all in 10% goat serum. After staining, the cells were washed with PBS, placed onto glass slides, and sealed using Prolong Gold antifade reagent with DAPI (Thermo Fisher Scientific). For imaging, a Leica Confocal SP8-STED/FLIM/FCS microscope was employed. The acquired images were subsequently processed using DeconvolutionLab2 in ImageJ.

In vivo BLI

BLI was performed using a spectral advanced molecular maging HTX imaging system (Spectral Instrument Imaging). Live animal images were acquired 5 min after intraperitoneal (i.p.) injection of D-Luciferin (1 mg/mouse for visualizing tumor cells, and 3 mg/mouse for visualizing therapeutic cells) for total body bioluminescence. Imaging data were analyzed using an AURA imaging software (Spectral Instrument Imaging, version 3.2.0).

In vivo PK/PD and T cell-mediated allorejection study of universal BCAR-NKT cells

The experimental design is shown in Figure 3F. In brief, on day −4, NSG mice received intravenous (i.v.) injection of healthy donor-derived PBMCs (1×10^7 cells per mouse). In one experiment set, PBMCs from a single donor were utilized to compare allorejection with different therapeutic cells. These PBMCs are donor-mismatched with the therapeutic cells. On day 0, the experimental mice received i.v. injection of $^{Allo/U15}$BCAR-NKT/FG cells (10×10^6 cells in 100 μL PBS per mouse), or control BCAR-T/FG cells (10×10^6 cells in 100 μL PBS per mouse). Over the experiment, mice were monitored for survival and their therapeutic cells were measured twice per week using BLI. Note, in this study, therapeutic cells but not the tumor cells were labeled with FG.

In vivo antitumor efficacy study of universal BCAR-NKT cells: Human MM xenograft NSG mouse model

The experimental design is shown in Figures 5A, 5E, 5I, and 5M. In brief, on day 0, NSG mice received i.v. inoculation of human MM cells, including MM.1S-FG, MM.1S-CD1d-FG, and ^{KO}MM.1S-FG cells (1×10^6 cells per mouse). On days 4 or 20, the experimental mice received i.v. injection of vehicle (100 μL PBS per mouse), $^{Allo/U15}$BCAR-NKT cells (10×10^6 CAR$^+$ cells in 100 μL PBS per mouse), or control BCAR-T cells (10×10^6 CAR$^+$ cells in 100 μL PBS per mouse). Over the experiment, mice were monitored for survival and their tumor loads were measured twice per week using BLI.

In vivo antitumor efficacy study of universal BCAR-NKT cells under T cell-mediated allorejection: Human MM xenograft NSG mouse model

The experimental design is shown in Figure 6A. In brief, on day 0, NSG mice received i.v. inoculation of human MM.1S-FG cells (1×10^6 cells per mouse). On day 4, the experimental mice received i.v. injection of healthy donor-derived donor-mismatched PBMCs (1×10^7 cells per mouse). On day 8, the experimental mice received i.v. injection of vehicle (100 μL PBS per mouse), $^{Allo/U15}$BCAR-NKT cells (10×10^6 CAR$^+$ cells in 100 μL PBS per mouse), or control BCAR-T cells (10×10^6 CAR$^+$ cells in 100 μL PBS per mouse). Over the experiment, mice were monitored for survival and their tumor loads were measured twice per week using BLI.

In vivo antitumor efficacy study of universal BCAR-NKT cells: Human ovarian cancer xenograft NSG mouse model

The experimental design is shown in Figure S5A. In brief, on day 0, NSG mice received i.p. inoculation of OVCAR8-FG human ovarian cancer cells (5×10^5 cells per mouse). On day 4, the experimental mice received i.v. injection of vehicle (100 μL PBS per mouse), ^{U15}BCAR-NKT cells, or control PBMC-NK cells. Over the experiment, mice were monitored for survival and their tumor loads were measured 3 times per week using BLI.

In vivo CRS study

The experimental design is shown in Figure 8E. In brief, on day 0, NSG mice received i.v. inoculation of MM.1S-FG cells (5×10^6 cells per mouse). On day 10, the experimental mice received i.v. injection of vehicle (100 μL PBS per mouse), $^{Allo/U15}$BCAR-NKT cells (10×10^6 CAR$^+$ cells in 100 μL PBS per mouse), or control BCAR-T cells (10×10^6 CAR$^+$ cells in 100 μL PBS per mouse). On days 11 and 13, blood samples were collected from the experimental mice, and their serum IL-6 and SAA-3 were measured using ELISA. A Mouse SAA-3 ELISA Kit (Millipore Sigma) was used to measure SAA-3, following the manufacturer's instructions.

In vivo GCV depletion study

The experimental design is shown in Figure S9C. In brief, on day 0, NSG mice received i.v. injection of ^{U}BCAR-NKT-TK cells, followed by i.p. injection of GCV for 5 consecutive days (50 mg/kg per injection per day). On day 5, mice were terminated. Multiple tissues (i.e., blood, spleen, liver, and lung) were collected and processed for flow cytometry analysis to detect circulating and tissue-infiltrating ^{U}BCAR-NKT-TK cells (identified as iNKT TCR$^+$CD45$^+$).

Histology analysis

Tissues (i.e., liver and lung) were collected from experimental mice, fixed in 10% neutral buffered formalin for up to 36 h, and embedded in paraffin for sectioning (5 μm thickness). Tissue sections were

prepared and stained with hematoxylin and eosin by the UCLA Translational Pathology Core Laboratory (TPCL), following the Core's standard protocols. Stained sections were imaged using an Olympus BX51 upright microscope equipped with an Optronics Macrofire CCD camera (AU Optronics). The images were analyzed using an Optronics PictureFrame software (AU Optronics).

Statistics

GraphPad Prism 8 software (GraphPad) was used for statistical data analysis. Student's two-tailed t test was used for pairwise comparisons. Ordinary one- or two-way ANOVA followed by Tukey's or Dunnett's multiple comparisons test was used for multiple comparisons. Log rank (Mantel-Cox) test adjusted for multiple comparisons was used for Meier survival curves analysis. Data are presented as the mean ± SEM, unless otherwise indicated. In all figures and figure legends, n represents the number of samples or animals utilized in the indicated experiments. A p value of less than 0.05 was considered significant. ns, not significant; $*p < 0.05$, $**p < 0.01$, $***p < 0.001$, $****p < 0.0001$.

DATA AND CODE AVAILABILITY

All data associated with this study are present in the paper or supplemental information.

SUPPLEMENTAL INFORMATION

Supplemental information can be found online at https://doi.org/10.1016/j.ymthe.2024.04.005.

ACKNOWLEDGMENTS

We thank the University of California, Los Angeles (UCLA) animal facility for providing animal support, the UCLA Translational Pathology Core Laboratory (TPCL) for providing histology support, the UCLA CFAR Virology Core for providing human cells, the UCLA BSCRC Flow Cytometry Core Facility for cell sorting support, and the UCLA Advanced Light Microscopy/Spectroscopy Technology Center for confocal microscopy support. This work was supported by a Partnering Opportunity for Discovery Stage Research Projects Award and a Partnering Opportunity for Translational Research Projects Award from the California Institute for Regenerative Medicine (DISC2-11157 and TRAN1-12250, to L.Y.), a Department of Defense CDMRP PRCRP Impact Award (CA200456 to L.Y.), a UCLA BSCRC Innovation Award (to L.Y.), and an Ablon Scholars Award (to L.Y.). Y.-R.L. is a postdoctoral fellow supported by a UCLA MIMG M. John Pickett Post-Doctoral Fellow Award and a CIRM-BSCRC Postdoctoral Fellowship. D.L. and Z.L. are postdoctoral fellows supported by T32 Tumor Immunology Training Grant postdoctoral fellowships (USHHS Ruth L. Kirschstein Institutional National Research Service Award, T32-CA009120).

AUTHOR CONTRIBUTIONS

Y.-R.L. and L.Y. designed the experiments, analyzed the data, and wrote the manuscript. L.Y. conceived and oversaw the study, with assistance from Y.-R.L. and suggestions from P.W. and T.H. Y.-R.L. performed all experiments, with assistance from Y. Zhou, J.Y., Y. Zhu, D.L., E.Z., Z.L., Y.J.K., K.Z., Y.F., Z.L., Y.C., Y.T., J.H., X.C., T.H., and J.M.C. J.J.Z. helped with the statistical analysis of data. B.R.P. and S.M.L. provided and assisted on the analysis of primary MM patient BM samples.

DECLARATION OF INTERESTS

Y.-R.L., Y. Zhou, J.Y., D.L., Z.L., Y.J.K., P.W., and L.Y. are inventors on patents relating to this study filed by UCLA. Y. Zhou is currently an employee of Amberstone Biosciences. J.Y. is currently an employee of Appia Bio. Z.L. is currently an employee of Allogene. Y.J.K. is currently an employee of Nkarta. P.W. is a co-founder, stockholder, and advisory board member of Simnova Bio, TCRCure Biopharma, Appia Bio, and is a scientific advisor to Grit Biotechnolgoy. L.Y. is a scientific advisor to AlzChem and Amberstone Biosciences, and a co-founder, stockholder, and advisory board member of Appia Bio. Appia Bio licensed some patents relating to this study from UCLA. None of the declared companies contributed to or directed any of the research reported in this article. The remaining authors declare no competing interests.

REFERENCES

1. June, C.H., O'Connor, R.S., Kawalekar, O.U., Ghassemi, S., and Milone, M.C. (2018). CAR T cell immunotherapy for human cancer. Science *359*, 1361–1365. https://doi.org/10.1126/science.aar6711.
2. Sterner, R.C., and Sterner, R.M. (2021). CAR-T cell therapy: current limitations and potential strategies. Blood Cancer J. *11*, 69. https://doi.org/10.1038/s41408-021-00459-7.
3. Labanieh, L., and Mackall, C.L. (2023). CAR immune cells: design principles, resistance and the next generation. Nature *614*, 635–648. https://doi.org/10.1038/s41586-023-05707-3.
4. Finck, A.V., Blanchard, T., Roselle, C.P., Golinelli, G., and June, C.H. (2022). Engineered cellular immunotherapies in cancer and beyond. Nat. Med. *28*, 678–689. https://doi.org/10.1038/s41591-022-01765-8.
5. Li, Y.-R., Dunn, Z.S., Yu, Y., Li, M., Wang, P., and Yang, L. (2023). Advancing cell-based cancer immunotherapy through stem cell engineering. Cell Stem Cell *30*, 592–610. https://doi.org/10.1016/j.stem.2023.02.009.
6. Newick, K., O'Brien, S., Moon, E., and Albelda, S.M. (2017). CAR T Cell Therapy for Solid Tumors. Annu. Rev. Med. *68*, 139–152. https://doi.org/10.1146/annurev-med-062315-120245.
7. Baker, D.J., Arany, Z., Baur, J.A., Epstein, J.A., and June, C.H. (2023). CAR T therapy beyond cancer: the evolution of a living drug. Nature *619*, 707–715. https://doi.org/10.1038/s41586-023-06243-w.
8. Benjamin, R., Graham, C., Yallop, D., Jozwik, A., Mirci-Danicar, O.C., Lucchini, G., Pinner, D., Jain, N., Kantarjian, H., Boissel, N., et al. (2020). Genome-edited, donor-derived allogeneic anti-CD19 chimeric antigen receptor T cells in paediatric and adult B-cell acute lymphoblastic leukaemia: results of two phase 1 studies. Lancet *396*, 1885–1894. https://doi.org/10.1016/S0140-6736(20)32334-5.
9. van der Stegen, S.J.C., Lindenbergh, P.L., Petrovic, R.M., Xie, H., Diop, M.P., Alexeeva, V., Shi, Y., Mansilla-Soto, J., Hamieh, M., Eyquem, J., et al. (2022). Generation of T-cell-receptor-negative CD8αβ-positive CAR T cells from T-cell-derived induced pluripotent stem cells. Nat. Biomed. Eng. *6*, 1284–1297. https://doi.org/10.1038/s41551-022-00915-0.
10. Nair, S., and Dhodapkar, M.V. (2017). Natural Killer T Cells in Cancer Immunotherapy. Front. Immunol. *8*, 1178. https://doi.org/10.3389/fimmu.2017.01178.
11. Vivier, E., Ugolini, S., Blaise, D., Chabannon, C., and Brossay, L. (2012). Targeting natural killer cells and natural killer T cells in cancer. Nat. Rev. Immunol. *12*, 239–252. https://doi.org/10.1038/nri3174.

12. Fujii, S.I., Shimizu, K., Okamoto, Y., Kunii, N., Nakayama, T., Motohashi, S., and Taniguchi, M. (2013). NKT cells as an ideal anti-tumor immunotherapeutic. Front. Immunol. *4*, 409. https://doi.org/10.3389/fimmu.2013.00409.

13. Heczey, A., Courtney, A.N., Montalbano, A., Robinson, S., Liu, K., Li, M., Ghatwai, N., Dakhova, O., Liu, B., Raveh-Sadka, T., et al. (2020). Anti-GD2 CAR-NKT cells in patients with relapsed or refractory neuroblastoma: an interim analysis. Nat. Med. *26*, 1686–1690. https://doi.org/10.1038/s41591-020-1074-2.

14. Brennan, P.J., Brigl, M., and Brenner, M.B. (2013). Invariant natural killer T cells: an innate activation scheme linked to diverse effector functions. Nat. Rev. Immunol. *13*, 101–117. https://doi.org/10.1038/nri3369.

15. Heczey, A., Xu, X., Courtney, A.N., Tian, G., Barragan, G.A., Guo, L., Amador, C.M., Ghatwai, N., Rathi, P., Wood, M.S., et al. (2023). Anti-GD2 CAR-NKT cells in relapsed or refractory neuroblastoma: updated phase 1 trial interim results. Nat. Med. *29*, 1379–1388. https://doi.org/10.1038/s41591-023-02363-y.

16. Courtney, A.N., Tian, G., and Metelitsa, L.S. (2023). Natural killer T cells and other innate-like T lymphocytes as emerging platforms for allogeneic cancer cell therapy. Blood *141*, 869–876. https://doi.org/10.1182/blood.2022016201.

17. Bendelac, A., Savage, P.B., and Teyton, L. (2007). The biology of NKT cells. Annu. Rev. Immunol. *25*, 297–336. https://doi.org/10.1146/annurev.immunol.25.022106.141711.

18. Wagner, D.L., Fritsche, E., Pulsipher, M.A., Ahmed, N., Hamieh, M., Hegde, M., Ruella, M., Savoldo, B., Shah, N.N., Turtle, C.J., et al. (2021). Immunogenicity of CAR T cells in cancer therapy. Nat. Rev. Clin. Oncol. *18*, 379–393. https://doi.org/10.1038/s41571-021-00476-2.

19. Li, Y.-R., Zhou, K., Wilson, M., Kramer, A., Zhu, Y., Dawson, N., and Yang, L. (2023). Mucosal-associated invariant T cells for cancer immunotherapy. Mol. Ther. *31*, 631–646. https://doi.org/10.1016/j.ymthe.2022.11.019.

20. Themeli, M., Rivière, I., and Sadelain, M. (2015). New cell sources for T cell engineering and adoptive immunotherapy. Cell Stem Cell *16*, 357–366. https://doi.org/10.1016/j.stem.2015.03.011.

21. Li, Y.-R., Zhou, Y., Kim, Y.J., Zhu, Y., Ma, F., Yu, J., Wang, Y.-C., Chen, X., Li, Z., Zeng, S., et al. (2021). Development of allogeneic HSC-engineered iNKT cells for off-the-shelf cancer immunotherapy. Cell Rep. Med. *2*, 100449. https://doi.org/10.1016/j.xcrm.2021.100449.

22. Abrahimi, P., Chang, W.G., Kluger, M.S., Qyang, Y., Tellides, G., Saltzman, W.M., and Pober, J.S. (2015). Efficient gene disruption in cultured primary human endothelial cells by CRISPR/Cas9. Circ. Res. *117*, 121–128. https://doi.org/10.1161/CIRCRESAHA.117.306290.

23. Ren, J., Liu, X., Fang, C., Jiang, S., June, C.H., and Zhao, Y. (2017). Multiplex genome editing to generate universal CAR T cells resistant to PD1 inhibition. Clin. Cancer Res. *23*, 2255–2266. https://doi.org/10.1158/1078-0432.CCR-16-1300.

24. Steimle, V., Siegrist, C.A., Mottet, A., Lisowska-Grospierre, B., and Mach, B. (1994). Regulation of MHC class II expression by interferon-γ mediated by the transactivator gene CIITA. Science *265*, 106–109. https://doi.org/10.1126/science.8016643.

25. Zhu, Y., Smith, D.J., Zhou, Y., Li, Y.R., Yu, J., Lee, D., Wang, Y.C., Di Biase, S., Wang, X., Hardoy, C., et al. (2019). Development of Hematopoietic Stem Cell-Engineered Invariant Natural Killer T Cell Therapy for Cancer. Cell Stem Cell *25*, 542–557.e9. https://doi.org/10.1016/j.stem.2019.08.004.

26. Liu, Y., Chen, Z., Fang, H., Wei, R., Yu, K., Jiang, S., Fu, W., Jiang, H., Du, J., He, F., et al. (2018). Durable Remission Achieved from Bcma-Directed CAR-T Therapy Against Relapsed or Refractory Multiple Myeloma. Blood *132*, 956. https://doi.org/10.1182/blood-2018-99-112786.

27. Li, Y.-R., Zhou, Y., Kramer, A., and Yang, L. (2021). Engineering stem cells for cancer immunotherapy. Trends Cancer *7*, 1059–1073. https://doi.org/10.1016/j.trecan.2021.08.004.

28. Godfrey, D.I., and Berzins, S.P. (2007). Control points in NKT-cell development. Nat. Rev. Immunol. *7*, 505–518. https://doi.org/10.1038/nri2116.

29. Lee, P.T., Benlagha, K., Teyton, L., and Bendelac, A. (2002). Distinct functional lineages of human Vα24 natural killer T cells. J. Exp. Med. *195*, 637–641. https://doi.org/10.1084/jem.20011908.

30. Gumperz, J.E., Miyake, S., Yamamura, T., and Brenner, M.B. (2002). Functionally distinct subsets of CD1d-restricted natural killer T cells revealed by CD1d tetramer staining. J. Exp. Med. *195*, 625–636. https://doi.org/10.1084/jem.20011786.

31. Lanza, R., Russell, D.W., and Nagy, A. (2019). Engineering universal cells that evade immune detection. Nat. Rev. Immunol. *19*, 723–733. https://doi.org/10.1038/s41577-019-0200-1.

32. Labanieh, L., Majzner, R.G., and Mackall, C.L. (2018). Programming CAR-T cells to kill cancer. Nat. Biomed. Eng. *2*, 377–391. https://doi.org/10.1038/s41551-018-0235-9.

33. Lam, P.Y., Nissen, M.D., and Mattarollo, S.R. (2017). Invariant natural killer T cells in immune regulation of blood cancers: Harnessing their potential in immunotherapies. Front. Immunol. *8*, 1355. https://doi.org/10.3389/fimmu.2017.01355.

34. Krijgsman, D., Hokland, M., and Kuppen, P.J.K. (2018). The Role of Natural Killer T Cells in Cancer-A Phenotypical and Functional Approach. Front. Immunol. *9*, 367. https://doi.org/10.3389/fimmu.2018.00367.

35. Kronenberg, M. (2005). Toward an understanding of NKT cell biology: progress and paradoxes. Annu. Rev. Immunol. *23*, 877–900. https://doi.org/10.1146/annurev.immunol.23.021704.115742.

36. Li, Y.-R., Zhou, Y., Wilson, M., Kramer, A., Hon, R., Zhu, Y., Fang, Y., and Yang, L. (2022). Tumor-Localized Administration of α-GalCer to Recruit Invariant Natural Killer T Cells and Enhance Their Antitumor Activity against Solid Tumors. Int. J. Mol. Sci. *23*, 7547. https://doi.org/10.3390/ijms23147547.

37. Deuse, T., Hu, X., Gravina, A., Wang, D., Tediashvili, G., De, C., Thayer, W.O., Wahl, A., Garcia, J.V., Reichenspurner, H., et al. (2019). Hypoimmunogenic derivatives of induced pluripotent stem cells evade immune rejection in fully immunocompetent allogeneic recipients. Nat. Biotechnol. *37*, 252–258. https://doi.org/10.1038/s41587-019-0016-3.

38. Figueiredo, C., and Blasczyk, R. (2015). A future with less HLA: potential clinical applications of HLA-universal cells. Tissue Antigens *85*, 443–449. https://doi.org/10.1111/tan.12564.

39. Mullard, A. (2022). FDA approves second BCMA-targeted CAR-T cell therapy. Nat. Rev. Drug Discov. *21*, 249. https://doi.org/10.1038/d41573-022-00048-8.

40. Spanoudakis, E., Hu, M., Naresh, K., Terpos, E., Melo, V., Reid, A., Kotsianidis, I., Abdalla, S., Rahemtulla, A., and Karadimitris, A. (2009). Regulation of multiple myeloma survival and progression by CD1d. Blood *113*, 2498–2507. https://doi.org/10.1182/blood-2008-06-161281.

41. Rafiq, S., Hackett, C.S., and Brentjens, R.J. (2020). Engineering strategies to overcome the current roadblocks in CAR T cell therapy. Nat. Rev. Clin. Oncol. *17*, 147–167. https://doi.org/10.1038/s41571-019-0297-y.

42. Dhatchinamoorthy, K., Colbert, J.D., and Rock, K.L. (2021). Cancer Immune Evasion Through Loss of MHC Class I Antigen Presentation. Front. Immunol. *12*, 636568. https://doi.org/10.3389/fimmu.2021.636568.

43. Zhang, S., Kohli, K., Black, R.G., Yao, L., Spadinger, S.M., He, Q., Pillarisetty, V.G., Cranmer, L.D., Van Tine, B.A., Yee, C., et al. (2019). Systemic Interferon-γ Increases MHC Class I Expression and T-cell Infiltration in Cold Tumors: Results of a Phase 0 Clinical Trial. Cancer Immunol. Res. *7*, 1237–1243. https://doi.org/10.1158/2326-6066.CIR-18-0940.

44. Jorgovanovic, D., Song, M., Wang, L., and Zhang, Y. (2020). Roles of IFN-γ in tumor progression and regression: a review. Biomark. Res. *8*, 49. https://doi.org/10.1186/s40364-020-00228-x.

45. Li, Y.-R., Brown, J., Yu, Y., Lee, D., Zhou, K., Dunn, Z.S., Hon, R., Wilson, M., Kramer, A., Zhu, Y., et al. (2022). Targeting Immunosuppressive Tumor-Associated Macrophages Using Innate T Cells for Enhanced Antitumor Reactivity. Cancers *14*, 2749. https://doi.org/10.3390/cancers14112749.

46. Li, Y.-R., Yu, Y., Kramer, A., Hon, R., Wilson, M., Brown, J., and Yang, L. (2022). An Ex Vivo 3D Tumor Microenvironment-Mimicry Culture to Study TAM Modulation of Cancer Immunotherapy. Cells *11*, 1583. https://doi.org/10.3390/cells11091583.

47. Li, Y.-R., Wilson, M., and Yang, L. (2022). Target tumor microenvironment by innate T cells. Front. Immunol. *13*, 999549. https://doi.org/10.3389/fimmu.2022.999549.

48. Kawano, Y., Moschetta, M., Manier, S., Glavey, S., Görgün, G.T., Roccaro, A.M., Anderson, K.C., and Ghobrial, I.M. (2015). Targeting the bone marrow

microenvironment in multiple myeloma. Immunol. Rev. *263*, 160–172. https://doi.org/10.1111/imr.12233.

49. Neumeister, P., Schulz, E., Pansy, K., Szmyra, M., and Deutsch, A.J. (2022). Targeting the Microenvironment for Treating Multiple Myeloma. Int. J. Mol. Sci. *23*, 7627. https://doi.org/10.3390/ijms23147627.

50. Sun, J., Park, C., Guenthner, N., Gurley, S., Zhang, L., Lubben, B., Adebayo, O., Bash, H., Chen, Y., Maksimos, M., et al. (2022). Tumor-associated macrophages in multiple myeloma: Advances in biology and therapy. J. Immunother. Cancer *10*, e003975. https://doi.org/10.1136/jitc-2021-003975.

51. Li, Y.-R., Ochoa, C.J., Zhu, Y., Kramer, A., Wilson, M., Fang, Y., Chen, Y., Singh, T., Di Bernardo, G., Zhu, E., et al. (2023). Profiling ovarian cancer tumor and microenvironment during disease progression for cell-based immunotherapy design. iScience *26*, 107952. https://doi.org/10.1016/j.isci.2023.107952.

52. Sica, A., Larghi, P., Mancino, A., Rubino, L., Porta, C., Totaro, M.G., Rimoldi, M., Biswas, S.K., Allavena, P., and Mantovani, A. (2008). Macrophage polarization in tumour progression. Semin. Cancer Biol. *18*, 349–355. https://doi.org/10.1016/j.semcancer.2008.03.004.

53. Yunna, C., Mengru, H., Lei, W., and Weidong, C. (2020). Macrophage M1/M2 polarization. Eur. J. Pharmacol. *877*, 173090. https://doi.org/10.1016/j.ejphar.2020.173090.

54. Stegelmeier, A.A., van Vloten, J.P., Mould, R.C., Klafuric, E.M., Minott, J.A., Wootton, S.K., Bridle, B.W., and Karimi, K. (2019). Myeloid Cells during Viral Infections and Inflammation. Viruses *11*, 168. https://doi.org/10.3390/v11020168.

55. Yang, S., Zhao, M., and Jia, S. (2023). Macrophage: Key player in the pathogenesis of autoimmune diseases. Front. Immunol. *14*, 1080310. https://doi.org/10.3389/fimmu.2023.1080310.

56. Winkler, I.G., Sims, N.A., Pettit, A.R., Barbier, V., Nowlan, B., Helwani, F., Poulton, I.J., van Rooijen, N., Alexander, K.A., Raggatt, L.J., and Lévesque, J.P. (2010). Bone marrow macrophages maintain hematopoietic stem cell (HSC) niches and their depletion mobilizes HSCs. Blood *116*, 4815–4828. https://doi.org/10.1182/blood-2009-11-253534.

57. Hill, G.R., Betts, B.C., Tkachev, V., Kean, L.S., and Blazar, B.R. (2021). Current Concepts and Advances in Graft-Versus-Host Disease Immunology. Annu. Rev. Immunol. *39*, 19–49. https://doi.org/10.1146/annurev-immunol-102119-073227.

58. Chang, Y.-J., Zhao, X.-Y., and Huang, X.-J. (2018). Strategies for Enhancing and Preserving Anti-leukemia Effects Without Aggravating Graft-Versus-Host Disease. Front. Immunol. *9*, 3041. https://doi.org/10.3389/fimmu.2018.03041.

59. Tugues, S., Amorim, A., Spath, S., Martin-Blondel, G., Schreiner, B., De Feo, D., Lutz, M., Guscetti, F., Apostolova, P., Haftmann, C., et al. (2018). Graft-versus-host disease, but not graft-versus-leukemia immunity, is mediated by GM-CSF-licensed myeloid cells. Sci. Transl. Med. *10*, eaat8410. https://doi.org/10.1126/scitranslmed.aat8410.

60. Shlomchik, W.D. (2007). Graft-versus-host disease. Nat. Rev. Immunol. *7*, 340–352. https://doi.org/10.1038/nri2000.

61. Chen, Y., Zhu, Y., Kramer, A., Fang, Y., Wilson, M., Li, Y.-R., and Yang, L. (2023). Genetic engineering strategies to enhance antitumor reactivity and reduce alloreactivity for allogeneic cell-based cancer therapy. Front. Med. *10*, 1135468. https://doi.org/10.3389/fmed.2023.1135468.

62. Fang, Y., Zhu, Y., Kramer, A., Chen, Y., Li, Y.-R., and Yang, L. (2023). Graft-versus-Host Disease Modulation by Innate T Cells. Int. J. Mol. Sci. *24*, 4084. https://doi.org/10.3390/ijms24044084.

63. Pillai, A.B., George, T.I., Dutt, S., Teo, P., and Strober, S. (2007). Host NKT Cells Can Prevent Graft-versus-Host Disease and Permit Graft Antitumor Activity after Bone Marrow Transplantation. J. Immunol. *178*, 6242–6251. https://doi.org/10.4049/jimmunol.178.10.6242.

64. Giavridis, T., Van Der Stegen, S.J.C., Eyquem, J., Hamieh, M., Piersigilli, A., and Sadelain, M. (2018). CAR T cell-induced cytokine release syndrome is mediated by macrophages and abated by IL-1 blockade letter. Nat. Med. *24*, 731–738. https://doi.org/10.1038/s41591-018-0041-7.

65. Sterner, R.M., Sakemura, R., Cox, M.J., Yang, N., Khadka, R.H., Forsman, C.L., Hansen, M.J., Jin, F., Ayasoufi, K., Hefazi, M., et al. (2019). GM-CSF inhibition reduces cytokine release syndrome and neuroinflammation but enhances CAR-T cell function in xenografts. Blood *133*, 697–709. https://doi.org/10.1182/blood-2018-10-881722.

66. Norelli, M., Camisa, B., Barbiera, G., Falcone, L., Purevdorj, A., Genua, M., Sanvito, F., Ponzoni, M., Doglioni, C., Cristofori, P., et al. (2018). Monocyte-derived IL-1 and IL-6 are differentially required for cytokine-release syndrome and neurotoxicity due to CAR T cells. Nat. Med. *24*, 739–748. https://doi.org/10.1038/s41591-018-0036-4.

67. Li, H., Song, W., Li, Z., and Zhang, M. (2022). Preclinical and clinical studies of CAR-NK-cell therapies for malignancies. Front. Immunol. *13*, 992232. https://doi.org/10.3389/fimmu.2022.992232.

68. Rezvani, K., Rouce, R., Liu, E., and Shpall, E. (2017). Engineering Natural Killer Cells for Cancer Immunotherapy. Mol. Ther. *25*, 1769–1781. https://doi.org/10.1016/j.ymthe.2017.06.012.

69. Klingemann, H. (2014). Are natural killer cells superior CAR drivers? Oncoimmunology *3*, e28147. https://doi.org/10.4161/onci.28147.

70. Gschweng, E.H., McCracken, M.N., Kaufman, M.L., Ho, M., Hollis, R.P., Wang, X., Saini, N., Koya, R.C., Chodon, T., Ribas, A., et al. (2014). HSV-sr39TK positron emission tomography and suicide gene elimination of human hematopoietic stem cells and their progeny in humanized mice. Cancer Res. *74*, 5173–5183. https://doi.org/10.1158/0008-5472.CAN-14-0376.

71. Thomis, D.C., Marktel, S., Bonini, C., Traversari, C., Gilman, M., Bordignon, C., and Clackson, T. (2001). A Fas-based suicide switch in human T cells for the treatment of graft-versus-host disease. Blood *97*, 1249–1257. https://doi.org/10.1182/blood.v97.5.1249.

72. Mashima, H., Zhang, R., Kobayashi, T., Tsukamoto, H., Liu, T., Iwama, T., Hagiya, Y., Yamamoto, M., Fukushima, S., Okada, S., et al. (2021). Improved safety of induced pluripotent stem cell-derived antigen-presenting cell-based cancer immunotherapy. Mol. Ther. Methods Clin. Dev. *21*, 171–179. https://doi.org/10.1016/j.omtm.2021.03.002.

73. Di Stasi, A., Tey, S.-K., Dotti, G., Fujita, Y., Kennedy-Nasser, A., Martinez, C., Straathof, K., Liu, E., Durett, A.G., Grilley, B., et al. (2011). Inducible Apoptosis as a Safety Switch for Adoptive Cell Therapy. N. Engl. J. Med. *365*, 1673–1683. https://doi.org/10.1056/nejmoa1106152.

74. Warda, W., Da Rocha, M.N., Trad, R., Haderbache, R., Salma, Y., Bouquet, L., Roussel, X., Nicod, C., Deschamps, M., and Ferrand, C. (2021). Overcoming target epitope masking resistance that can occur on low-antigen-expresser AML blasts after IL-1RAP chimeric antigen receptor T cell therapy using the inducible caspase 9 suicide gene safety switch. Cancer Gene Ther. *28*, 1365–1375. https://doi.org/10.1038/s41417-020-00284-3.

75. Schaft, N. (2020). The landscape of car-t cell clinical trials against solid tumors—a comprehensive overview. Cancers (Basel) *12*, 2567. https://doi.org/10.3390/cancers12092567.

76. Li, Y.-R., Dunn, Z.S., Zhou, Y., Lee, D., and Yang, L. (2021). Development of Stem Cell-Derived Immune Cells for Off-the-Shelf Cancer Immunotherapies. Cells *10*, 3497. https://doi.org/10.3390/cells10123497.

77. Lee, N., Llano, M., Carretero, M., Geraghty, D.E., Ishitani, A., López-Botet, M., and López-Botet, M. (1998). HLA-E is a major ligand for the natural killer inhibitory receptor CD94/NKG2A. Proc. Natl. Acad. Sci. USA *95*, 5199–5204. https://doi.org/10.1073/pnas.95.9.5199.

78. Benjamin, R., Graham, C., Yallop, D., Jozwik, A., Ciocarlie, O., Jain, N., Jabbour, E.J., Maus, M.V., Frigault, M., Boissel, N., et al. (2018). Preliminary Data on Safety, Cellular Kinetics and Anti-Leukemic Activity of UCART19, an Allogeneic Anti-CD19 CAR T-Cell Product, in a Pool of Adult and Pediatric Patients with High-Risk CD19+ Relapsed/Refractory B-Cell Acute Lymphoblastic Leukemia. Blood *132*, 896. https://doi.org/10.1182/blood-2018-99-111356.

79. Cichocki, F., Bjordahl, R., Gaidarova, S., Mahmood, S., Abujarour, R., Wang, H., Tuininga, K., Felices, M., Davis, Z.B., Bendzick, L., et al. (2020). iPSC-derived NK cells maintain high cytotoxicity and enhance in vivo tumor control in concert with T cells and anti–PD-1 therapy. Sci. Transl. Med. *12*, eaaz5618. https://doi.org/10.1126/scitranslmed.aaz5618.

80. Liu, E., Marin, D., Banerjee, P., Macapinlac, H.A., Thompson, P., Basar, R., Nassif Kerbauy, L., Overman, B., Thall, P., Kaplan, M., et al. (2020). Use of CAR-Transduced Natural Killer Cells in CD19-Positive Lymphoid Tumors. N. Engl. J. Med. *382*, 545–553. https://doi.org/10.1056/nejmoa1910607.

81. Benjamin, R., Jain, N., Maus, M.V., Boissel, N., Graham, C., Jozwik, A., Yallop, D., Konopleva, M., Frigault, M.J., Teshima, T., et al. (2022). UCART19, a first-in-class allogeneic anti-CD19 chimeric antigen receptor T-cell therapy for adults with relapsed or refractory B-cell acute lymphoblastic leukaemia (CALM): a phase 1, dose-escalation trial. Lancet Haematol. *9*, e833–e843. https://doi.org/10.1016/S2352-3026(22)00245-9.

82. Mailankody, S., Matous, J.V., Chhabra, S., Liedtke, M., Sidana, S., Oluwole, O.O., Malik, S., Nath, R., Anwer, F., Cruz, J.C., et al. (2023). Allogeneic BCMA-targeting CAR T cells in relapsed/refractory multiple myeloma: phase 1 UNIVERSAL trial interim results. Nat. Med. *29*, 422–429. https://doi.org/10.1038/s41591-022-02182-7.

83. Nahmad, A.D., Reuveni, E., Goldschmidt, E., Tenne, T., Liberman, M., Horovitz-Fried, M., Khosravi, R., Kobo, H., Reinstein, E., Madi, A., et al. (2022). Frequent aneuploidy in primary human T cells after CRISPR-Cas9 cleavage. Nat. Biotechnol. *40*, 1807–1813. https://doi.org/10.1038/s41587-022-01377-0.

84. Depil, S., Duchateau, P., Grupp, S.A., Mufti, G., and Poirot, L. (2020). "Off-the-shelf" allogeneic CAR T cells: development and challenges. Nat. Rev. Drug Discov. *19*, 185–199. https://doi.org/10.1038/s41573-019-0051-2.

85. Zhou, Y., Li, M., Zhou, K., Brown, J., Tsao, T., Cen, X., Husman, T., Bajpai, A., Dunn, Z.S., and Yang, L. (2022). Engineering Induced Pluripotent Stem Cells for Cancer Immunotherapy. Cancers *14*, 2266. https://doi.org/10.3390/cancers14092266.

86. Zhu, H., Blum, R.H., Bjordahl, R., Gaidarova, S., Rogers, P., Lee, T.T., Abujarour, R., Bonello, G.B., Wu, J., Tsai, P.-F., et al. (2020). Pluripotent stem cell-derived NK cells with high-affinity noncleavable CD16a mediate improved antitumor activity. Blood *135*, 399–410. https://doi.org/10.1182/blood.2019000621.

87. Cichocki, F., Goodridge, J.P., Bjordahl, R., Gaidarova, S., Mahmood, S., Abujarour, R., Davis, Z., Wang, H., Tuininga, K., Kodal, B., et al. (2021). Off-the-Shelf, Multiplexed-Engineered iPSC-Derived NK Cells Mediate Potent Multi-Antigen Targeting of B-Cell Malignancies with Reduced Cytotoxicity Against Healthy B Cells. Blood *138*, 407. https://doi.org/10.1182/blood-2021-148654.

88. Furukawa, Y., Ishii, M., Ando, J., Ikeda, K., Igarashi, K.J., Kinoshita, S., Azusawa, Y., Toyota, T., Honda, T., Nakanishi, M., et al. (2023). iPSC-derived hypoimmunogenic tissue resident memory T cells mediate robust anti-tumor activity against cervical cancer. Cell Rep. Med. *4*, 101327. https://doi.org/10.1016/j.xcrm.2023.101327.

89. Presidential Symposium and Presentation of Top Abstracts (2023). Mol. Ther. *31*, 1–794. https://doi.org/10.1016/j.ymthe.2023.04.017.

90. Rotolo, A., Whelan, E.C., Atherton, M.J., Kulikovskaya, I., Jarocha, D., Fraietta, J.A., Kim, M.M., Diffenderfer, E.S., Cengel, K.A., Piviani, M., et al. (2023). Unedited allogeneic iNKT cells show extended persistence in MHC-mismatched canine recipients. Cell Rep. Med. *4*, 101241. https://doi.org/10.1016/j.xcrm.2023.101241.

91. Lemoine, J., Ruella, M., and Houot, R. (2021). Born to survive: how cancer cells resist CAR T cell therapy. J. Hematol. Oncol. *14*, 199. https://doi.org/10.1186/s13045-021-01209-9.

92. Tahmasebi, S., Elahi, R., and Esmaeilzadeh, A. (2019). Solid Tumors Challenges and New Insights of CAR T Cell Engineering. Stem Cell Rev. Rep. *15*, 619–636. https://doi.org/10.1007/s12015-019-09901-7.

93. Li, C., Xu, X., Wei, S., Jiang, P., Xue, L., and Wang, J.; Senior Correspondence (2021). Tumor-associated macrophages: potential therapeutic strategies and future prospects in cancer. J. Immunother. Can. *9*, e001341. https://doi.org/10.1136/jitc-2020-001341.

94. Xia, Y., Rao, L., Yao, H., Wang, Z., Ning, P., and Chen, X. (2020). Engineering Macrophages for Cancer Immunotherapy and Drug Delivery. Adv. Mater. *32*, e2002054. https://doi.org/10.1002/adma.202002054.

95. Li, X., Yao, W., Yuan, Y., Chen, P., Li, B., Li, J., Chu, R., Song, H., Xie, D., Jiang, X., and Wang, H. (2017). Targeting of tumour-infiltrating macrophages via CCL2/CCR2 signalling as a therapeutic strategy against hepatocellular carcinoma. Gut *66*, 157–167. https://doi.org/10.1136/gutjnl-2015-310514.

96. Rodriguez-Garcia, A., Lynn, R.C., Poussin, M., Eiva, M.A., Shaw, L.C., O'Connor, R.S., Minutolo, N.G., Casado-Medrano, V., Lopez, G., Matsuyama, T., and Powell, D.J., Jr. (2021). CAR-T cell-mediated depletion of immunosuppressive tumor-associated macrophages promotes endogenous antitumor immunity and augments adoptive immunotherapy. Nat. Commun. *12*, 877. https://doi.org/10.1038/s41467-021-20893-2.

97. Xu, X., Huang, W., Heczey, A., Liu, D., Guo, L., Wood, M., Jin, J., Courtney, A.N., Liu, B., Di Pierro, E.J., et al. (2019). NKT Cells Coexpressing a GD2-Specific Chimeric Antigen Receptor and IL15 Show Enhanced In Vivo Persistence and Antitumor Activity against Neuroblastoma. Clin. Cancer Res. *25*, 7126–7138. https://doi.org/10.1158/1078-0432.CCR-19-0421.

98. Straathof, K.C., Pulè, M.A., Yotnda, P., Dotti, G., Vanin, E.F., Brenner, M.K., Heslop, H.E., Spencer, D.M., and Rooney, C.M. (2005). An inducible caspase 9 safety switch for T-cell therapy. Blood *105*, 4247–4254. https://doi.org/10.1182/blood-2004-11-4564.

99. Giannoni, F., Hardee, C.L., Wherley, J., Gschweng, E., Senadheera, S., Kaufman, M.L., Chan, R., Bahner, I., Gersuk, V., Wang, X., et al. (2013). Allelic exclusion and peripheral reconstitution by TCR transgenic T cells arising from transduced human hematopoietic stem/progenitor cells. Mol. Ther. *21*, 1044–1054. https://doi.org/10.1038/mt.2013.8.

100. Smith, D.J., Liu, S., Ji, S., Li, B., McLaughlin, J., Cheng, D., Witte, O.N., and Yang, L. (2015). Genetic engineering of hematopoietic stem cells to generate invariant natural killer T cells. Proc. Natl. Acad. Sci. USA *112*, 1523–1528. https://doi.org/10.1073/pnas.1424877112.

101. Ramkumar, P., Abarientos, A.B., Tian, R., Seyler, M., Leong, J.T., Chen, M., Choudhry, P., Hechler, T., Shah, N., Wong, S.W., et al. (2020). CRISPR-based screens uncover determinants of immunotherapy response in multiple myeloma. Blood Adv. *4*, 2899–2911. https://doi.org/10.1182/bloodadvances.2019001346.

102. Seet, C.S., He, C., Bethune, M.T., Li, S., Chick, B., Gschweng, E.H., Zhu, Y., Kim, K., Kohn, D.B., Baltimore, D., et al. (2017). Generation of mature T cells from human hematopoietic stem and progenitor cells in artificial thymic organoids. Nat. Methods *14*, 521–530. https://doi.org/10.1038/nmeth.4237.

YMTHE, Volume *32*

Supplemental Information

Engineering allorejection-resistant CAR-NKT cells from hematopoietic stem cells for off-the-shelf cancer immunotherapy

Yan-Ruide Li, Yang Zhou, Jiaji Yu, Yichen Zhu, Derek Lee, Enbo Zhu, Zhe Li, Yu Jeong Kim, Kuangyi Zhou, Ying Fang, Zibai Lyu, Yuning Chen, Yanxin Tian, Jie Huang, Xinjian Cen, Tiffany Husman, Jae Min Cho, Tzung Hsiai, Jin J. Zhou, Pin Wang, Benjamin R. Puliafito, Sarah M. Larson, and Lili Yang

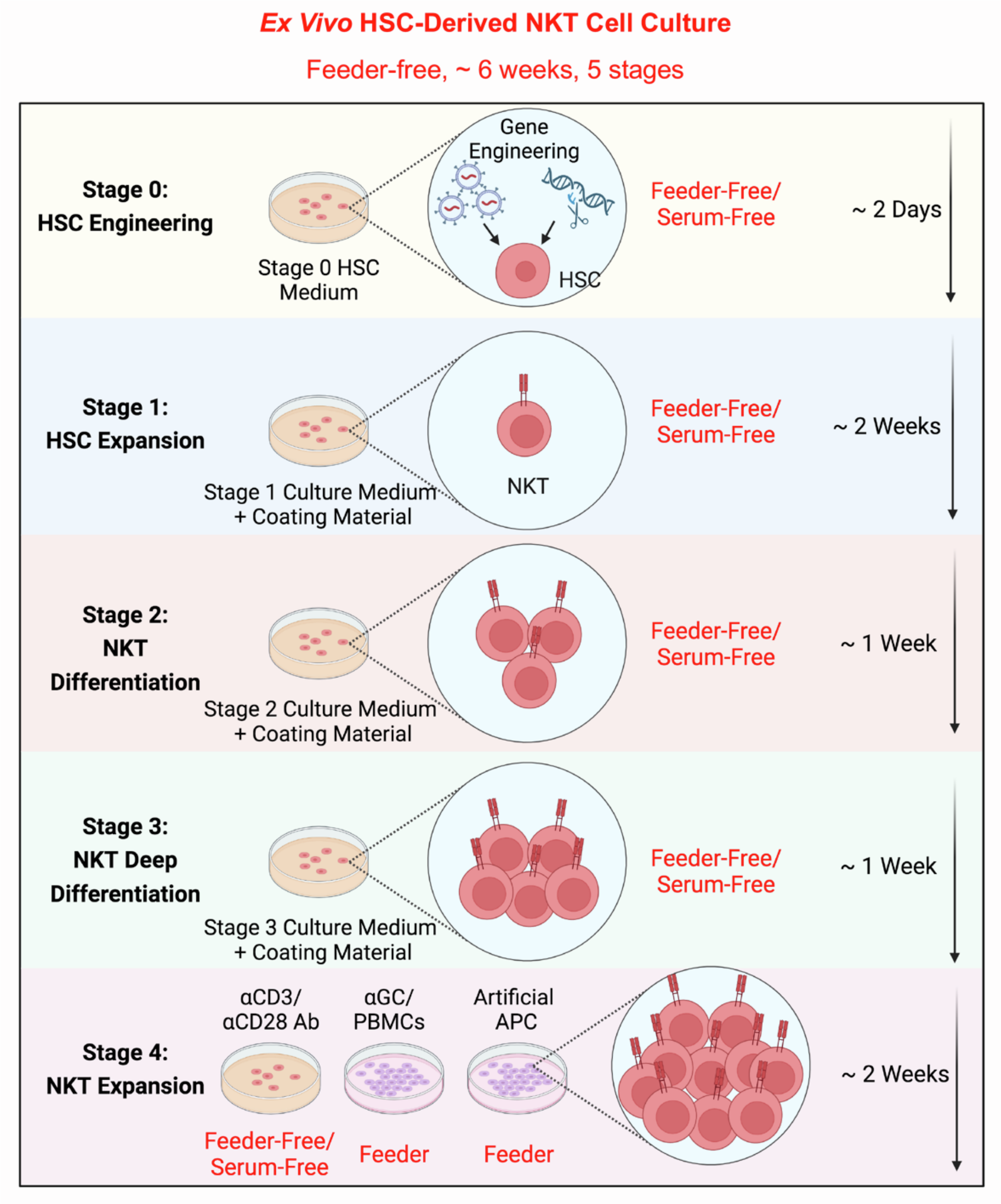

Figure S1. Diagram of the *Ex Vivo* HSC-Derived NKT Cell Culture.
Cryopreserved human cord blood-derived $CD34^{+}$ hematopoietic stem and progenitor cells (referred to as HSCs) were used to generate NKT cell products through a 5-stage, 6-week process. At Stage 0, HSCs were transduced with a lentivector, electroporated with a CRISPR-Cas9/B2M-CIITA-gRNAs complex, and then cultured over 48 hours in a classical X-VIVO 15-based HSC Medium. Gene-engineered HSCs were then cultured over ~6 weeks to generate a designated CAR-NKT cell product: Stage 1 HSC expansion (~2 weeks), Stage 2 NKT differentiation (~1 week), Stage 3 NKT deep differentiation (~1 week), and Stage 4 NKT expansion (~2 weeks). The Stage 1 Culture Medium comprised the StemSpan™ SFEM II Medium (SFEM) and the StemSpan™ Lymphoid Progenitor Expansion Supplement to support the HSC expansion. The Stage 2 Culture Medium comprised the SFEM and the StemSpan™ Lymphoid Progenitor Maturation Supplement (LPMS) to support the NKT cell differentiation. The Stage 3 Culture Medium comprised the SFEM, the LPMS, the CD3/CD28/CD2 T Cell Activator, and the human

recombinant IL-15 to support NKT cell deep differentiation. In addition, the StemSpan™ Lymphoid Differentiation Coating Material were utilized throughout Stages 1 to 3 to support HSC expansion and differentiation into T cell lineage. The entire 5-stage culture can be implemented in a feeder-free and serum-free manner. Alternatively, two feeder-dependent strategies involving α-galactosylceramide (αGC)-loaded healthy donor PBMCs or K562-based artificial antigen-presenting cells (aAPCs) can be employed for Stage 4 NKT expansion.

A

Cell products	Engineering strategy		Description
	Lentivector design	CRISPR-Cas9 editing	
Allo15BCAR-NKT	Lenti/iNKT-BCAR-IL-15	NA	Allogeneic IL-15-enhanced BCMA-targeting CAR-engineered NKT cells
U15BCAR-NKT	Lenti/iNKT-BCAR-IL-15	*B2M* and *CIITA* KO	HLA-ablated universal IL-15-enhanced BCMA-targeting CAR-engineered NKT cells
Allo15BCAR-NKT/FG	Lenti/iNKT-BCAR-IL-15 and Lenti/FG	NA	Allogeneic IL-15-enhanced BCMA-targeting CAR-engineered NKT cells labeled with FG
U15BCAR-NKT/FG	Lenti/iNKT-BCAR-IL-15 and Lenti/FG	*B2M* and *CIITA* KO	HLA-ablated universal IL-15-enhanced BCMA-targeting CAR-engineered NKT cells labeled with FG
UBCAR-NKT-TK	Lenti/iNKT-BCAR-sr39TK	*B2M* and *CIITA* KO	HLA-ablated universal BCMA-targeting CAR-engineered NKT cells with sr39TK
UEBCAR-NKT	Lenti/iNKT-BCAR-HLA-E	*B2M* and *CIITA* KO	HLA-ablated universal HLA-E-overexpressed BCMA-targeting CAR-engineered NKT cells

B

Lentivectors	Titers (IFU/ml)
Lenti/iNKT-BCAR-IL-15	$1.784 \times 10^8 \pm 5.61 \times 10^7$
Lenti/iNKT-BCAR-sr39TK	$1.623 \times 10^8 \pm 2.49 \times 10^7$
Lenti/iNKT-BCAR-HLA-E	$1.692 \times 10^8 \pm 4.28 \times 10^7$

C

Cell products	VCN
Allo15BCAR-NKT	3.40 ± 0.05
U15BCAR-NKT	3.27 ± 0.13
UEBCAR-NKT	3.19 ± 0.27

D

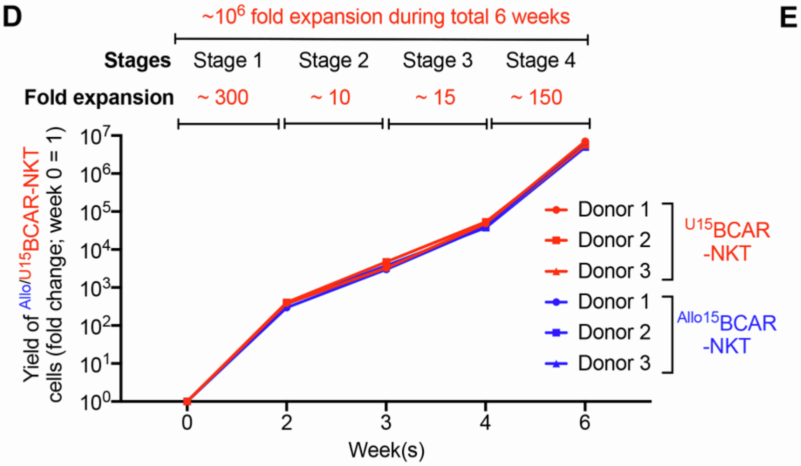

E

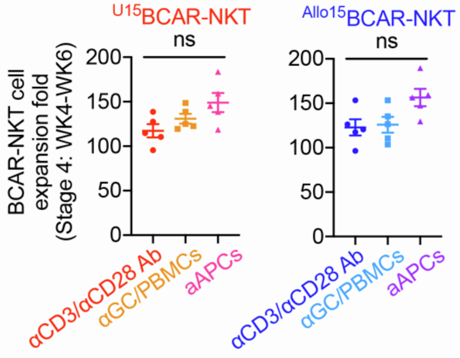

F

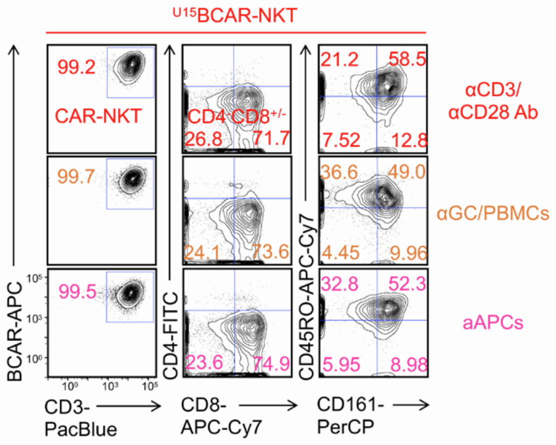

G

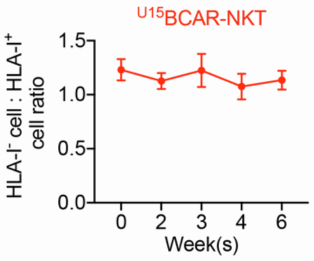

Figure S2. Summary of CAR-NKT cell products generated in this study and the generation of $^{Allo/U15}$BCAR-NKT cells.
(A) Table summarizing the generated CAR-NKT cell products in this study.
(B) Titers of the three lentivectors used in this study. Vector titers were measured by transducing HEK 293T cells with serial dilutions and performing flow cytometry (n = 3-6). IFU, infectious units.
(C) Vector copy numbers (VCNs) in the three indicated cell products. The generated BCAR-NKT cells were collected and analyzed for average VCN per cell using droplet digital PCR (ddPCR) (n = 3-6).
(D) Yield of $^{Allo/U15}$BCAR-NKT cells during the *Ex Vivo* HSC-Derived NKT Cell Culture.
(E) Comparison of $^{Allo/U15}$BCAR-NKT cell expansion fold using the three expansion approaches (n = 5).
(F) FACS detection of surface markers on ^{U15}BCAR-NKT cells generated using the three expansion approaches.
(G) The ratio of HLA-I/II-ablated ^{U15}BCAR-NKT cells to non-gene-edited cells during the *Ex Vivo* HSC-Derived NKT Cell Culture (n = 5).
Representative of > 3 experiments. Data are presented as the mean ± SEM. ns, not significant, by one-way ANOVA.

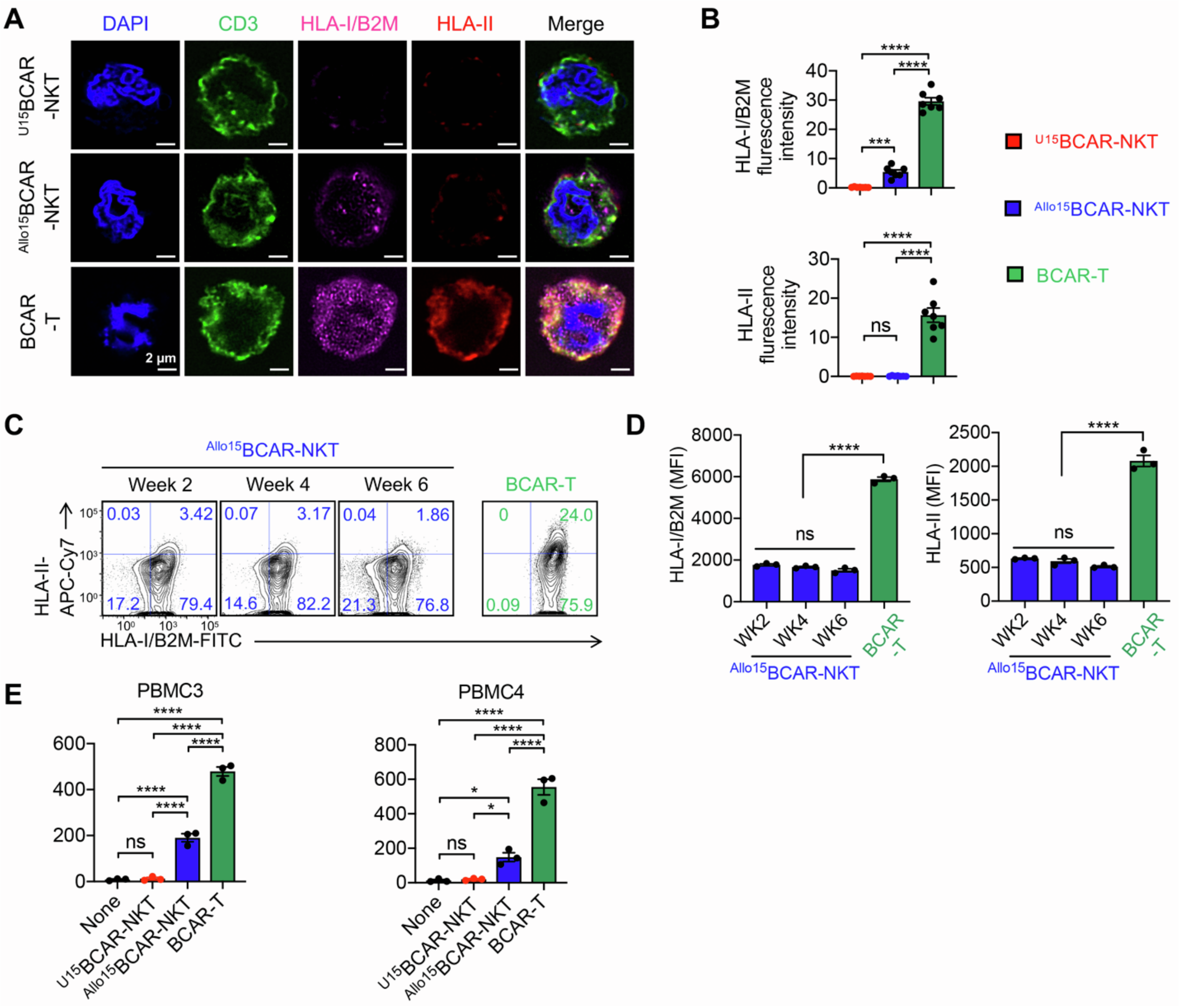

Figure S3. ^{U15}BCAR-NKT cells display an HLA-negative phenotype and resist to T cell-mediated allorejection, related to Figure 3.

(A-B) Studying HLA expression on $^{Allo/U15}$BCAR-NKT cells. (A) Immunofluorescence analysis of surface HLA-I/II on $^{Allo/U15}$BCAR-NKT cells. (B) Quantification of (A) (n = 7).

(C-D) Studying HLA expression ^{Allo15}BCAR-NKT cells throughout the 6-week cell culture. (C) FACS measurements of surface HLA-I/II molecules on ^{Allo15}BCAR-NKT cells collected from the indicated weeks. Conventional BCAR-T cells were included as a staining control. (D) Quantification of (C) (n = 3).

(E) ELISA analyses of IFN-γ production on day 4 in the T cell-mediated allorejection MLR assays (n = 3).

Representative of 3 experiments. Data are presented as the mean ± SEM. ns, not significant, *p < 0.05, ***p < 0.001, ****p < 0.0001, by one-way ANOVA.

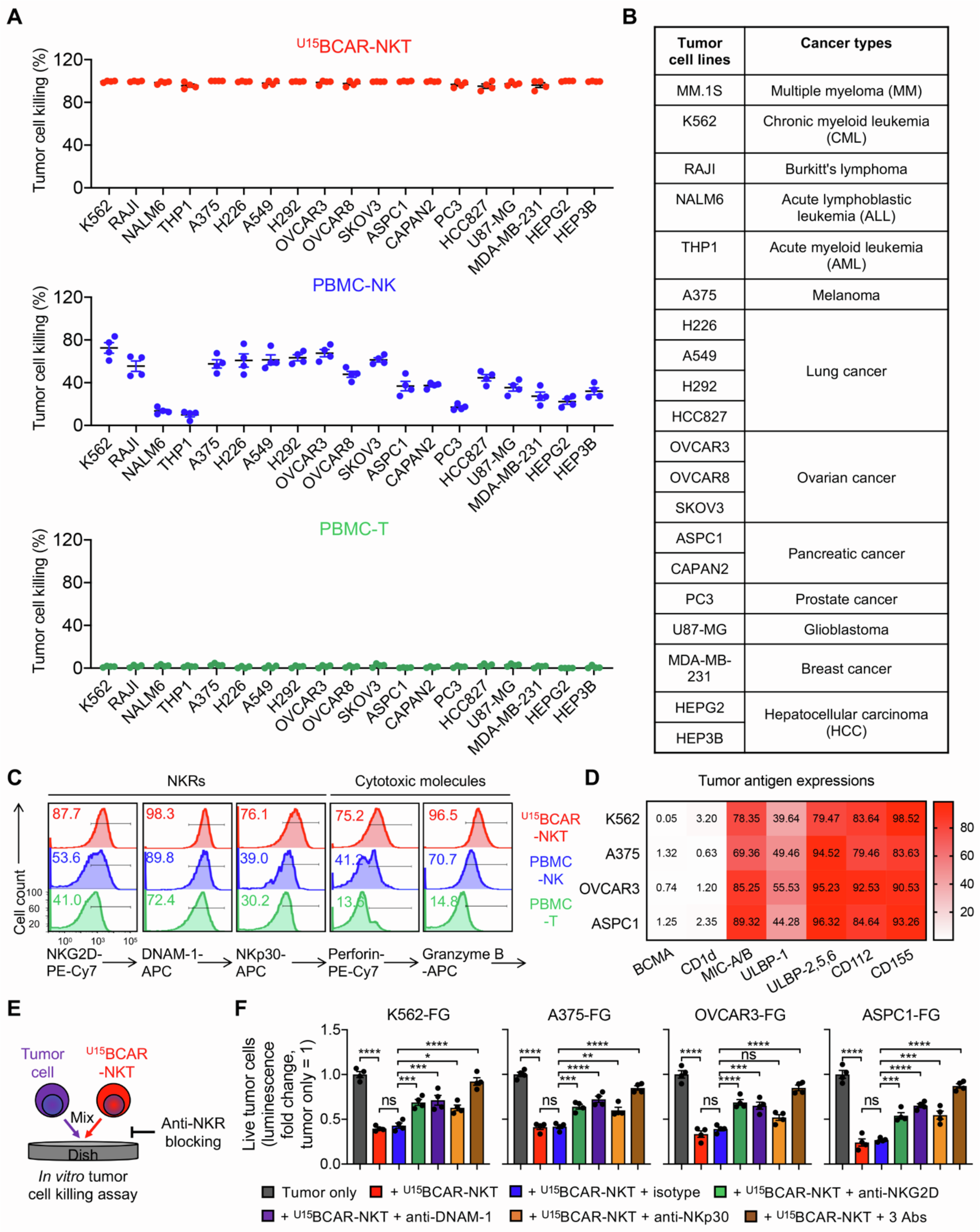

Figure S4. ***In vitro*** **tumor targeting of** ^{U15}**BCAR-NKT cells through intrinsic NK function, related to Figure 4.**

(A) Tumor cell killing data of ^{U15}BCAR-NKT cells at 24 h (n = 4). Healthy donor peripheral blood mononuclear cell (PBMC)-derived NK (PBMC-NK) and conventional T (PBMC-T) cells are included as therapeutic cell controls. E:T ratio = 10:1.
(B) Summary of tumor cell lines used in this study.
(C) FACS detection of surface NKR expression and intracellular cytotoxic molecules production by the indicated cells.
(D) FACS analyses of surface tumor antigen expressions on the indicated tumor cell lines. Data from 4 tumor cell lines (i.e., K562, A375, OVCAR3, and ASPC1) are presented. The numbers in the heatmap represent the percentage of antigen-positive tumor cells relative to the total tumor cell population. These percentages were determined based on control isotype staining.
(E-F) Studying the tumor killing mechanism of ^{U15}BCAR-NKT cells mediated by NKRs (i.e., NKG2D, DNAM-1, and NKp30). (E) Experimental design. (F) Tumor cell killing data at 24 h (E:T ratio = 2:1; n = 4).
Representative of 3 experiments. Data are presented as the mean ± SEM. ns, not significant, *$p < 0.05$, **$p < 0.01$, ***$p < 0.001$, ****$p < 0.0001$, by 1-way ANOVA.

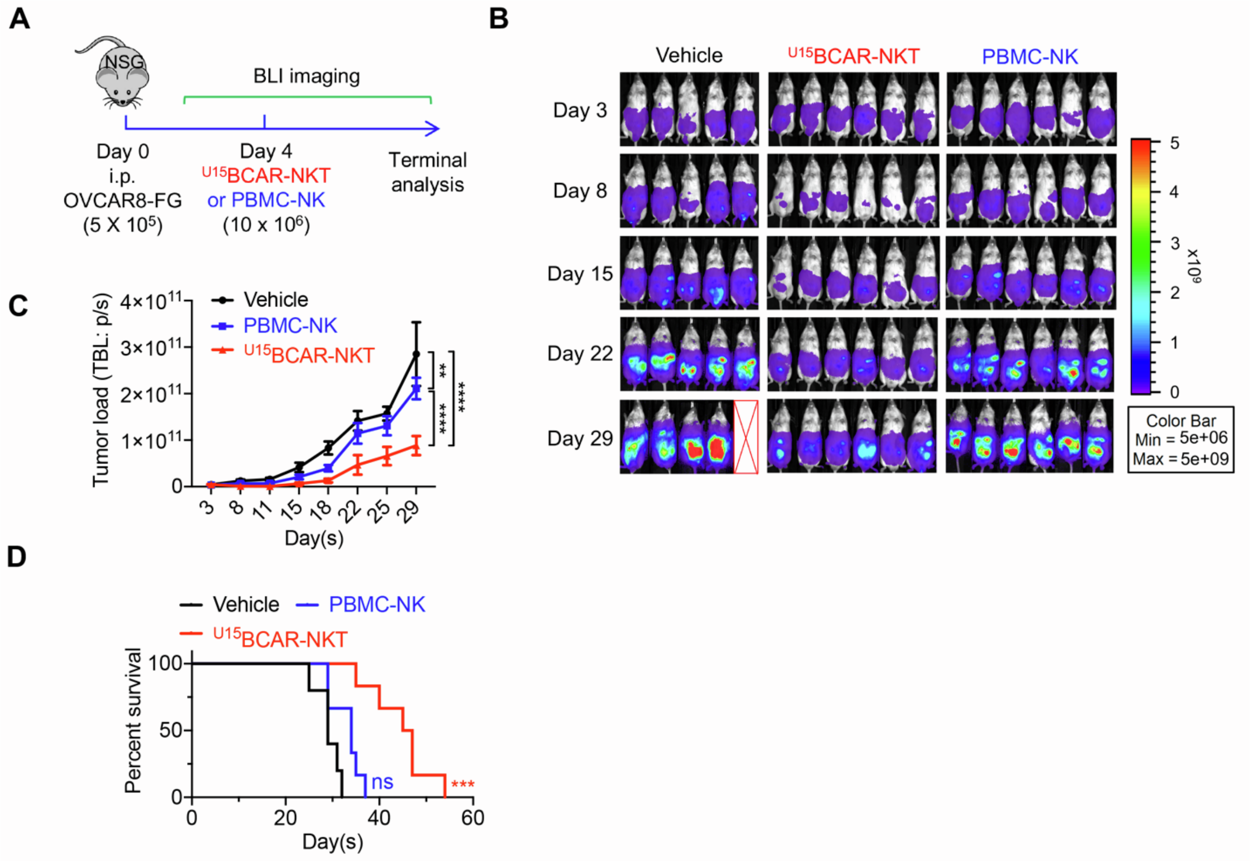

Figure S5. ***In vivo*** **tumor targeting of** **U15BCAR-NKT cells through intrinsic NK function, related to Figure 4.**
(A) Experimental design to study the *in vivo* antitumor efficacy of U15BCAR-NKT cells in an OVCAR8-FG xenograft NSG mouse model. PBMC-NK cells were included as a therapeutic cell control.
(B) BLI images showing tumor loads in experimental mice over time.
(C) Quantification of (B) (n = 5-6).
(D) Kaplan-Meier survival curves of experimental mice over time (n = 5-6).
Representative of 3 experiments. Data are presented as the mean ± SEM. ns, not significant, $^{**}p < 0.01$, $^{***}p < 0.001$, $^{****}p < 0.0001$, by 2-way ANOVA (C), or log rank (Mantel-Cox) test adjusted for multiple comparisons (D).

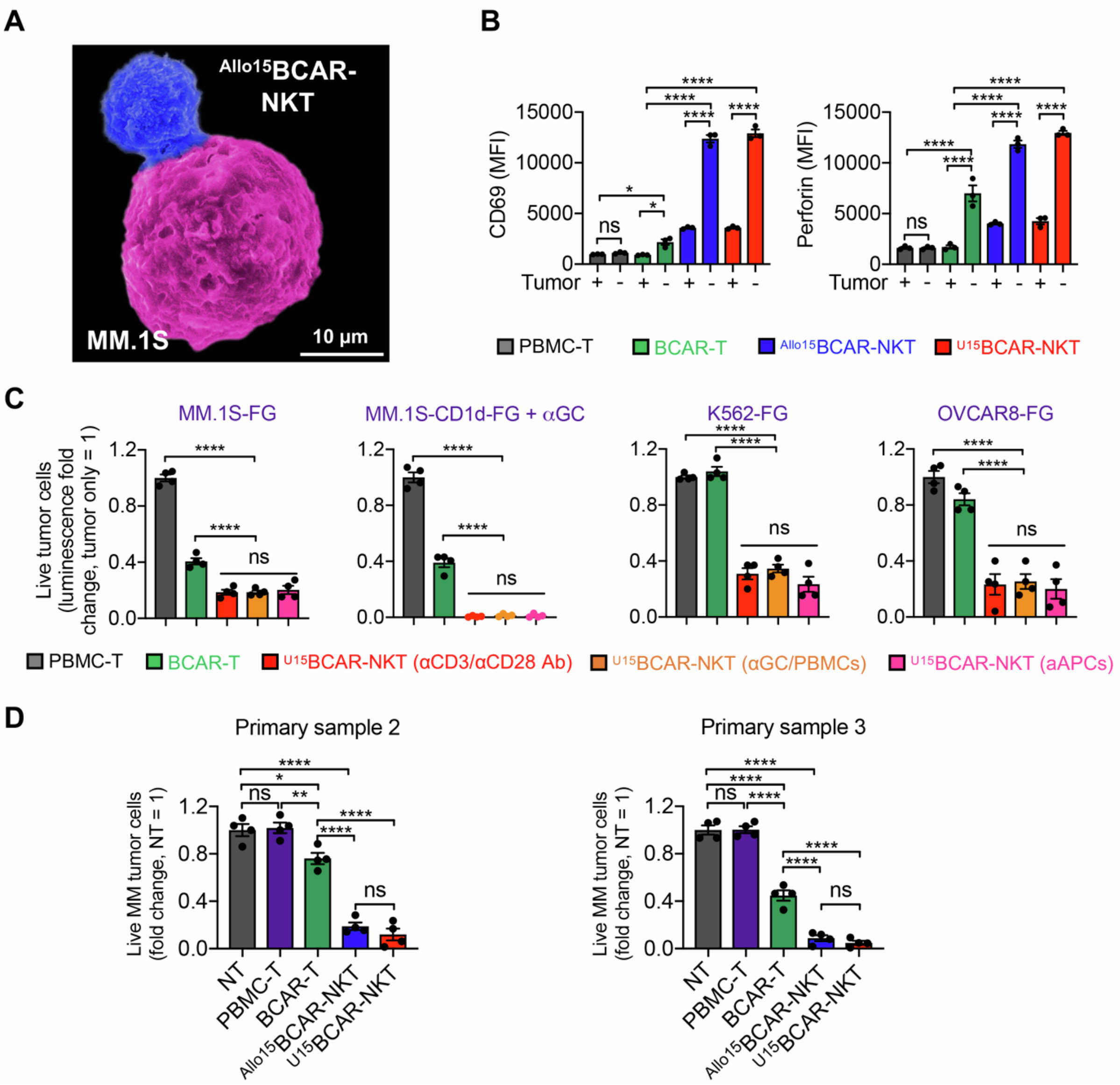

Figure S6. ***In vitro*** **antitumor efficacy and mechanism of action (MOA) study of U15BCAR-NKT cells, related to Figure 4.**

(A) Scanning electron microscope (SEM) image showing that an Allo15BCAR-NKT cell (blue) is attacking an MM.1S tumor cell (purple).

(B) FACS analyses of surface activation marker (i.e., CD69) and intracellular cytotoxic molecule (i.e., Perforin) in the indicated cells after coculturing with MM.1S-FG cells for 24 hours (n = 3).

(C) Tumor cell killing data by the indicated therapeutic cells at 24 h (E:T ratio = 1:1; n = 4). U15BCAR-NKT cells generated using the three expansion approaches were compared.

(D) Primary MM tumor cell killing data by the indicated cells at 24 h (n = 4).

Representative of 3 experiments. Data are presented as the mean ± SEM. ns, not significant, *p < 0.05, **p < 0.01, ***p < 0.001, ****p < 0.0001, by 1-way ANOVA.

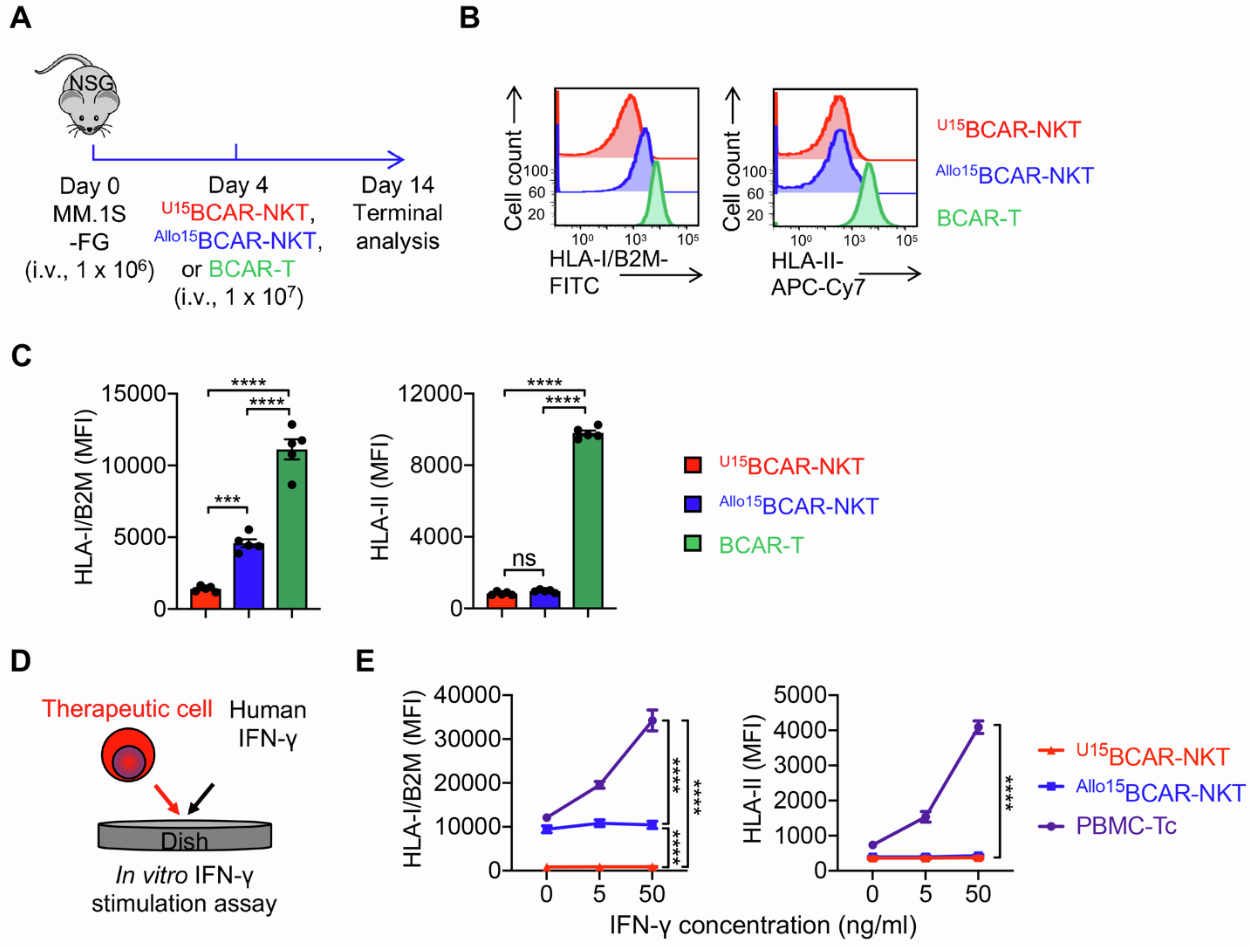

Figure S7. HLA expression of $^{Allo/U15}$BCAR-NKT cells *in vivo* or under IFN-γ stimulation, related to Figure 5.

(A-C) Studying HLA expression of $^{Allo/U15}$BCAR-NKT cells in a human MM.1S xenograft NSG mouse model. (A) Experimental design. Conventional BCAR-T cells were included as a control. (B) FACS measurements of surface HLA-I/II on $^{Allo/U15}$BCAR-NKT cells post *in vivo* antitumor response. (C) Quantification of (B) (n = 5).

(D-E) Studying HLA expression of $^{Allo/U15}$BCAR-NKT cells under IFN-γ stimulation. (D) Experimental design. $^{Allo/U15}$BCAR-NKT cells were stimulated with a range of IFN-γ (0, 5, and 50 ng/ml) for 3 days. PBMC-Tc cells were included as a positive control. (E) Quantification of HLA-I/II expressions on the indicated cells stimulated with IFN-γ of indicated concentrations (n = 3).

Representative of 3 experiments. Data are presented as the mean ± SEM. ns, not significant, ***p < 0.001, ****p < 0.0001, by 1-way ANOVA (C) or 2-way ANOVA (E).

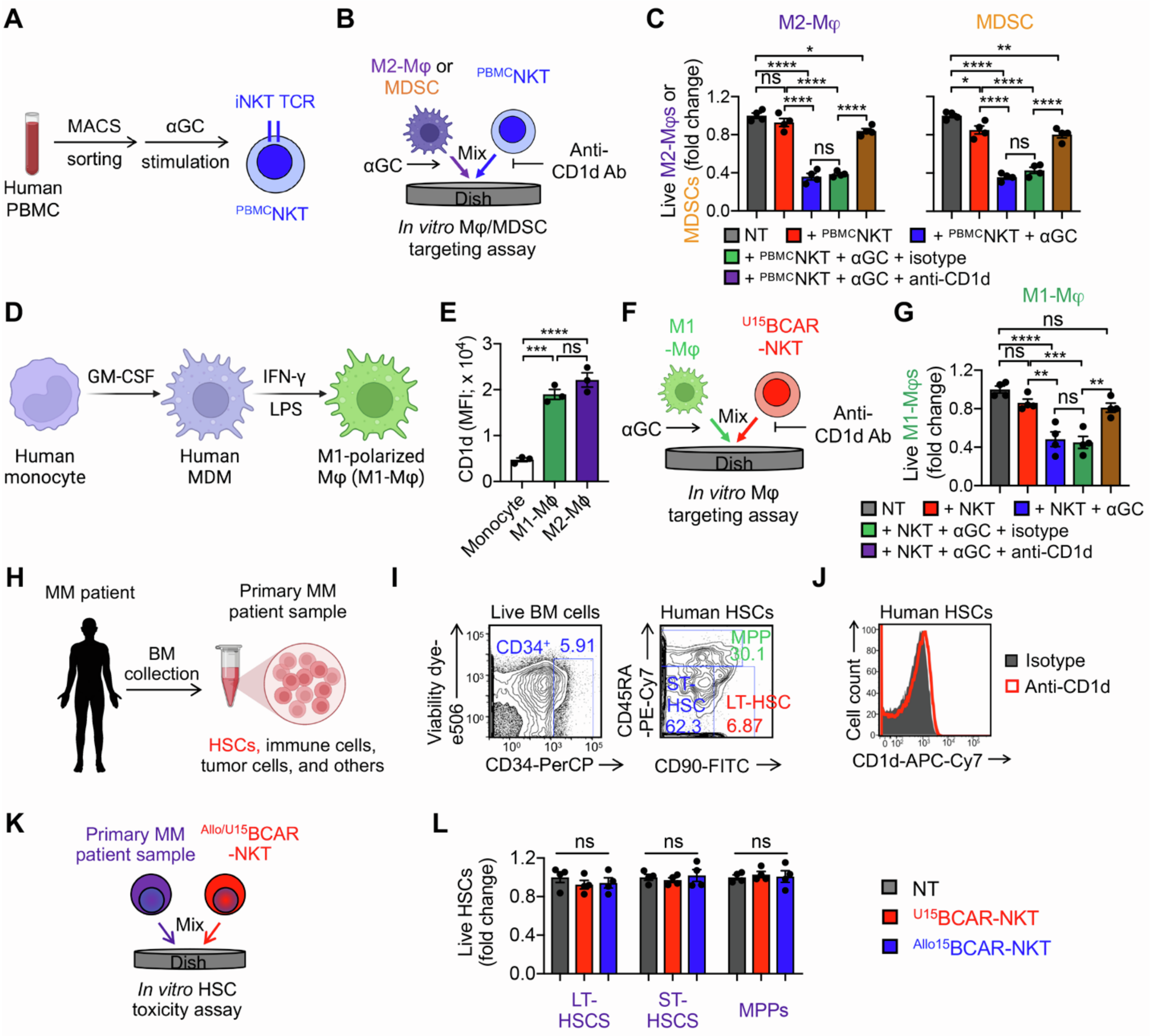

Figure S8. $^{Allo/U15}$BCAR-NKT cells selectively target the TME but not affect the human HSCs, related to Figure 7.

(A-C) Studying the TAM/MDSC targeting by PBMC-derived NKT (^{PBMC}NKT) cells. (A) Schematics showing the generation of ^{PBMC}NKT cells. (B) Experimental design to study Mφ/MDSC targeting by ^{PBMC}NKT cells using an *in vitro* Mφ/MDSC targeting assay. Mφ/MDSC:^{PBMC}NKT ratio = 1:1. (C) Mφ/MDSC killing data at 24 h (n = 4).

(D-G) Studying the M1-type macrophage (M1-Mφ) targeting by $^{Allo/U15}$BCAR-NKT cells. (D) Diagram showing the generation of healthy donor PBMC-derived M1-Mφs. (E) FACS detection of CD1d on the indicated cells. (F) Experimental design to study M1-Mφ targeting by $^{Allo/U15}$BCAR-NKT cells using an *in vitro* Mφ targeting assay. Mφ:NKT ratio = 1:1. (G) Mφ killing data at 24 h (n = 4).

(H-L) Studying the toxicity of $^{Allo/U15}$BCAR-NKT cells against human HSCs using primary MM patient BM samples. (H) Diagram showing the BM collection from primary MM patients. (I) FACS detection of long-term HSCs (LT-HSCs; gated as $CD34^+CD90^+CD45RA^-$ cells), short-term HSCs (ST-HSCs; gated as $CD34^+CD90^-CD45RA^-$ cells), and multi-potent progenitor cells (MPPs; gated as $CD34^+CD90^-CD45RA^+$ cells) in the MM patient BM cells. (J) FACS plots showing the lack of CD1d expression on primary MM patient BM HSCs. (K) Experimental design to study HSC killing

by $^{Allo/U15}$BCAR-NKT cells. MM patient BM cells were mixed with $^{Allo/U15}$BCAR-NKT cells at 1:1 ratio and cultured *in vitro*. (L) HSC killing data at 24 h (n = 4).
Representative of 3 experiments. Data are presented as the mean ± SEM. ns, not significant, *p < 0.05, **p < 0.01, ***p < 0.001, ****p < 0.0001, by 1-way ANOVA.

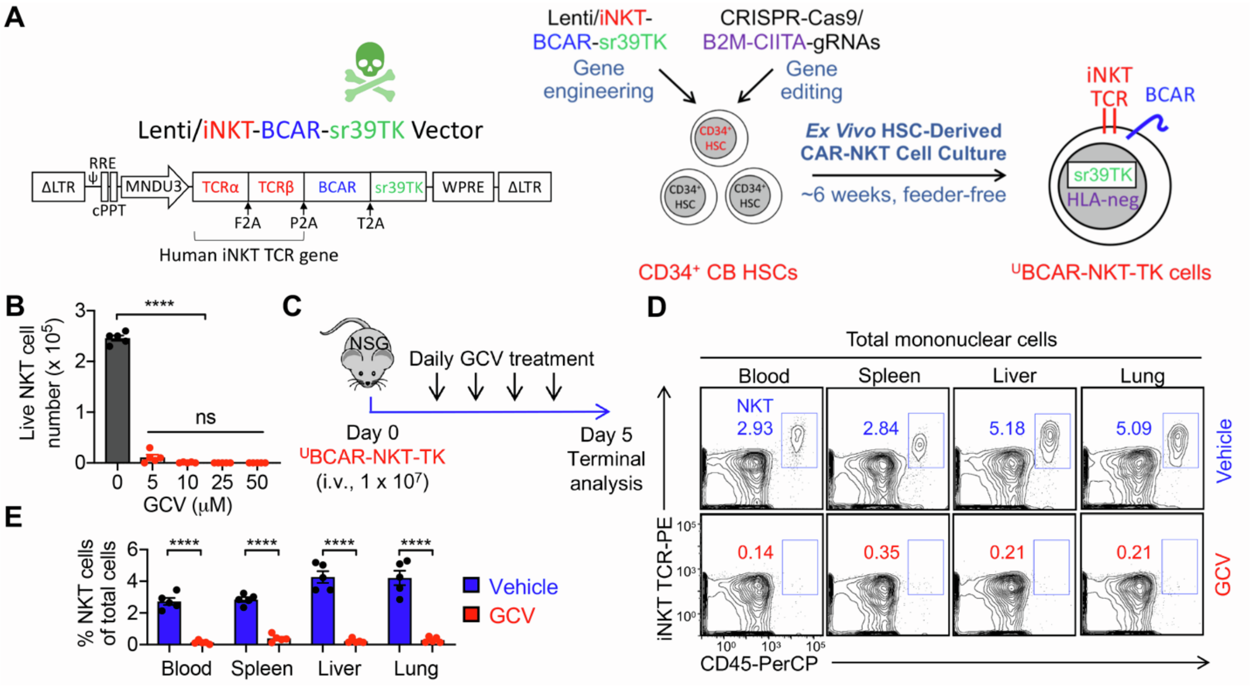

Figure S9. Incorporation of an sr39TK suicide switch in HSC-derived BCAR-NKT cells, related to Figure 8.

(A) Schematic showing the experimental design to generate HSC-engineered HLA-ablated BCAR-NKT cells equipped with an sr39TK suicide gene (denoted as ^{U}BCAR-NKT-TK cells). Lenti/iNKT-BCAR-sr39TK, lentivector encoding iNKT TCR, BCAR, and sr39TK.

(B) *In vitro* depletion of ^{U}BCAR-NKT-TK cells via GCV treatment (n = 4). GCV, ganciclovir that specifically depleting cells expressing the sr39TK gene.

(C-E) *In vivo* depletion of ^{U}BCAR-NKT-TK cells via GCV administration. (C) Experimental design. (D) FACS detection of ^{U}BCAR-NKT-TK cells in the indicated tissues of the experimental animals. (E) Quantification of (D).

Representative of 3 experiments. Data are presented as the mean ± SEM. ns, not significant; ****p < 0.0001 by Student's *t* test (E), or one-way ANOVA (B).

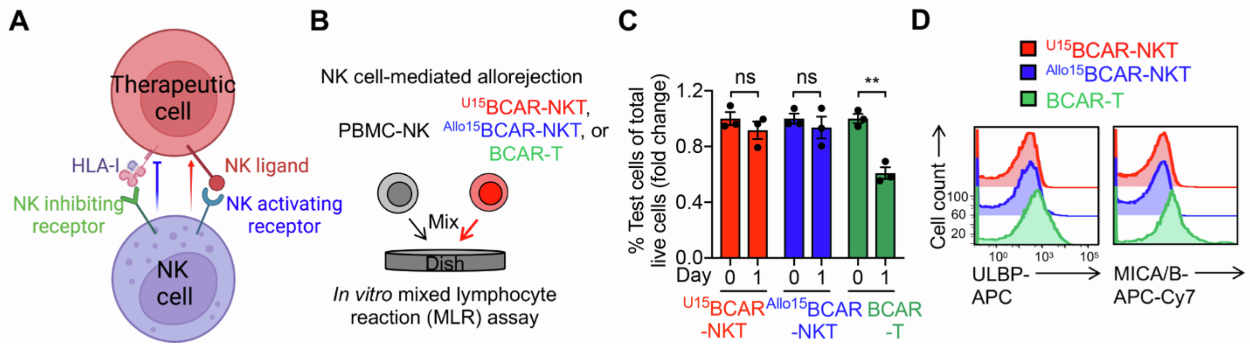

Figure S10. ^{U15}BCAR-NKT cells resist to NK cell-mediated allorejection.

(A) Diagram showing that host NK cells mediate allorejection through a double-trigger mechanism: 1) "missing self," which refers to the absence of matching HLA-I molecules on allogeneic cells, triggers the release of inhibitory signals mediated by NK inhibitory receptors, and 2) "stress signals," which refer to the upregulation of stress molecules on allogeneic cells, activate NK activating receptors such as NKG2D, DNAM-1, and NKp44.

(B-D) Studying the NK cell-mediated allorejection against ^{U15}BCAR-NKT cells using an *in vitro* MLR assay. PBMC-NK cells isolated from over ten random mismatched healthy donors were used. Data from one representative donor are presented. ^{Allo15}BCAR-NKT and BCAR-T cells were included as allorejection controls. (B) Experimental design. (C) FACS quantification of the indicated live cells on day 0 and day 1 (n = 3). (D) FACS measurements of NK ligands on the indicated therapeutic cells.

Representative of 3 experiments. Data are presented as the mean ± SEM. ns, not significant, $^{**}p < 0.01$, by Student's *t* test.

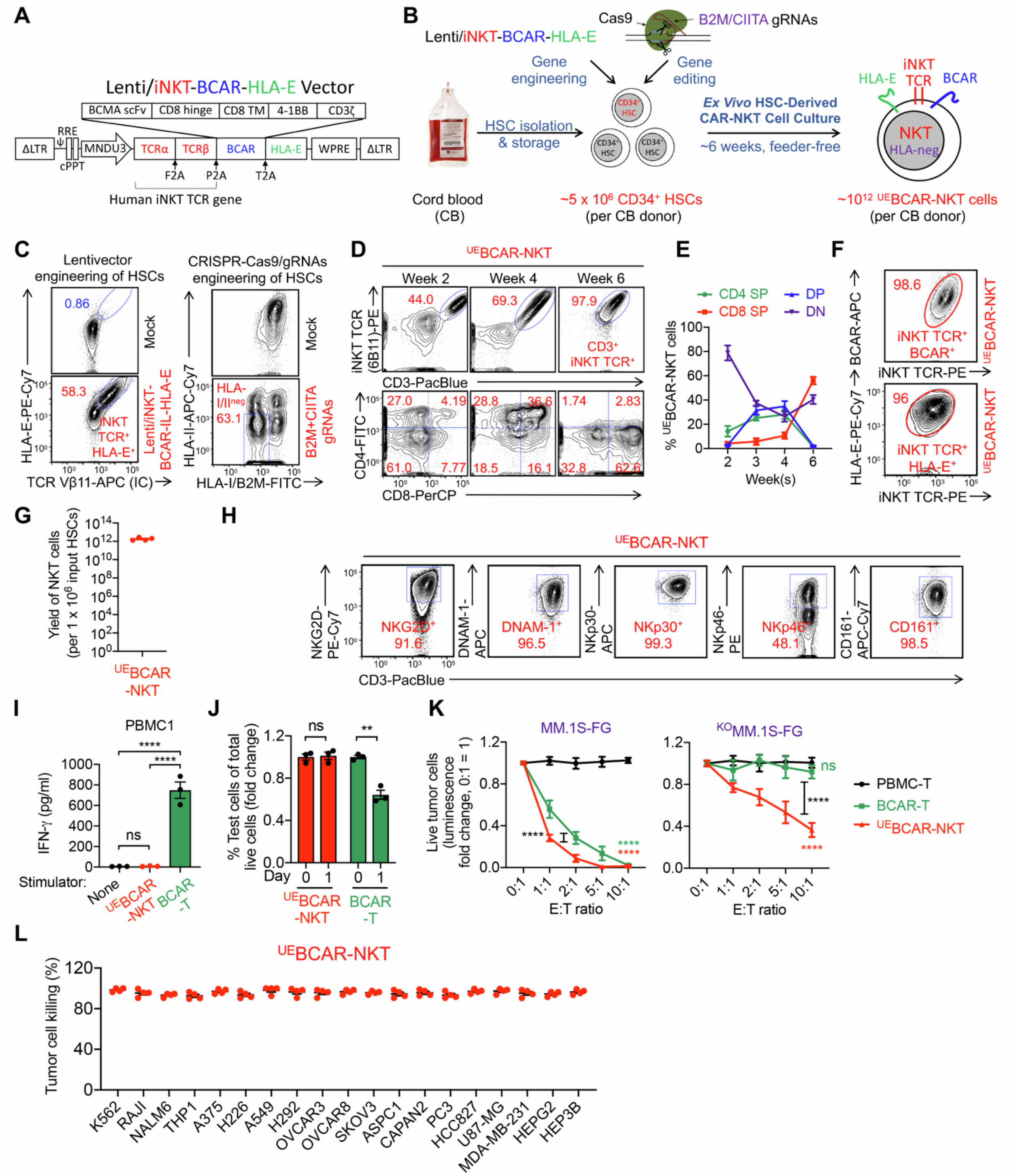

Figure S11. Generation of universal BCAR-NKT cells engineered with HLA-E to further ensure resistance to NK cell-mediated allorejection.

(A) Schematics showing the design of Lenti/iNKT-BCAR-HLA-E lentivector.

(B) Schematic showing the experimental design to generate HSC-engineered HLA-ablated BCAR-NKT cells equipped with an HLA-E gene (denoted as ^{UE}BCAR-NKT cells).

(C) Intracellular expression of iNKT TCR, surface expression of HLA-E, and surface ablation of HLA-I/II in CB HSCs 72 h after lentivector transduction and 48 h after CRISPR-Cas9 gene editing.

(D) FACS monitoring of the generation of ^{UE}BCAR-NKT cells.

(E) Quantification of the transition among four subpopulations of ^{UE}BCAR-NKT cells during their developmental stages.
(F) FACS detection of BCAR and HLA-E expressions on ^{UE}BCAR-NKT cells.
(G) Yield of ^{UE}BCAR-NKT cells (n = 4; n indicates different CB donors).
(H) FACS analyses of surface NK receptors on ^{UE}BCAR-NKT cells.
(I-J) Studying the T and NK cell-mediated allorejection against ^{UE}BCAR-NKT cells using *in vitro* MLR assays. BCAR-T cells were included as a control. (I) ELISA analyses of IFN-γ production on day 4 in the T cell-mediated allorejection assay (n = 3). (J) FACS quantification of the indicated live cells on day 0 and day 1 in the NK cell-mediated allorejection assay (n = 3).
(K-L) Tumor cell killing data by ^{UE}BCAR-NKT cells at 24 h (n = 4).
Representative of 3 experiments. Data are presented as the mean ± SEM. ns, not significant, *p < 0.05, **p < 0.01, ***p < 0.001, ****p < 0.0001, by Student's *t* test (J), 1-way ANOVA (I), or 2-way ANOVA (K).

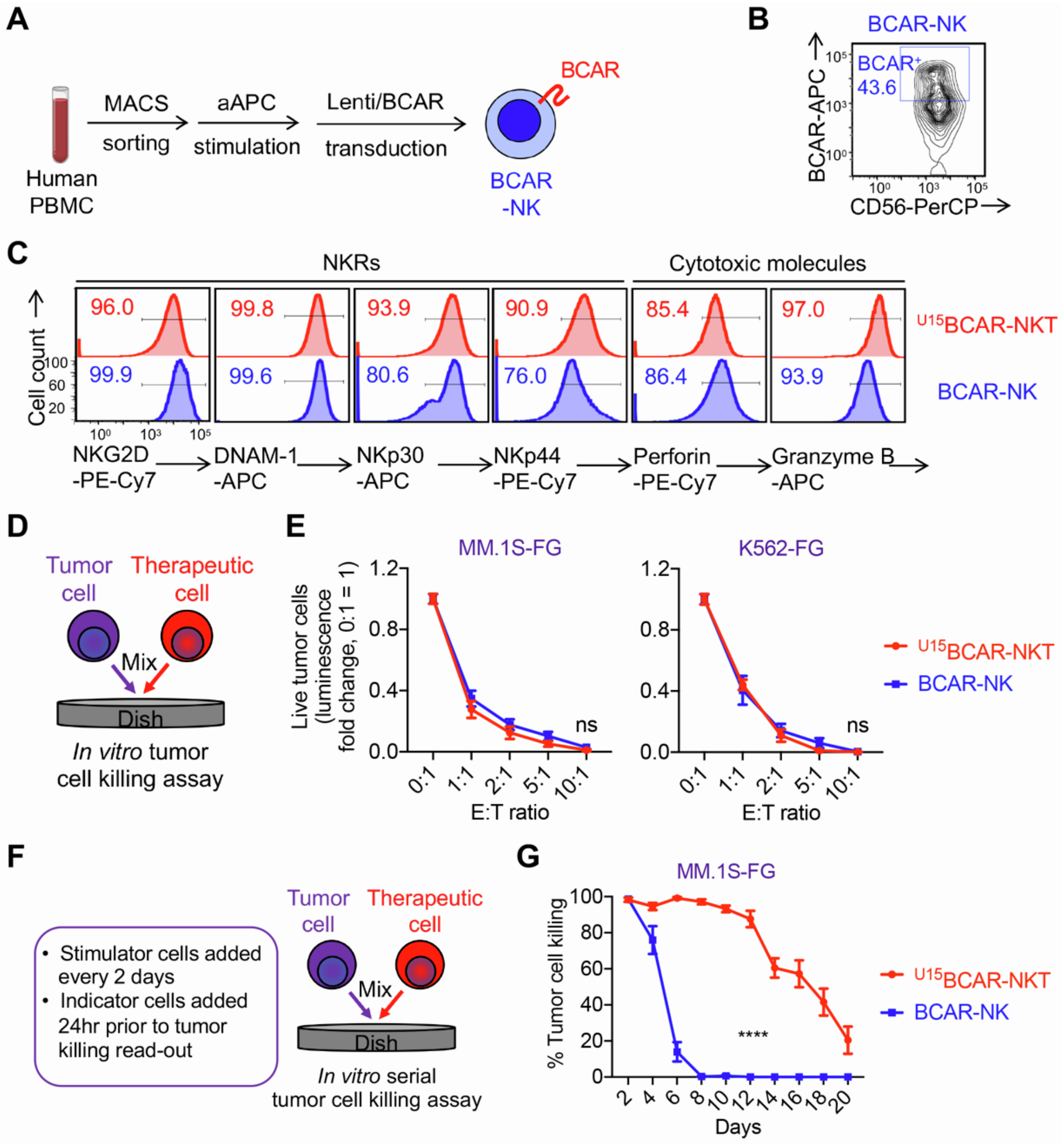

Figure S12. Comparison between ^{U15}BCAR-NKT cells and BCAR-engineered NK (BCAR-NK) cells.

(A) Schematics showing the generation of BCAR-NK cells.
(B) FACS plots showing the BCAR expression on BCAR-NK cells.
(C) FACS plots showing the surface NKR expression and intracellular cytotoxic molecule production of ^{U15}BCAR-NKT and BCAR-NK cells.
(D-E) Studying the *in vitro* antitumor efficacy of ^{U15}BCAR-NKT and BCAR-NK cells. (D) Experimental design. (E) Tumor cell killing data at 24 h (n = 4).
(F-G) Studying the long-term antitumor efficacy of ^{U15}BCAR-NKT and BCAR-NK cells using an *in vitro* serial tumor cell killing assay. (F) Experimental design. (G) Tumor cell killing data (n = 4).
Representative of two experiments. Data are presented as the mean ± SEM. ns, not significant, ****$p < 0.0001$, by two-way ANOVA.

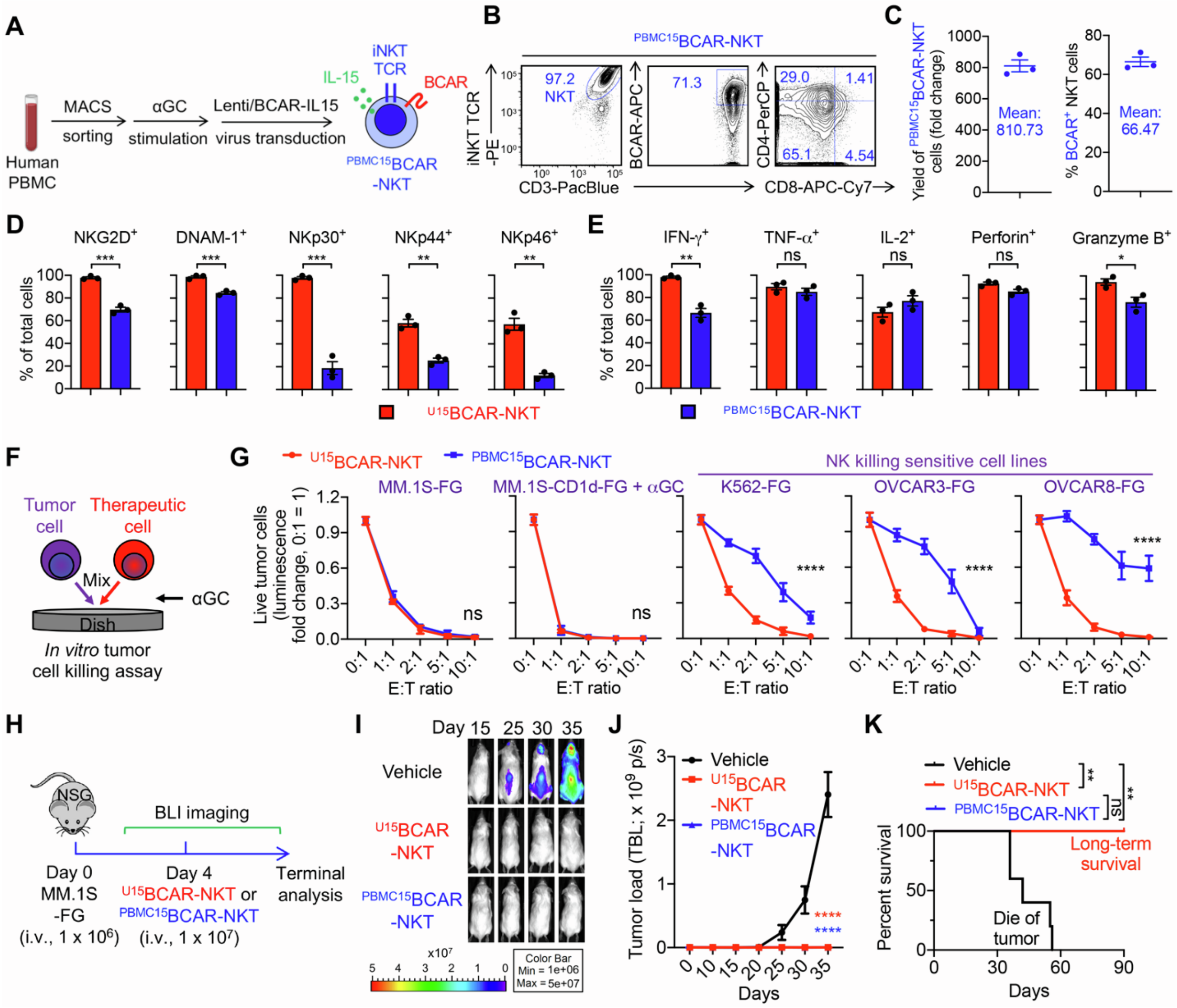

Figure S13. Comparison between U15BCAR-NKT cells and PBMC-derived IL-15-enhanced BCAR-engineered NKT (PBMC15BCAR-NKT) cells.
(A) Schematics showing the generation of PBMC15BCAR-NKT cells.
(B) FACS plots showing the purity, CAR expression, and CD4/CD8 co-receptor expression of PBMC15BCAR-NKT cells.
(C) Yield and CAR expression of PBMC15BCAR-NKT cells (n = 3; n indicates different donors).
(D) FACS analyses of surface NKR expressions on U15BCAR-NKT and PBMC15BCAR-NKT cells (n = 3).
(E) FACS analyses of intracellular cytokine and cytotoxic molecule production in U15BCAR-NKT and PBMC15BCAR-NKT cells (n = 3).
(F-G) Studying the *in vitro* antitumor efficacy of U15BCAR-NKT and PBMC15BCAR-NKT cells. (F) Experimental design. (G) Tumor cell killing data at 24 h (n = 4).
(H-K) Studying the *in vivo* antitumor efficacy of U15BCAR-NKT and PBMC15BCAR-NKT cells in a human MM xenograft NSG mouse model. (H) Experimental design. (I) BLI images showing the presence of tumor cells in experimental mice over time. (J) Quantification of (I) (n = 5). (K) Kaplan-Meier survival curves of experimental mice over time (n = 5).
Representative of three (A-G) and two (H-K) experiments. Data are presented as the mean ± SEM. ns, not significant; *$p < 0.05$; **$p < 0.01$; ***$p < 0.001$; ****$p < 0.0001$, by one-way ANOVA

(D and E), two-way ANOVA (G and J), or by log rank (Mantel-Cox) test adjusted for multiple comparisons (K).

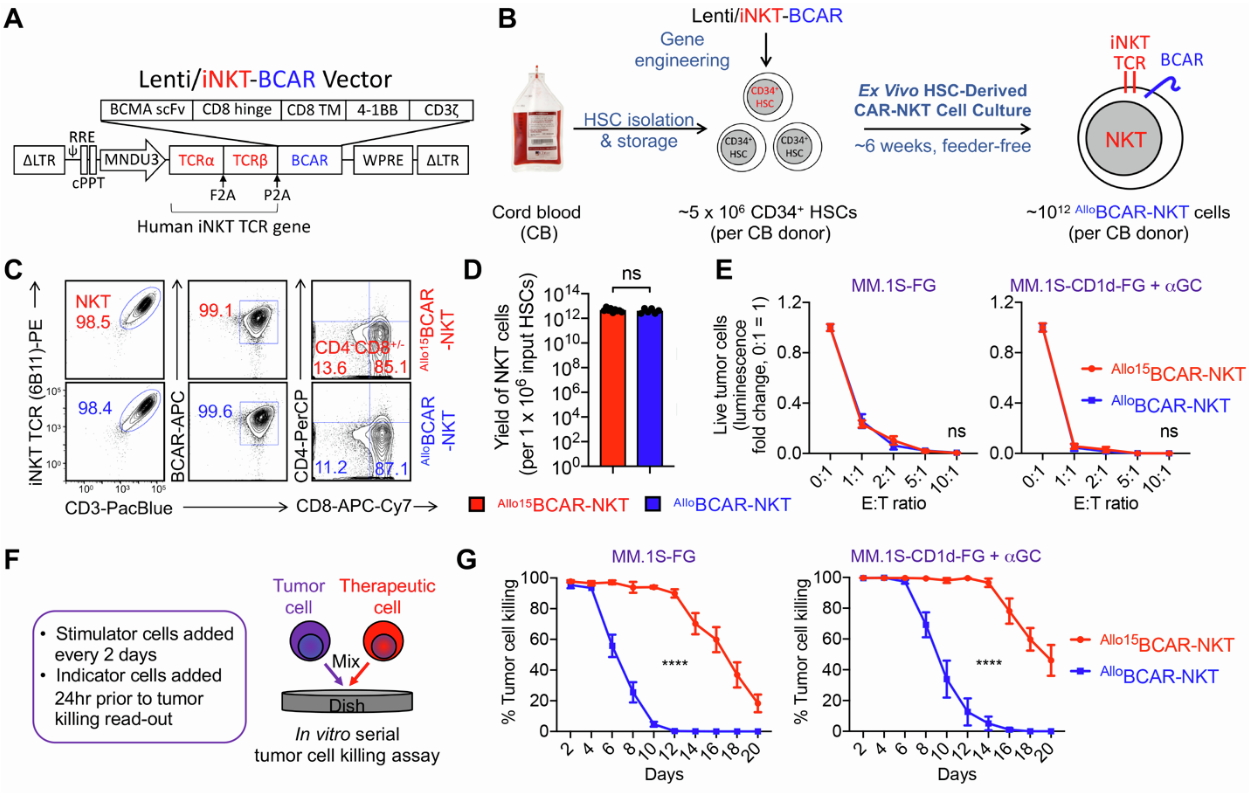

Figure S14. Comparison between non-IL-15-enhanced allogeneic BCAR-NKT (^{Allo}BCAR-NKT) cells with IL-15-enhanced ^{Allo15}BCAR-NKT cells.

(A) Schematics showing the design of Lenti/iNKT-BCAR lentivector.

(B) Schematic showing the experimental design to generate ^{Allo}BCAR-NKT cells.

(C) FACS detection of surface markers on $^{Allo/15}$BCAR-NKT cells.

(D) Yield of $^{Allo/15}$BCAR-NKT cells (n = 6-9; n indicates different donors).

(E) Tumor cell killing data by $^{Allo/15}$BCAR-NKT cells at 24 h (n = 4).

(F-G) Studying the long-term antitumor efficacy of $^{Allo/15}$BCAR-NKT cells using an *in vitro* serial tumor cell killing assay. (F) Experimental design. (G) Tumor cell killing data (n = 4).

Representative of 3 experiments. Data are presented as the mean ± SEM. ns, not significant, ****$p < 0.0001$, by Student's *t* test (D), or 2-way ANOVA (E and G).
